# Widespread alternative splicing dysregulation occurs presymptomatically in CAG expansion spinocerebellar ataxias

**DOI:** 10.1093/brain/awad329

**Published:** 2023-09-30

**Authors:** Hannah K Shorrock, Claudia D Lennon, Asmer Aliyeva, Emily E Davey, Cristina C DeMeo, Caroline E Pritchard, Lori Planco, Jose M Velez, Alexandra Mascorro-Huamancaja, Damian S Shin, John D Cleary, J Andrew Berglund

**Affiliations:** The RNA Institute, University at Albany—SUNY, Albany, NY 12222, USA; The RNA Institute, University at Albany—SUNY, Albany, NY 12222, USA; The RNA Institute, University at Albany—SUNY, Albany, NY 12222, USA; Department of Biology, University at Albany—SUNY, Albany, NY 12222, USA; The RNA Institute, University at Albany—SUNY, Albany, NY 12222, USA; The RNA Institute, University at Albany—SUNY, Albany, NY 12222, USA; The RNA Institute, University at Albany—SUNY, Albany, NY 12222, USA; The RNA Institute, University at Albany—SUNY, Albany, NY 12222, USA; The RNA Institute, University at Albany—SUNY, Albany, NY 12222, USA; Department of Biology, University at Albany—SUNY, Albany, NY 12222, USA; The RNA Institute, University at Albany—SUNY, Albany, NY 12222, USA; Department of Neuroscience and Experimental Therapeutics, Albany Medical College, Albany, NY 12208, USA; The RNA Institute, University at Albany—SUNY, Albany, NY 12222, USA; The RNA Institute, University at Albany—SUNY, Albany, NY 12222, USA; Department of Biology, University at Albany—SUNY, Albany, NY 12222, USA

**Keywords:** CAG repeat expansion, spinocerebellar ataxia, alternative splicing, polyglutamine diseases, RNA binding proteins

## Abstract

The spinocerebellar ataxias (SCAs) are a group of dominantly inherited neurodegenerative diseases, several of which are caused by CAG expansion mutations (SCAs 1, 2, 3, 6, 7 and 12) and more broadly belong to the large family of over 40 microsatellite expansion diseases. While dysregulation of alternative splicing is a well defined driver of disease pathogenesis across several microsatellite diseases, the contribution of alternative splicing in CAG expansion SCAs is poorly understood. Furthermore, despite extensive studies on differential gene expression, there remains a gap in our understanding of presymptomatic transcriptomic drivers of disease. We sought to address these knowledge gaps through a comprehensive study of 29 publicly available RNA-sequencing datasets.

We identified that dysregulation of alternative splicing is widespread across CAG expansion mouse models of SCAs 1, 3 and 7. These changes were detected presymptomatically, persisted throughout disease progression, were repeat length-dependent, and were present in brain regions implicated in SCA pathogenesis including the cerebellum, pons and medulla. Across disease progression, changes in alternative splicing occurred in genes that function in pathways and processes known to be impaired in SCAs, such as ion channels, synaptic signalling, transcriptional regulation and the cytoskeleton. We validated several key alternative splicing events with known functional consequences, including *Trpc3* exon 9 and *Kcnma1* exon 23b, in the *Atxn1^154Q/2Q^* mouse model. Finally, we demonstrated that alternative splicing dysregulation is responsive to therapeutic intervention in CAG expansion SCAs with *Atxn1* targeting antisense oligonucleotide rescuing key splicing events.

Taken together, these data demonstrate that widespread presymptomatic dysregulation of alternative splicing in CAG expansion SCAs may contribute to disease onset, early neuronal dysfunction and may represent novel biomarkers across this devastating group of neurodegenerative disorders.

## Teaser

Shorrock *et al*. show that changes to alternative splicing are widespread across mouse models of spinocerebellar ataxias (SCAs) caused by CAG expansion mutations. The changes can be detected prior to disease onset, are repeat length dependent, and represent a novel transcriptomic driver of disease pathogenesis across CAG expansion SCAs.

## Introduction

Spinocerebellar ataxias (SCAs) are a genetically heterogeneous group of rare, dominantly inherited neurodegenerative disorders characterized by progressive ataxia. The characteristic symptoms of progressive loss of balance and coordination accompanied by slurred speech are driven by degeneration of the cerebellum, in particular, damage to cerebellar Purkinje neurons, although these are only mildly affected in some SCAs.^1-3^ Other interconnected regions of the nervous system, in particular the brainstem, are also involved and drive symptomology to a greater or lesser extent across the different SCAs. The most common SCAs are those caused by CAG repeat expansion mutations including SCA types 1, 2, 3, 6, 7, 12 and 17 in which the expanded CAG tracts are located in *ATXN1*, *ATXN2*, *ATXN3*, *CACAN1A*, *ATXN7*, *PPP2R2B* and *TBP*, respectively.^1,2^ For SCAs 1–3, 6, 7 and 17, the CAG repeat expansions are within the coding regions of the respective genes resulting in expression of polyglutamine expansion proteins. The expanded polyglutamine tracts alter protein conformation leading to disruption of normal function and protein aggregation.^3^

Given the shared trinucleotide repeat expansion mutation and the shared broad symptomology across CAG expansion SCAs, disruption to several cellular pathways has been implicated in disease pathogenesis across CAG expansion SCAs. Disruption to neuronal ion channels and alterations in membrane potential that impact Purkinje neuron firing and function have been reported across many CAG expansion SCAs.^2,4^ These findings are consistent with the many non-repeat expansion SCAs caused by mutations in ion channels or regulators of calcium ion homeostasis,^2,4^ such as *TRPC3* (SCA41) and *ITPR1* (SCA15/16 and SCA29).^4-7^ These changes in ion channel properties and membrane potential are not surprisingly associated with changes to synaptic signalling and function.^3^ Alterations in nuclear processes, such as DNA damage and repair and transcriptional dysregulation, have also been reported across multiple CAG expansion SCAs.^2,3^ Whilst attempts have been made to understand how CAG expansion mutations lead to disruption of these cellular pathways and contribute to neuronal dysfunction, degeneration and symptom onset, it remains a challenge to identify key transcriptomic drivers of disease pathogenesis in presymptomatic models of CAG expansion SCAs.

In several other trinucleotide repeat expansion diseases, alternative splicing dysregulation has been directly linked to key symptoms.^8^ Alternative splicing is a highly regulated mechanism that increases genetic diversity by processing RNA transcripts into distinct combinations of exons. Alternatively spliced exons are normally included or excluded to different extents in a spatio-temporal or developmental fashion governed by *trans*-acting spliceosomal factors and RNA binding proteins (RBPs) and *cis*-regulatory sequences that influence splice site selection.^8-10^ This process produces the mature mRNA transcripts and protein isoforms required by the cell type or developmental stage for normal functioning.^9^ In disease, these regulatory processes can go awry leading to dysregulation of alternative splicing resulting in different mRNA and protein isoform expression, which can have specific functional consequences and result in disease symptoms.^8,10^

The contribution of disease-associated dysregulation of alternative splicing to key symptoms is well characterized in the CTG repeat expansion disease, myotonic dystrophy type 1 (DM1).^8^ For example, missplicing of the *CLCN1* chloride channel mRNA leads to inclusion of exon 7A, which contains a premature stop codon. This triggers nonsense mediated decay of *CLCN1* transcripts, resulting in fewer CLCN1 ion channels and the hallmark symptom of DM1, myotonia.^11^ Likewise, missplicing of *SCN5* sodium channel mRNA, leads to inclusion of fetal exon 6A instead of adult exon 6B and results in cardiac arrythmia.^12^ These events are just two examples of the many alternative splicing events dysregulated in DM1, which have been used as biomarkers to distinguish severity of disease in DM1 patients and as target engagement biomarkers for preclinical therapeutic development studies.^13-15^ Together, these studies demonstrate that alternative splicing dysregulation can contribute directly to disease symptoms and can be used as biomarkers for monitoring disease progression in patients.

Studies in other CTG/CAG trinucleotide repeat expansion diseases have also implicated dysregulation of alternative splicing in disease pathogenesis. For example, in SCA8, the expression of the CUG expansion RNA has been shown to lead to splicing changes in a glutamate ionotropic receptor NMDA type subunit and a GABA transporter, which may contribute to the predicted loss of GABAergic inhibition in SCA8.^16^ Likewise, expression of expanded CUG RNAs was also linked to missplicing of a glutamate ionotropic receptor NMDA type subunit in SCA2.^17^ Several studies have also implicated alternative splicing dysregulation in genes linked to neurodegeneration and movement control in the CAG repeat expansion disorder, Huntington’s disease.^18-20^ Similarly, expression of CAG repeat expansions has been linked to missplicing of *CLCN1* and *SERCA1* (*ATP2A1*) in cell culture systems, with *SERCA1* being misspliced in SCA3 patient-derived fibroblasts.^21^

While these studies indicate the potential for alternative splicing dysregulation in several CAG repeat expansion SCAs, we do not currently understand the extent of splicing changes and their possible contribution to disease across these disorders. To address this gap, we performed a comprehensive analysis of alternative splicing across published RNA sequencing (RNA-Seq) data from CAG expansion SCA mouse models. In our analysis we compared mouse models expressing short and long CAG repeat expansions, investigated the interplay between differential gene expression and alternative splicing dysregulation and assessed the potential of alternative splicing as a target engagement biomarker for therapeutic studies in CAG SCAs.

## Materials and methods

### RNA sequencing data analysis

RNA-Seq datasets were acquired using the NCBI Sequence Read Archive (SRA) Explorer. FASTQ file quality was assessed using FastQC (version 0.11.9) and datasets with an average read depth of >35 million paired end reads were included in this study. FASTQ files with >20% adapter content were trimmed using fastp (version 0.23.2) and for samples comprised of more than one FASTQ file, files were merged to reach read depth threshold. FASTQ files were then aligned to the GRCm38/mm10 mouse reference genome using STAR (version 2.7.10a).^22^ RNA-Seq reads aligned to the *Atxn1* reference sequence were viewed using the Integrative Genomics Viewer (IGV 2.16.0)^23^ to identify mutations associated with the 154Q expansion allele. Differential gene expression was performed in RStudio (2022.12.0; R 4.2.2) using DESeq2 (version 3.16)^24^ and genes that passed a threshold of *P*adj < 0.05 and log2FC > |1.5| were considered significantly differentially expressed. Alternative splicing analysis was performed using rMATS (version 4.1.2)^25^ and events were considered significantly misspliced if the false-discovery rate (FDR) < 0.1 and ΔPSI (per cent spliced in) > |0.1|. The number of significant skipped exon events passing a threshold of *P* < 0.05 and ΔPSI > |0.1| is also reported for comparison with published datasets. All ΔPSI values are converted from a ratio to a percentage with the threshold adjusted accordingly: ΔPSI > |10%|. Exon numbers are referred to by previously published exon numbers for the same coordinates or based on counting the first exon in a gene as exon 1. Coordinates for reported exons are provided in Supplementary Table 1. Upset plots were generated using the ComplexHeatmap (2.10.0) R package.

### Gene ontology enrichment analysis

Gene ontology enrichment analysis was performed using Metascape (version v3.5.20230101)^26^ and the Database for Annotation, Visualization and Integrated Discovery (DAVID).^27,28^ Functional annotation data from DAVID was used to create enrichment maps in Cytoscape (version 3.9.1)^29^ with a node Q-value of 0.05 and an edge cut-off of 0.375 using the Edge weighted spring embedded layout based on overlap size.

### Allele-specific expression analysis for *Atxn1^2Q^* and *Atxn1^154Q^* using RNA-Seq data

Custom FASTA genomes were generated using the GRCm38/mm10 mouse reference genome edited to remove all sequences of *Atxn1* and *Atxn1-l* and add in minimal sequences for *Atxn1^2Q^* and *Atxn1^154Q^* alleles such that a 126 bp read or a 150 bp read would align with at least three sequence differences between the two alleles. Kallisto (version 0.48.0)^30^ was used to perform pseudo-alignment and quantify transcripts per million (TPM ± standard error). These custom genomes are available on request from the authors. Differential gene expression analysis was performed using sleuth (version 3.9)^31^ and data are reported as log2FC between SCA1 and SCA1 treatment or genetic cross ± standard error with FDR corrected *P*-values.

### Mouse studies

Mice used in this study were housed and treated in accordance with the NIH Guide for the Care and Use of Laboratory Animals and complied with the Albany Medical College Institutional Animal Care and Use Committee (IACUC) guidelines under approved animal care and use protocol numbers 20-04002 and 23-03002. *Atxn1^154Q/2Q^* mice were originally obtained from Jackson Laboratories (strain number 005601) and maintained according to established breeding protocols with genotyping performed by PCR, at weaning and retroactively, following existing protocols.^32^ Age and gender matched *Atxn1^154Q/2Q^* and wild-type (WT) littermates were anaesthetized at 6, 12 or 23 weeks of age using urethane in saline (1.2–1.5 g/kg) followed by a double thoracotomy and perfusion through the ascending aorta with 20 ml 1 × PBS. Whole cerebellum was removed and stored at −70°C.

### RT-PCR splicing analysis

RNA was extracted from cerebellum of wild-type and SCA1 *Atxn1^154Q/2Q^* KI mice using TRIzol (Ambion, Life Technologies) following the manufacturer’s instructions and a DNA digestion was performed using the TURBO DNA-free Kit (Invitrogen). RNA concentrations were measured using nanodrop and 500 ng total RNA was reverse transcribed using SuperScript IV reverse transcriptase (Invitrogen) with random hexamers (IDT). PCR for selected splicing events was performed using the Taq 2x master mix (NEB) with 2 μl cDNA under the following conditions: 95°C 30 s–32 cycles of 95°C 30 s, primer specific Tm 30 s, 68°C 30 s–68°C 5 min. Primer sequences, annealing temperatures and product sizes are as follows: Kcnma1 exon 23b Fw (5ʹ GGACAGATCATCACCCGACA 3ʹ), Rv (5ʹ ACAAGCAAAGGGCTGTGTGA 3ʹ), Tm 59.6°C, inclusion 260 bp, exclusion 180 bp; Anks1b exon 5 Fw (5ʹ ACAGACAGAGAATTCTACAAGCGA 3ʹ), Rv (5ʹ TTGAGGCTGTGGCTTCATTA 3ʹ), Tm 48.4°C, inclusion 610 bp, exclusion 540 bp; Trpc3 exon 9 primers are as previously reported.^33^ PCR products were resolved on a 5300 Fragment Analyzer (Agilent Technologies) using the 905 separation gel for Kcnma1 and the 910 separation gel for Trpc3 and Anks1b, following the manufacturer’s protocol. PCR products were resolved in technical triplicates on the fragment analyser and the average PSI is reported for each sample. PCR products were also resolved via agarose gel electrophoresis to confirm banding patterns seen via fragment analysis.

### Confirmation of *Atxn1^154Q^* sequence

To confirm the sequence immediately upstream of (CAG)_154_ in the *Atxn1^154Q/2Q^* repeats, a NaOH DNA extraction was performed on mouse ear punches and a PCR across the CAG repeat was performed using Atxn1-Rep-Fw (5ʹ CGTGTACCCTCCTCCTCAGT 3ʹ) and Atxn1-Rep-Rv (5ʹ ATTGCACAACCACCTGGGAT 3ʹ) under the following conditions: 1× Phire Hot Start II DNA Polymerase, 1× Phire Reaction Buffer (ThermoFisher), 1 M Betaine (Sigma), 0.4 mM dNTPs (NEB), 0.4 µM Atxn1-Rep-Fw, 0.4 µM Atxn1-Rep-Rv, 0.04 mM 7-deaza-dGTP (Roche); 98°C 7 mins–35 cycles of 98°C 30 s, 65.6°C 30 s, 72°C 75 s–72°C 5 mins. DNA was extracted from PCR bands using EZ-10 Spin Column DNA Gel Extraction Kit (Bio Basic) and sent for DNA sequencing using nested primers Atxn1-Seq-Fw (5ʹ CTTACGCGGGCTTTATCCCT 3ʹ) and Atxn1-Seq-Rv (5ʹ GCGGGATCATCGTCTGATGG 3ʹ).

### Allele-specific RT-qPCR for *Atxn1^2Q^* and *Atxn1^154Q^*

To confirm the specificity of our bioinformatic allele selective expression analyses for *Atxn1^2Q^* and *Atxn1^154Q^*, an allele specific RT-qPCR assay was performed. The following primer sets were used for detection of the expanded allele 154Q-Fw (5ʹ CTTACGCGGGCTTTATCCCT 3ʹ) and 154Q-Rv (5ʹ AGCCTTGTGTCCCGGCG 3ʹ) and the wild-type allele 2Q-Fw (5ʹ CAGGCACCAGGACATAAGGTTG 3ʹ) and 2Q-Rv (5ʹ CGTCTGATGGGGATGGAGGT 3ʹ), agarose gel electrophoresis was used to confirm primer specificity. Control reactions were performed for *Gapdh* Fw (5ʹ GCGAGACCCCACTAACATCA 3ʹ), Rv (5ʹ GGCGGAGATGATGACCCTTT 3ʹ) and total *Atxn1* Fw (5ʹ GAGAATCGAGGAGAGCCAC 3ʹ), Rv (5ʹ AGACTTCGACACTGACCT 3ʹ). All qPCR reactions were performed in technical quadruplicates using 1 μl cDNA with the PowerUP SYBR green master mix (ThermoFisher) according to the manufacturer’s instructions. To confirm specificity of primers, qPCR was performed on RT reactions in which the RT enzyme was replaced with H_2_O (RT−) under the same conditions. RT-qPCR data were analysed using the 2^−ΔΔCt^ method.^34^

### Data analysis

For qPCR and alternative splicing validation analyses, statistical analysis was performed using GraphPad Prism 9. Grubb’s test with an alpha of 0.05 was used to identify and remove any outliers for alternative splicing analyses of 6-week-old mice. Data are represented as mean ± standard error of the mean (SEM) and statistical analyses were performed using two-tailed Student’s unpaired *t*-test.

## Results

### RNA sequencing data from CAG expansion SCA mouse models show alternative splicing dysregulation

There are extensive RNA-Seq data available for different brain regions from mouse models of CAG expansion SCAs, which have previously been used to investigate differential gene expression.^35-49^ Here we sought to understand if changes in alternative splicing broadly represent a transcriptomic signature in CAG expansion SCAs. To do this, we performed alternative splicing analysis of publicly available RNA-Seq datasets. We performed a comprehensive search using the Gene Expression Omnibus (GEO) for RNA-Seq data from SCA mouse models using exact matches with search terms ‘Spinocerebellar ataxia type *N*’ and ‘SCA*N*’ where *N* = 1, 2, 3, 6, 7, 8, 12 or 17. All datasets identified using these search terms, that were from brain regions of CAG repeat expansion expressing mouse models, generated using RNA-Seq and available before August 2022 were included in our analysis. In addition, publicly available datasets reported with BioProject numbers but no GSE numbers were identified from publications. Through these searches, we identified 14 studies from across SCA types 1, 2, 3, 7 and 17 (Table 1).^35-49^

**Table 1:** RNA sequencing datasets used in this study

Disease dataset	Mouse model	Tissue type	Age	Avg. read depth	Pass thresh.	Skipped exon events (SCA versus WT)	
						*P* < 0.05; PSI > 10%	FDR < 0.1; PSI > 10%
SCA1,^35^ GSE122099	ATXN1[82Q] Tg	Cerebellum	5 w	87 537 211	Yes	650	93
	ATXN1[82Q] Tg	Cerebellum	12 w	92 485 001	Yes	759	136
	ATXN1[82Q] Tg	Inferior olive	5 w	99 996 263	Yes	610	55
	ATXN1[82Q] Tg	Inferior olive	12 w	97 497 000	Yes	609	69
	Atxn1 154Q/2Q	Cerebellum	5 w	104 290 286	Yes	684	87
	Atxn1 154Q/2Q	Cerebellum	12 w	115 188 043	Yes	797	154
	Atxn1 154Q/2Q	Inferior olive	5 w	87 880 555	Yes	780	147
	Atxn1 154Q/2Q	Inferior olive	12 w	99 154 063	Yes	757	101
SCA1,^43^ GSE114674	Atxn1 154Q/2Q, ASO treatment	Cerebellum	18 w	119 643 770	Yes	1009	362
	Atxn1 154Q/2Q, ASO treatment	Pons	18 w	82 569 603	Yes	1230	376
	Atxn1 154Q/2Q, ASO treatment	Medulla	18 w	82 934 663	Yes	1217	330
	Atxn1 154Q/2Q, ASO treatment	Pons	28 w	94 023 770	Yes	1157	462
	Atxn1 154Q/2Q, ASO treatment	Medulla	28 w	112 181 398	Yes	774	346
SCA1,^47^ GSE108256	Pcp2-ATXN1[82Q]	Cerebellum	12 w^a^	114 752 434	Yes	1016	523
SCA1,^38^ GSE75778^b^	ATXN1[30Q] Tg	Cerebellum	5 w	127 380 677	Yes	826	136
	ATXN1[82Q] Tg	Cerebellum	5 w	130 183 849	Yes	886	208
	**ATXN1[82Q] Tg**	**Cerebellum**	**12 w**	**10 182 436**	**No**	**NA**	**NA**
	**ATXN1[82Q] Tg**	**Cerebellum**	**28 w**	**9 168 448**	**No**	**NA**	**NA**
SCA1,^44^ GSE114815^c^	Pcp2-ATXN1[82Q]	Cerebellum	9 w	111 934 958	Yes	776	278
	ATXN1[82Q];CαM120A/M120A	Cerebellum	9 w	118 498 910	Yes	1450	994
SCA1,^42^ GSE163885^d^	Atxn1 154Q/2Q	Cerebellum	6 w	231 979 628	Yes	852	298
	Atxn1 154Q[S776A]/2Q	Cerebellum	6 w	230 593 782	Yes	772	231
SCA2,^45^ PRJEB24319	**ATXN2 Q127 Tg**	**Cerebellum**	**1 day**	**22 095 459**	**No**	**NA**	**NA**
	**ATXN2 Q127 Tg**	**Cerebellum**	**3 w**	**23 312 280**	**No**	**NA**	**NA**
	**ATXN2 Q127 Tg**	**Cerebellum**	**6 w**	**21 850 856**	**No**	**NA**	**NA**
SCA3,^49^ GSE107958	MJD84.2	Cerebellum	17.5 mo	183 848 391	Yes	421	75
	MJD84.2	Cortex	17.5 mo	175 864 178	Yes	460	84
	MJD84.2	Striatum	17.5 mo	193 332 930	Yes	634	167
	MJD84.2	Brainstem	17.5 mo	68 227 029	Yes	463	127
SCA3,^46^ GSE117605^e^	YAC15Q hemi	Pons	22–24 w	51 185 663	Yes	583	101
	YAC84Q hemi	Pons	22–24 w	56 641 310	Yes	645	157
	**KI-het**	**Pons**	**22–24 w**	**4 714 289**	**No**	**NA**	**NA**
	KI-hom	Pons	22–24 w	59 753 664	Yes	700	165
SCA3,^37^ GSE145613	304/304Q	Cerebellum	2 mo	47 636 024	Yes	970	547
	304/304Q	Cerebellum	12 mo	36 060 638	Yes	1154	698
SCA3,^39^ GSE178367^f^	YAC15Q (no WT)	Cerebellum	18 mo	104 531 443	Yes	NA	NA
	YAC84Q, IGF1 treatment	Cerebellum	18 mo	58 606 848	Yes	1249	445
SCA7,^41^ GSE138527^g^	140Q/5Q	Cerebellum	40 w	43 319 128	Yes	885	248
SCA7,^48^ GSE139090	**SCA7 92Q**	**Cerebellum**	**12 w**	**33 206 832**	**No**	**NA**	**NA**
	**SCA7 92Q**	**Cerebellum**	**29 w**	**34 817 416**	**No**	**NA**	**NA**
SCA17,^40^ GSE145067	**TBP-105Q**	**Cerebellum**	**3 mo**	**11 548 250**	**No**	**NA**	**NA**
	**TBP-105Q**	**Striatum**	**3 mo**	**10 299 866**	**No**	**NA**	**NA**
	**TBP-105Q**	**Prefrontal cortex**	**3 mo**	**9 952 160**	**No**	**NA**	**NA**

Datasets in bold do not pass read depth threshold for alternative splicing analysis. An expanded version of Table 1 including replicate number, library selection method, sequencing method and read length for each dataset can be found in the Supplementary material.

ASO = antisense oligonucleotide; FDR = false discovery rate; KI = knock-in; mo = months; NA = not applicable - datasets did not pass read depth threshold and so alternative splicing analysis was not performed; PSI = per cent spliced in; SCA = spinocerebellar ataxia; w = weeks; WT = wild-type.

^a^Publication states 12 weeks, GSE states 12 months.

^b^FastQ files merged to reach read depth threshold.

^c^Technical replicates combined into one FastQ file.

^d^FastQ files contain ∼15–20% adapter content but had sufficient read depth and therefore untrimmed.

^e^FastQ files trimmed for high adapter content to increase quality and merged to reach read depth threshold.

^f^Dataset GSE178367 does not contain wild-type mice, skipped exon numbers are reported for YAC84Q versus YAC15Q. Library selection and mouse model terminology matches dataset publications.

^g^Dataset GSE138527 is the only single-stranded dataset used in the analysis and was included due to containing >40 million reads and resulted in robust detection of alternatively spliced events allowing SCA7 to be included in this analysis.

To quantify a specific alternative splicing event using RNA-Seq data, multiple reads must map across the exon-exon junctions for both the inclusion and exclusion products. This contrasts with differential gene expression where reads mapping anywhere in a specific transcript can be used for quantification of that transcript’s expression. The sensitivity of detecting alternative splicing events therefore depends on the read depth of the RNA-Seq data, with read length also influencing the accuracy of analysis.^25,50-52^ The 14 studies identified have variable read depths and read lengths (Table 1). Based on a conservative estimate from our previous alternative splicing studies alongside those of others,^13,25,51,53^ to maximize sensitivity of detecting alternative splicing events and validity of cross-dataset comparisons, we set a minimum threshold of 35 million paired-end reads. Of the identified studies, 11 passed this threshold including data from SCA1, SCA3 and SCA7 mouse models. Together these 11 studies include 29 control versus disease model comparisons, which we will refer to as datasets (Table 1). All datasets analysed were generated using polyA selection during library preparation and, excluding the single SCA7 dataset, were sequenced using paired-end reads (Table 1).^35-39,41-44,46,47,49^ Alternative splicing analysis was performed for age-matched wild-type versus SCA mice for each dataset and two thresholds were used to define significance based on previous Huntington’s disease and myotonic dystrophy alternative splicing studies.^13,18-20,53^ Across all datasets analysed, changes in alternative splicing, which may represent missplicing or dysregulation of alternative splicing, were identified (Table 1 and Supplementary Tables 2–5). These data demonstrate the presence of a shared transcriptomic hallmark across CAG expansion SCA mouse models.

### Alternative splicing dysregulation is a cerebellar transcriptomic hallmark of CAG expansion SCA mouse models

**Figure 1:**
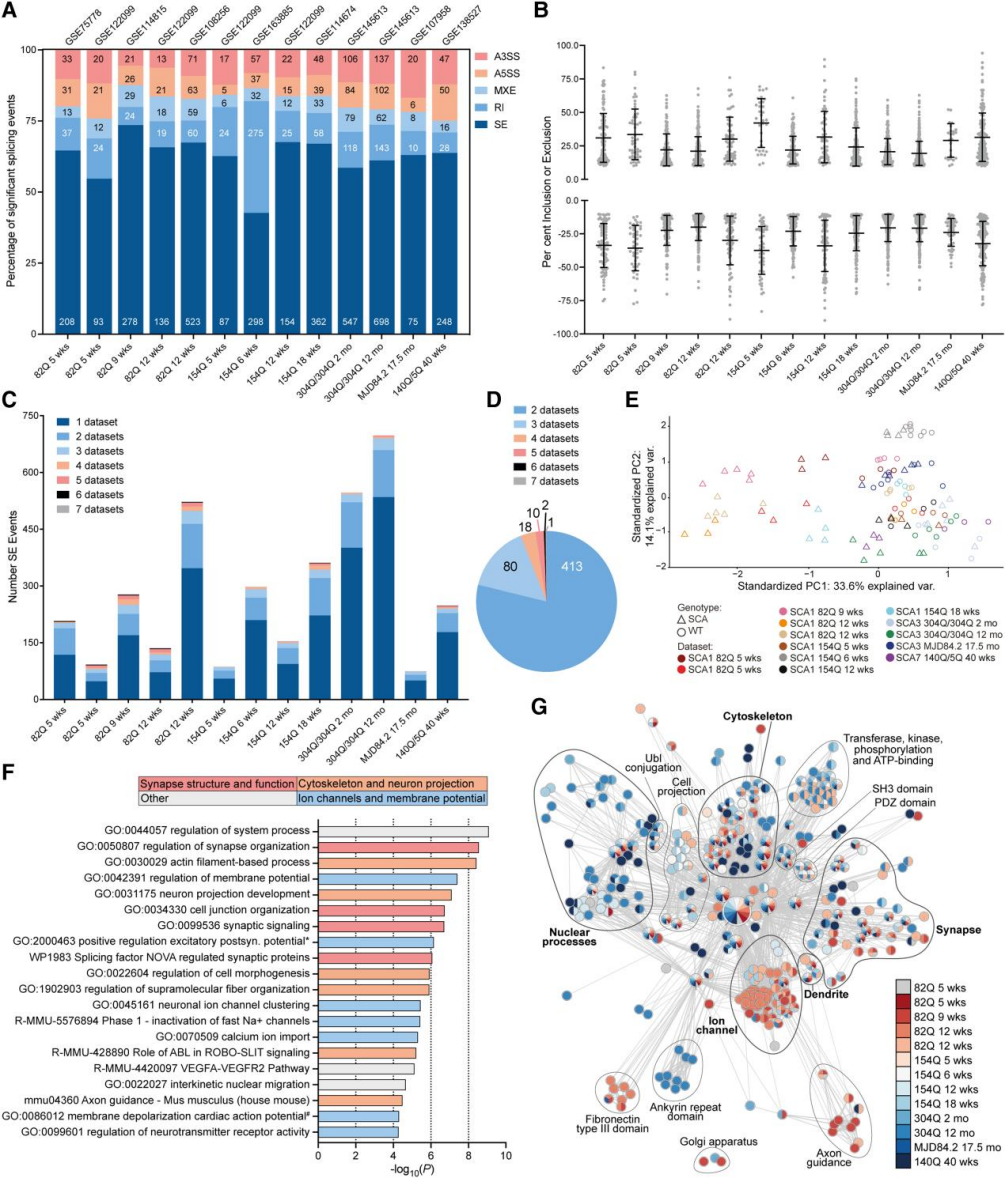
**Widespread dysregulation of alternative splicing in CAG expansion SCA mouse model cerebellum.** (**A**) Percentage of significantly misspliced skipped exon (SE), retained intron (RI), mutually exclusive exons (MXE), alternative 5’ splice site (A5SS) and alternative 3’ splice site (A3SS) events as a proportion of total splicing events in spinocerebellar ataxia (SCA) versus wild-type (WT) mice, number of each event shown on bar, FDR < 0.1, ΔPSI > 10%. (**B**) Percentage of exon inclusion (positive) or exclusion (negative) for significantly alternatively spliced skipped exon events per dataset, FDR < 0.1, ΔPSI > 10%, mean ± standard deviation (SD). (**C**) Number of skipped exon events per dataset with the proportion of events dysregulated in two to seven datasets shown, FDR < 0.1, ΔPSI > 10%. (**B** and **C**) Datasets are in the same order as **A**. (**D**) Total number of skipped exon events dysregulated in more than one dataset, FDR < 0.1, ΔPSI > 10%. (**E**) Principal component analysis of the 31 skipped exon events dysregulated in four or more datasets. (**F**) Enrichment of summary gene ontology terms identified using Metascape for the 524 skipped exon events dysregulated in two or more datasets; broad functional categories of terms are indicated by bar colour; see Supplementary Fig. 2 and Supplementary Table 8 for member gene ontology terms. Full gene ontology terms: *GO:2000463 positive regulation of excitatory postsynaptic potential; ^#^GO:0086012 membrane depolarization during cardiac muscle cell action potential. (**G**) Functional classification analysis of significantly misspliced skipped exon events, *P* < 0.05, ΔPSI > 10%; see Supplementary Fig. 4 for detailed annotation. FDR = false discovery rate; PSI = per cent spliced in.

To understand whether dysregulation of alternative splicing could contribute to cerebellar phenotypes in CAG expansion SCA mouse models, we performed a comprehensive analysis of the 13 cerebellar datasets identified (Table 1). Of the significant misspliced events identified with a ΔPSI > 10% and FDR < 0.1, skipped exon (SE) or cassette exon events were the most frequently dysregulated across all datasets. In 12 of the 13 datasets, skipped exon events accounted for more than 50% of the total misspliced events (Fig. 1A). Cassette exon or skipped exon events are alternative splicing events where an intervening exon is included or excluded between two other exons to form two different mature mRNAs and result in distinct protein isoforms.^8,10,25,54^ Alternative splicing of mutually exclusive exons (MXE), retained introns (RI), alternative 5ʹ splice site (A5SS) and alternative 3ʹ splice site (A3SS) were also detected in all datasets (Fig. 1A and Supplementary Table 6). As skipped exon events were the most frequently dysregulated, we sought to understand the extent and effect of their dysregulation.

Although differences in read depth, read length, library preparation and sequencing methods hinder comparisons of splicing event numbers between different studies,^25,50-52,54^ for datasets within a study there was an increase in skipped exon events throughout disease progression. For example, *ATXN1*-82Q cerebellum had 93 skipped exon events at 5 weeks and 136 at 12 weeks. Likewise, in the *Atxn1^154Q/2Q^* mouse model there was an increase from 87 skipped exon events at 5 weeks to 154 at 12 weeks of age in dataset GSE122099. This was also seen in the 304Q/304Q SCA3 mouse model with 547 skipped exon events at 2 months of age and 648 at 12 months (Fig. 1A; FDR < 0.1, ΔPSI > 10%). All datasets showed dysregulation of both inclusion events (an exon is included more frequently in SCA than wild-type mice) and exclusion events (an exon is excluded more frequently in SCA than wild-type mice) and across the datasets there was no predominance for dysregulation of inclusion versus exclusion events. The mean ΔPSI for inclusion events ranged from 19.5% to 42.1% with the maximum ΔPSI per dataset ranging from 52.1% to 94.2%. For exclusion events, the mean ΔPSI ranged from 20% to 37.4% with the maximum ΔPSI for exon exclusion ranging from 49.7% to 89.9% across datasets (Fig. 1B).

We next sought to understand the extent to which skipped exon missplicing was shared between the datasets. Each dataset contained skipped exon events that were misspliced in only that dataset, as well as events that were misspliced in two, three or more datasets (Fig 1C, Supplementary Fig. 1A and Supplementary Table 4). Across all datasets, a total of 2945 skipped exon events (ΔPSI > 10%, FDR < 0.1) were detected with 524 being shared between two or more datasets and one event, *Trpc3* exon 9, misspliced in seven datasets (Fig 1D and Supplementary Fig. 1A). By performing a principal component analysis (PCA) of skipped exon events shared between four or more datasets, we saw a global trend of SCA samples (triangles) clustered to the left and wild-type samples (circles) to the right. This distribution occurred as a global trend across all datasets and within each dataset, except for the 2 month and 17.5-month SCA3 datasets, which showed limited overlap with the SCA1 and SCA7 datasets (Fig 1E, Supplementary Fig. 1A and Supplementary Table 4).

We next wanted to identify if the cerebellar missplicing occurred in disease-relevant pathways previously implicated in CAG expansion SCAs. We performed gene ontology enrichment analysis of the genes with skipped exon events dysregulated in two or more datasets (524 skipped exon events) using Metascape.^26^ The four most enriched gene ontology summary terms were regulation of synapse organization, actin filament-based processes, regulation of membrane potential, and regulation of system process. Of the top 20 enriched gene ontology terms, 17 clustered into three broad categories based on the top four terms: synapse structure and function (e.g. splicing factor NOVA regulated synaptic proteins) (Supplementary Table 7), cytoskeleton and neuron projection (e.g. neuron projection development), and ion channels and membrane potential (e.g. calcium ion import) (Fig. 1F, Supplementary Fig. 2 and Supplementary Table 8). These pathways have all previously been shown to be affected or implicated in SCA disease.^1,2^ To confirm the enrichment of these pathways at the level of individual datasets, we performed gene ontology enrichment analysis using Metascape^26^ (Supplementary Fig. 3A) and functional annotation clustering using DAVID^27-29^ (Fig. 1G and Supplementary Fig. 4) for significant skipped exon events (*P* < 0.05, ΔPSI > 10%). Both analyses confirmed that skipped exon events occurred in genes implicated in synapse structure and function, the cytoskeleton and ion channels. Interestingly, both of these broader analyses revealed enrichment of nuclear process-associated terms such as DNA damage and repair, and zinc-fingers and transcriptional regulation. Terms specifically enriched in single datasets such as Ankyrin repeat domain in the 12-month 304Q/304Q SCA3 dataset were also identified in this analysis, but these were less common than the shared terms (Fig. 1G, Supplementary Figs. 3A, 4 and Supplementary Table 8). Together these data demonstrate that dysregulation of alternative splicing is a shared transcriptomic hallmark of cerebellum from CAG expansion SCA mouse models and that alternative splicing occurs in genes involved in cellular pathways previously implicated in SCAs.

### Alternative splicing dysregulation occurs across brainstem and cortical regions of CAG expansion SCA mouse models

**Figure 2:**
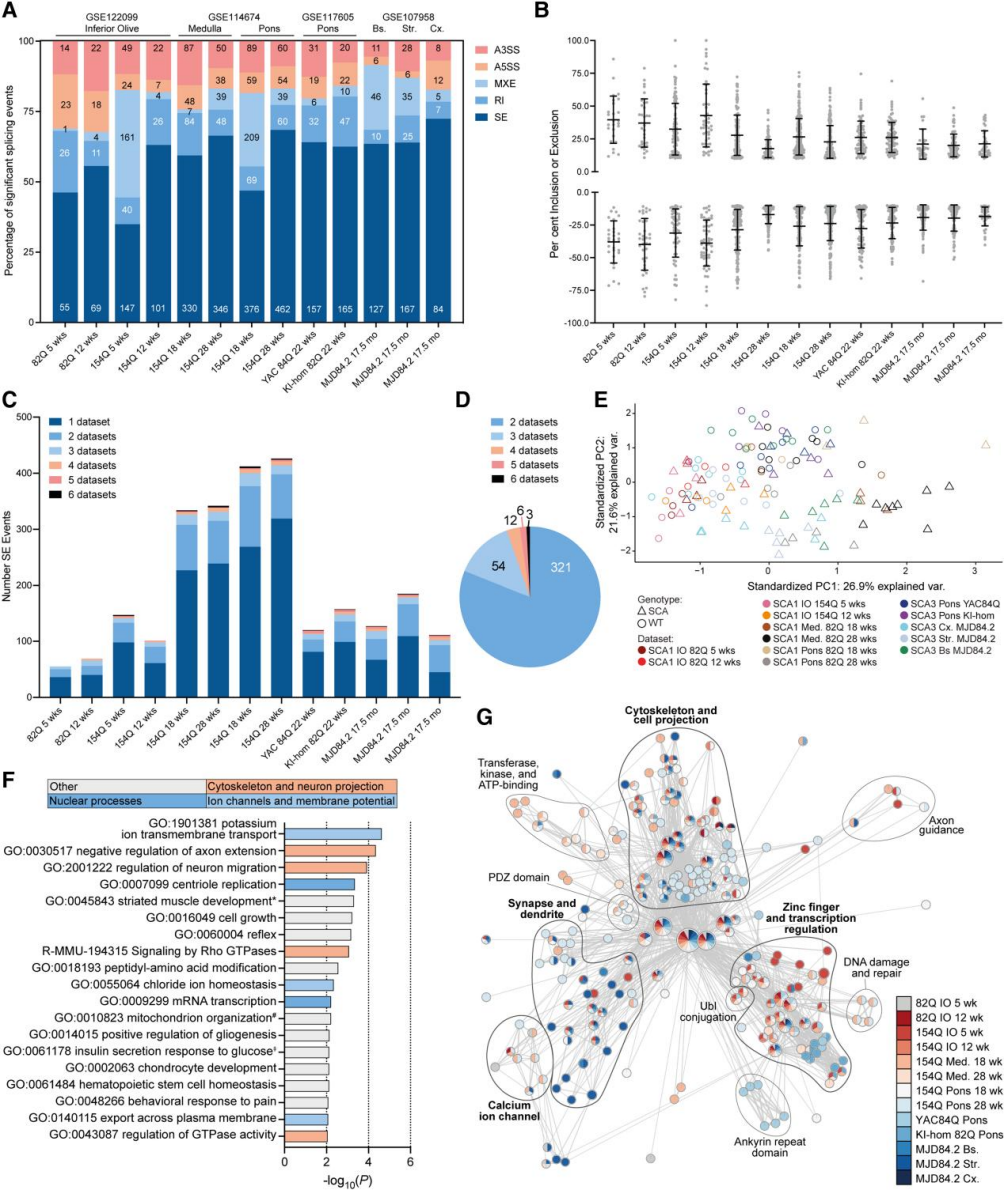
**Alternative splicing is dysregulated across affected brain regions in CAG expansion SCA mouse models.** (**A**) Percentage of significantly misspliced skipped exon (SE), retained intron (RI), mutually exclusive exons (MXE), alternative 5’ splice site (A5SS) and alternative 3ʹ splice site (A3SS) events as a proportion of total splicing events in spinocerebellar ataxia (SCA) versus wild-type (WT) mice, number of each event shown on bar, FDR < 0.1, ΔPSI > 10%. (**B**) Percentage of exon inclusion (positive) or exclusion (negative) for significantly alternatively spliced skipped exon events per dataset, FDR < 0.1, ΔPSI > 10%, mean ± standard deviation (SD). (**C**) Number of skipped exon events per dataset with the proportion of events dysregulated in two to seven datasets shown, FDR < 0.1, ΔPSI > 10%. (**B** and **C**) Datasets are in the same order as **A**. (**D**) Total number of skipped exon events dysregulated in more than one dataset, FDR < 0.1, ΔPSI > 10%. (**E**) Principal component analysis of the nine skipped exon events dysregulated in five or six datasets. (**F**) Enrichment of summary gene ontology terms identified using Metascape for the 396 skipped exon events dysregulated in two or more datasets; broad functional categories of terms are indicated by bar colour; see Supplementary Fig. 5 and Supplementary Table 8 for member gene ontology terms. Full gene ontology terms: *GO:0045843 negative regulation of striated muscle tissue development; ^#^GO:0010823 negative regulation of mitochondrion organization; ^‡^GO:0061178 regulation of insulin secretion involved in cellular response to glucose stimulus. (**G**) Functional classification analysis of significantly misspliced skipped exon events, *P* < 0.05, ΔPSI > 10%; see Supplementary Fig. 6 for detailed annotation. FDR = false discovery rate; PSI = per cent spliced in.

We next investigated whether alternative splicing occurred in other affected brain regions of SCA mouse models. Similar to the cerebellar analyses, skipped exon events accounted for >50% of all misspliced events (ΔPSI > 10%, FDR < 0.1) in SCA mice versus wild-type mice for 10 of the 13 cortical and brainstem region datasets (Fig. 2A and Supplementary Table 6). Importantly, few events were detected in the inferior olives from *ATXN1*-82Q mice (Fig. 2A), which only express the CAG expansion transgene in cerebellar Purkinje neurons.^35,55^ Across these datasets, dysregulation of both inclusion and exclusion events was observed with the mean ΔPSI for inclusion events ranging from 17.6% to 42.8% and for exclusion events from 17.1% to 39.8%. The ranges for maximum ΔPSI values were 43.1% to 100% for inclusion events and 40.3% to 86.6% for exclusion events (Fig. 2B). Again, we identified more dysregulated skipped exon events unique to each dataset than shared, but all datasets except the *ATXN1*-82Q inferior olive datasets included events that were misspliced in five or six datasets (Fig. 2C and Supplementary Fig. 1B). Overall, 2039 skipped exon events were identified with 396 dysregulated in two or more datasets (Fig. 2D, Supplementary Fig. 1B and Supplementary Table 5). Using events that were shared between five or six datasets, we performed PCA and saw a global separation of the wild-type (circles, upper left) and SCA (triangles, lower right) samples as a global trend and within each dataset (Fig. 2E).

To understand possible implications of these misspliced events, gene ontology enrichment analysis of the genes with skipped exon events dysregulated in two or more datasets (396 skipped exon events) was performed using Metascape.^26^ The four most enriched gene ontology summary terms were potassium ion transmembrane transport, negative regulation of axon extension, regulation of neuron migration, and centriole replication (Fig. 2F, Supplementary Fig. 5 and Supplementary Table 8). Gene ontology enrichment analysis of significant skipped exon events (*P* < 0.05, ΔPSI > 10%) at the level of individual datasets, using both Metascape^26^ and functional annotation clustering,^27-29^ confirmed that skipped exon events occurred in genes implicated in the cytoskeleton and neuron projections, ion channels and membrane potential, and nuclear processes, including regulation of transcription and DNA damage and repair. These broader analyses also identified enrichment of terms related to synapse structure and function, such as synaptic signalling, regulation of vesicle-mediated transport and postsynaptic density (Fig. 2G, Supplementary Figs. 3B, 6 and Supplementary Table 8). Together these analyses demonstrate that alternative splicing dysregulation also occurs across affected brain regions other than cerebellum from SCA1 and SCA3 mouse models and is implicated in the dysregulation of disease relevant pathways.

### Mouse models with short versus pathogenic CAG repeats have distinct splicing profiles

**Figure 3:**
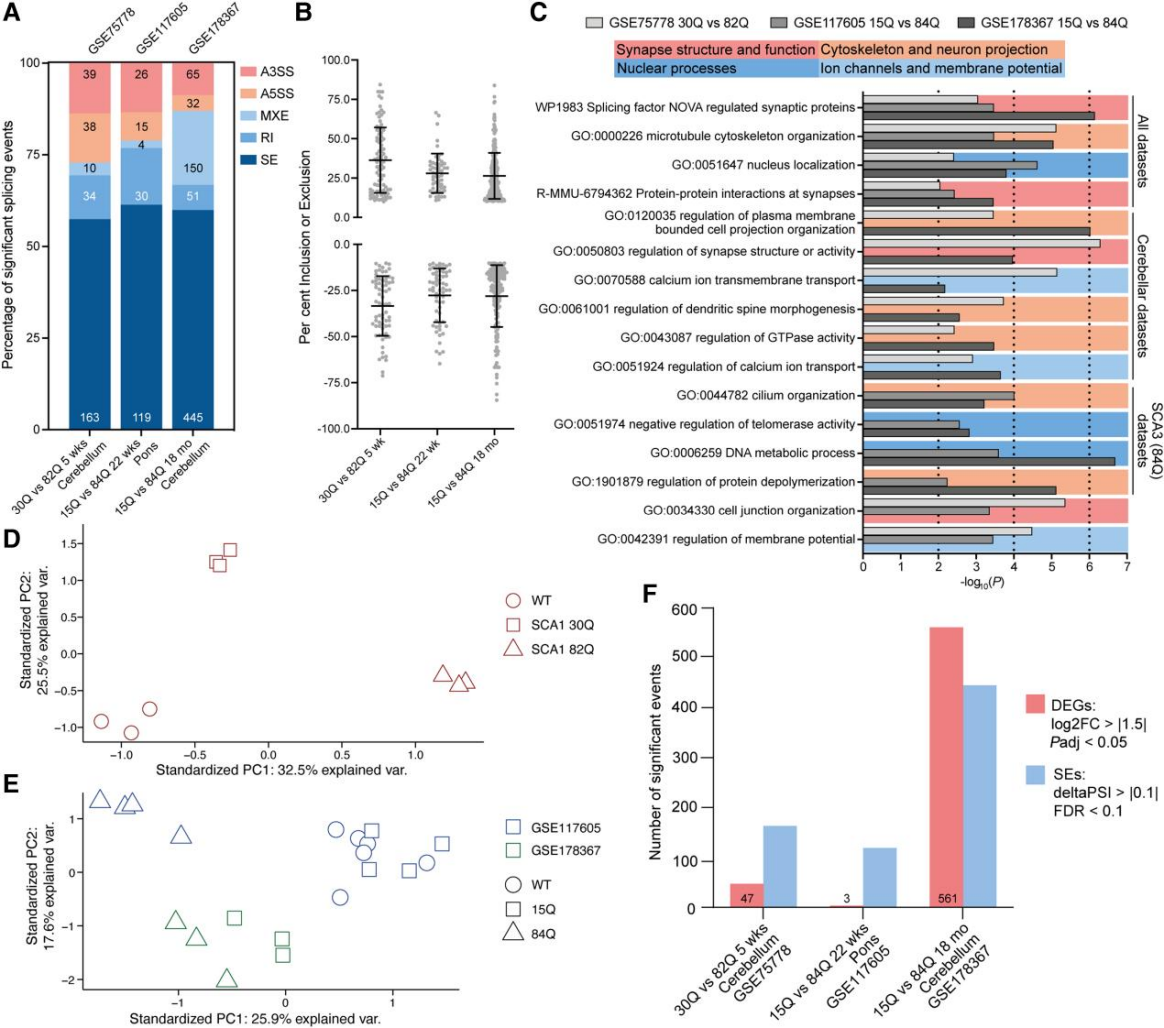
**Short and pathogenic repeat length mouse models show distinct splicing profiles.** (**A**) Percentage of significantly misspliced skipped exon (SE), retained intron (RI), mutually exclusive exons (MXE), alternative 5ʹ splice site (A5SS) and alternative 3ʹ splice site (A3SS) events as a proportion of total splicing events in spinocerebellar ataxia (SCA) versus short repeat mice, number of each event shown on bar, FDR < 0.1, ΔPSI > 10%. (**B**) Percentage of exon inclusion (positive) or exclusion (negative) for significantly alternatively spliced skipped exon events per dataset, FDR < 0.1, ΔPSI > 10%, mean ± standard deviation (SD). (**C**) Enrichment of gene ontology terms for significantly misspliced skipped exon events between short and pathogenic repeat length mice across the three datasets; broad functional categories of terms are indicated by background colours. (**D**) Principal component analysis of significantly dysregulated skipped exon events in the SCA1 dataset GSE75778. (**E**) Principal component analysis of skipped exon events significantly dysregulated in two or more pairwise comparisons from the SCA3 datasets GSE117605 and GSE178367. (**F**) Number of differentially expressed genes (DEGs) and skipped exon events in short repeat versus pathogenic repeat length mice; numbers of DEGs are shown on bars; DEGs: log2FC>|1.5|, *P*adj < 0.05; SE: FDR < 0.1, ΔPSI > 10%. FDR = false discovery rate; PSI = per cent spliced in.

To understand how alternative splicing dysregulation is connected with CAG repeat expansions, we assessed if missplicing was repeat-tract length-dependent. Of the 11 studies identified that passed our read depth threshold, three included data from mouse models with short, non-pathogenic CAG tracts generated using the same strategy as the CAG expansion SCA mouse model in each study.^38,39,46^ We performed alternative splicing analysis of these short repeat mice versus the age matched expansion SCA mice and, where available, age-matched wild-type mice (Table 1). We noted an increase in the number of skipped exon events (FDR > 0.1, ΔPSI > 10%) in the expansion mice compared to the short repeat mice when analysed against wild-type mice for both SCA1 (30Q versus WT: 136 skipped exon; 82Q versus WT: 208 skipped exon) and SCA3 (15Q versus WT: 76 skipped exon; 84Q versus WT: 157 skipped exon) datasets (Table 1). Analysis of the short repeat versus pathogenic repeat length mice revealed alternative splicing changes for all event types, with skipped exon events accounting for >50% of misspliced events across all three datasets (Fig. 3A). The maximum ΔPSI for inclusion values across these comparisons ranged from 66.5% to 84.4% and for exclusion values from 64.7% to 84.5%. The mean ΔPSI for inclusion events ranged from 26.4% to 36.3% and for exclusion values from 27.7% to 33.4% (Fig. 3B and Supplementary Table 9). These data demonstrate that the magnitude of splicing changes for skipped exon events from short repeat versus SCA mouse models are comparable to the magnitude of splicing changes seen for wild-type versus SCA comparisons (Figs 1B and 2B).

Gene ontology enrichment analysis^26^ of skipped exon events significantly alternatively spliced between short and pathogenic repeat length mice (*P* < 0.05, ΔPSI > 10%) demonstrated that misspliced genes were in disease-related processes. Amongst the enriched terms were synapse structure and function (e.g. splicing factor NOVA regulated synaptic proteins) (Supplementary Table 7), the cytoskeleton (e.g. microtubule cytoskeleton organization), nuclear processes (e.g. nuclear localization) and ion channels (e.g. calcium ion transmembrane transport). Interestingly, nuclear process-associated terms were more commonly enriched across the SCA3 datasets, while ion channel-associated terms were more commonly enriched across the cerebellar datasets (Fig. 3C and Supplementary Table 8).

To understand the relative splicing profiles of short repeat and SCA mouse models, we performed PCAs. For the SCA1 dataset, a PCA of significantly misspliced skipped exon events (FDR < 0.1, ΔPSI > 10%) in one or more of the pairwise alternative splicing analyses (30Q versus WT, 82Q versus WT and 30Q versus 82Q) was performed (Fig. 3D). For the SCA3 datasets, the PCA was performed based on skipped exon events significantly misspliced in two or more pairwise comparisons (Fig. 3E). In both cases, clear separation between short and pathogenic repeat samples was observed. For the SCA1 dataset, PC1 explained 32.5% of the variance between samples, with separation of the 82Q mice versus the 30Q and wild-type mice occurring in this direction. The 30Q and wild-type mice did show distinct clustering along PC2, which explained less variance in the data (25.5%) (Fig. 3D) and could be driven by effects based on transgene insertion sites and transgene copy number.^55^ For the SCA3 data, the 15Q and wild-type pons samples clustered together and were distinct from the 84Q pons samples (blue, dataset GSE117605). The 15Q and 84Q cerebellar samples (green, dataset GSE178367) likewise showed distinct clustering with the 15Q samples clustering nearer the wild-type and 15Q pons samples (Fig. 3E).

Finally, we assessed the contribution of alternative splicing dysregulation to repeat length-dependent transcriptomic signatures. We performed transcriptomic analysis between the short and pathogenic repeat length mice. For the presymptomatic (5 week) SCA1 82Q versus 30Q cerebellum, we saw over three times as many misspliced skipped exon events (*n* = 163) as differentially expressed genes (*n* = 47). Similarly, in the early symptomatic (22 week) SCA3 84Q versus 15Q pons, only three genes were differentially expressed but there were 119 misspliced skipped exon events. However, the symptomatic (17.5 month) SCA3 84Q versus 15Q cerebellum showed more differentially expressed genes (*n* = 561) than misspliced skipped exon events (*n* = 445) (Fig. 3F). Together these data demonstrate that mouse models of short CAG repeats and CAG repeats in the pathogenic expansion range for SCAs have distinct splicing profiles affecting pathways relevant to SCA disease, and that at early disease time points alternative splicing may contribute more to global transcriptomic dysregulation than differential gene expression.

### Presymptomatic SCA mouse models show disease relevant dysregulation of skipped exon events

**Figure 4:**
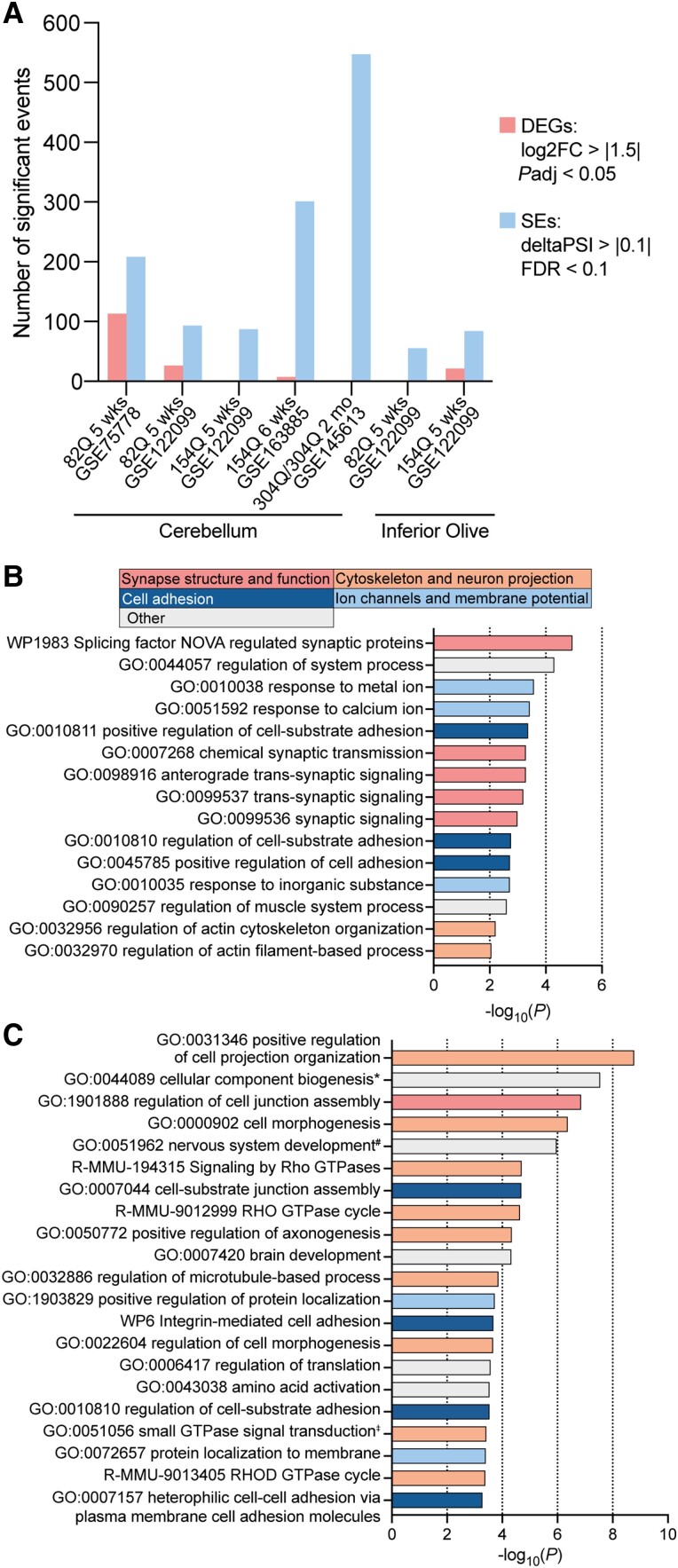
**Presymptomatic splicing changes occur in disease relevant pathways.** (**A**) Number of differentially expressed genes (DEGs) and skipped exon (SE) events in presymptomatic SCA1 or SCA3 versus wild-type (WT) mice; DEGs: log2FC>|1.5|, *P*adj < 0.05; SE: FDR < 0.1, ΔPSI > 10%. (**B**) Gene ontology enrichment analysis of the 28 skipped exon events significantly dysregulated across two or more SCA1 presymptomatic cerebellar datasets, FDR < 0.1, ΔPSI > 10%. (**C**) Gene ontology enrichment analysis of the 304Q/304Q presymptomatic SCA3 dataset, FDR < 0.1, ΔPSI > 10%. Full gene ontology terms: *GO:0044089 positive regulation of cellular component biogenesis; ^#^GO:0051962 positive regulation of nervous system development; ^‡^GO:0051056 regulation of small GTPase mediated signal transduction. (**B** and **C**) Broad functional categories of terms are indicated by bar colour.

We observed an increase in misregulation of skipped exon events across disease progression (Fig. 1A) and greater dysregulation of alternative splicing than differential gene expression in pre- and early symptomatic CAG expansion versus short repeat mice (Fig. 3F). Based on these observations, we next sought to understand if dysregulation of alternative splicing could represent a pathogenic mechanism for early neuronal dysfunction in CAG expansion SCAs. We performed transcriptomic analysis for all presymptomatic datasets, which included six datasets from 5- and 6-week-old *ATXN1*-82Q and *Atxn1^154Q/2Q^* SCA1 mice and one dataset from 2-month-old 304Q/304Q SCA3 mice. In all datasets, using stringent thresholds for both differential gene expression (*P*adj < 0.05, log2FC > |1.5|) and alternative splicing (FDR < 0.1, ΔPSI > 10%), we identified more skipped exon events than differentially expressed genes (Fig. 4A and Supplementary Table 10). This effect was especially pronounced in the SCA3 dataset [one differentially expressed gene (DEG); 547 skipped exons] and the 6-week-old *Atxn1^154Q/2Q^* dataset (seven DEGs; 298 skipped exons; Fig. 4A), the latter of which has roughly double the read depth and longer read length than the other presymptomatic SCA1 datasets (Table 1).

Between the four SCA1 presymptomatic cerebellar datasets there were 28 skipped exon events alternatively spliced (FDR < 0.1, ΔPSI > 10%) in two or more of the datasets. Gene ontology enrichment analysis^26^ of these events identified that the genes function in: synapse structure and function (e.g. splicing factor NOVA regulated synaptic proteins) (Supplementary Table 7), the cytoskeleton (e.g. regulation of actin cytoskeleton organisation), ion channels (e.g. response to calcium ion) and cell adhesion (e.g. positive regulation of cell substrate adhesion). Of the 15 enriched gene ontology terms, 13 clustered into these four functional categories (Fig. 4B, Supplementary Table 8 and Supplementary Figs 3A and 4 for individual dataset gene ontology analyses). As cell adhesion has not previously been identified as an enriched functional category in our analyses, we performed gene ontology analysis using Metascape^26^ for the presymptomatic SCA3 cerebellar dataset (FDR < 0.1, ΔPSI > 10%). Interestingly, we also identified cell adhesion-associated terms such as cell-substrate junction assembly and integrin mediated cell adhesion, suggesting that cell adhesion may be impaired during early stages of disease. We again identified terms related to the cytoskeleton (e.g. regulation of microtubule-based processes) and ion channels and membrane potential (e.g. protein localization to membrane), but we identified very few terms associated with synapse structure and function (Fig. 4C and Supplementary Table 8). These analyses demonstrate that presymptomatic transcriptomic dysregulation in both SCA1 and SCA3 mice is characterized by disease-relevant defects in alternative splicing and not by widespread changes in differential gene expression.

### Dysregulation of key alternative splicing events occurs across SCA1, SCA3 and SCA7 mouse models

**Figure 5:**
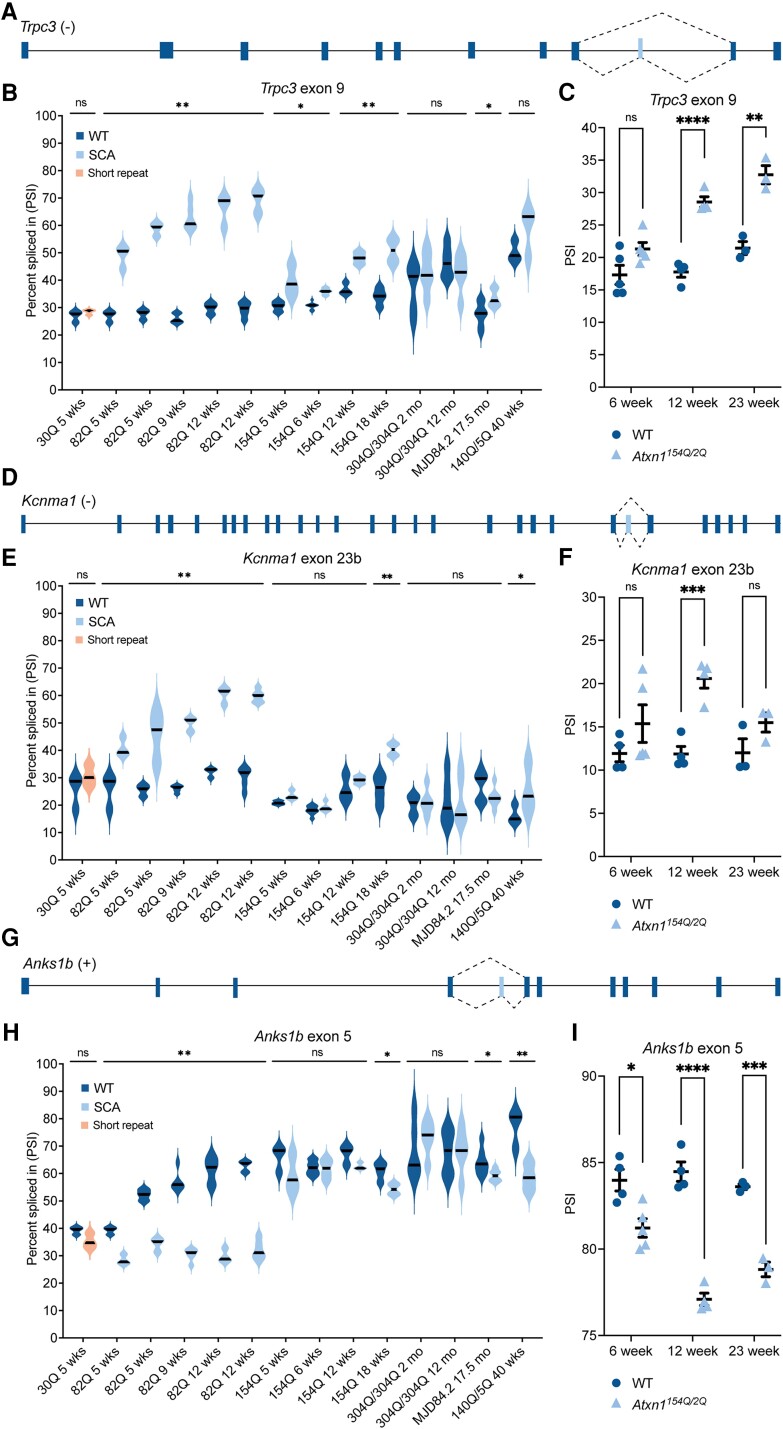
***Trpc3, Kcnma1* and *Anks1b* are significantly misspliced across SCA1, 3 and 7 mouse models.** (**A**, **D** and **G**) Schematics of each gene indicating exon positions and the significantly misspliced skipped exon identified in this study; the skipped exons are referred to as previously published exon numbers for these events or based on counting the first exon as exon 1. (**B**, **E** and **H**) Violin plots of RNA-Seq data showing increased inclusion of *Trpc3* exon 9 and *Kcnma1* exon 23b and increased exclusion of *Anks1b* exon 5, respectively, across SCA1, SCA3 and SCA7 mouse models; datasets shown in the same order as Fig. 1A. ns = not significant, *P < 0.05, **FDR < 0.1, line indicates median. (**C**, **F** and **G**) RT-PCR analysis of *Trpc3* exon 9, *Kcnma1* exon 23b and *Anks1b* exon 5, respectively, in cerebellum from presymptomatic (6 week, *n* = 4–5), early symptomatic (12 week, *n* = 4) and mild symptomatic (23 week, *n* = 3) *Atxn1^154Q/2Q^* SCA1 mouse models; ns = not significant, *P < 0.05, **P < 0.01, *** *P* < 0.001, **** *P* < 0.0001, mean ± standard error of the mean (SEM); 6 week *Trpc3* exon 9 *P* = 0.0568.

To understand the possible contribution of alternative splicing to neuronal dysfunction and disease in CAG expansion SCAs, we tracked three specific alternative splicing events dysregulated in presymptomatic mice across all datasets. The three skipped exon events that were dysregulated in six or seven cerebellar datasets (Fig. 1D) were all significantly dysregulated in two of the four presymptomatic SCA1 cerebellar datasets (FDR < 0.1, ΔPSI > 10%; Fig. 5). These three events are transient receptor potential cation channel subfamily C member 3 (*Trpc3*) exon 9 (Fig. 5A), potassium calcium-activated channel subfamily M alpha 1 (*Kcnma1*) exon 23b (Fig. 5D) and ankyrin repeat and sterile alpha motif domain containing 1B (*Anks1b*) exon 5 (Fig. 5G). Interestingly, all three of these events showed brain region-specific splicing profiles (Fig. 5 and Supplementary Fig. 7). For wild-type mice, across cerebellar datasets *Trpc3* exon 9 was included in 25–51% of transcripts (Fig. 5B) whereas in cortical and brainstem regions it was included in >93% of transcripts (Supplementary Fig. 7A). Likewise, *Kcnma1* exon 23b was included in 16–33% of transcripts in cerebellum from wild-type mice (Fig. 5E), but in >83% of transcripts in cortical and brainstem regions (Supplementary Fig. 7B). Finally, exon 5 of *Anks1b* was included in <21% of transcripts from brainstem of wild-type mice, 81–83% of transcripts in cortex and striatum (Supplementary Fig. 7C) but included in 39–78% of transcripts in wild-type cerebellum (Fig. 5H). Notably, for each of these events, the short repeat length mice (SCA1: 30Q; SCA3: 15Q) showed no significant difference in splicing profiles compared to the age-matched wild-type mice (Fig. 5 and Supplementary Fig. 7). Due to the region-specific splicing profiles of these events, it is understandable that the different splice isoforms may represent unique functional adaptations for each brain region, which has been previously demonstrated for *Trpc3* exon 9^33,56^ and that deviations from these brain region-specific splicing profiles may incur detrimental functional consequences.

For *Trpc3* exon 9, there was a significant age-dependent increase in exon inclusion compared to wild-type mice across all nine SCA1 cerebellar datasets. At 5 weeks, ATXN1-82Q mice showed a 21.9% increase in exon 9 inclusion compared to wild-type mice, which increased to 39.7% by 12 weeks of age (FDR < 0.1). In the *Atxn1^154Q/2Q^* mice, 5-week mice showed a 9.1% increase in *Trpc3* exon 9 inclusion compared to wild-type mice (*P* < 0.05), which increased to 16.8% by 18 weeks (FDR < 0.1). A significant increase in exon 9 inclusion was also seen in 17.5-month MJD84.2 mice (*P* < 0.05; Fig. 5B). Using RT-PCR, we confirmed a 10.8% increase in *Trpc3* exon 9 inclusion in *Atxn1^154Q/2Q^* mice at 12 weeks of age (*P* < 0.0001) and a 11.3% increase in inclusion at 23 weeks of age (*P* = 0.0028; Fig. 5C and Supplementary Fig. 8A). Likewise, for *Kcnma1* exon 23b in ATXN1-82Q mice there was a minimum increase in exon inclusion of 15.3% compared to wild-type mice, which increased to 29.6% by 12 weeks (FDR < 0.1). In *Atxn1^154Q/2Q^* at 18 weeks of age there was a significant increase in exon 23b inclusion by 14.3% (FDR < 0.1) and in the SCA7 140Q/5Q mouse model exon 23b was included 9.4% of the time more than wild-type mice (*P* < 0.05; Fig. 5E). RT-PCR validation detected an 8.7% increase in exon 23b inclusion in 12-week-old *Atxn1^154Q/2Q^* mice compared to wild-type (*P* = 0.0009; Fig. 5F and Supplementary Fig. 8B). For *Anks1b* exon 5 in ATXN1-82Q mice a reduction in exon inclusion of 10.8% was observed at 5 weeks compared to wild-type mice, this increased to 30.5% at 12 weeks (FDR < 0.01). Eighteen week old *Atxn1^154Q/2Q^* mice showed a reduction in exon inclusion by 6.8% (*P* < 0.05) and both late-stage 17.5-month-old MJD84.2 mice (5.0%, *P* < 0.05) and SCA7 140Q/5Q mice (18.3%, FDR < 0.1) showed a significant reduction in exon inclusion (Fig. 5H). RT-PCR validation showed a 7.4% reduction in *Anks1b* exon 5 inclusion in 12-week-old *Atxn1^154Q/2Q^* mice (*P* < 0.0001), a 2.8% reduction in inclusion at 6 weeks (*P* = 0.0117) and a 4.8% reduction in inclusion at 23 weeks compared to wild-type mice (*P* = 0.0005; Fig. 5I and Supplementary Fig. 8C).

While we observed significant, age-dependent increase in dysregulation across SCA1, interestingly few of these events were specifically dysregulated across the SCA3 datasets. To investigate skipped exon events specifically dysregulated in SCA3, we tracked specific events dysregulated in the presymptomatic SCA3 dataset across disease progression. Of the skipped exon events identified, many were dysregulated in the same direction (inclusion or exclusion), showed missplicing across both the 304Q/304Q and the MJD84.2 mouse models and showed significant missplicing between the 15Q and MJD84.2 mice (Supplementary Fig. 9A). Of these events, three occurred in the same gene: brain enriched myelin associated protein 1 (*Bcas1*). *Bcas1* exon 10 was misspliced in two different contexts (exons 9-10-11 and exons 9-10-12) with both showing an increase in exon exclusion (Supplementary Fig. 9A–D). The greatest missplicing of these two events was exon 10 in the context of exons 9 and 12, which showed an increase in exclusion of between 15.4% and 23.5% across the datasets (FDR < 0.1; Supplementary Fig. 9C). *Bcas1* exon 9 showed an increase in exon inclusion of 9.1% in presymptomatic 304Q mice to 15.0% in symptomatic 304Q mice (FDR < 0.1; Supplementary Fig. 9D). Additionally, roundabout guidance receptor 1 (*Robo1*) exon 18 and peroxisomal biogenesis factor 5 like (*Pex5 l*) exon 2 both showed increases in exon inclusion in presymptomatic mice (FDR < 0.1) and were consistently dysregulated in at least three of the four datasets (Supplementary Fig. 9E and F). This pattern of shared, presymptomatic, repeat-length dependent missplicing suggests that alternative splicing dysregulation of key events could represent drivers of disease pathogenesis across SCA3 models.

Finally, we wanted to understand the possible contribution of alternative splicing to brainstem-associated defects in CAG expansion SCAs. Interestingly, of the nine events misspliced in five or six cortical or brainstem region datasets (Fig. 2D), two were *Bcas1* exon 9 (Supplementary Fig. 7D) and exon 10, with exon exclusion in the latter occurring in the same two contexts (Supplementary Fig. 7E and F) as with cerebellar SCA3 datasets (Supplementary Fig. 9B, C and G). Again, we saw an increase in inclusion of exon 9, which occurred across almost all datasets excluding the *ATXN1*-82Q inferior olive datasets. Increase in *Bcas1* exon 9 inclusion was seen in both medulla (18 and 28 weeks) and pons (28 weeks) of *Atxn1^154Q/2Q^* mice as well as across all SCA3 mouse models and brain regions but not in the 15Q versus wild-type mice comparison (Supplementary Fig. 7D). Similarly, for both exon 10 events, we observed a reduction in exon inclusion in almost all datasets excluding the *ATXN1*-82Q and 15Q versus wild-type mice (Supplementary Fig. 7E and F). Together, these data demonstrate that specific events are misspliced across different mouse models of SCA1, 3 and 7 and that this missplicing can occur in a cerebellar-specific manner or across cerebellum and brainstem. Furthermore, the genes affected by these alternative splicing events are relevant to SCA disease pathogenesis and pathological hallmarks.

### Alternative splicing could be used as a target engagement biomarker for CAG expansion SCAs

**Figure 6:**
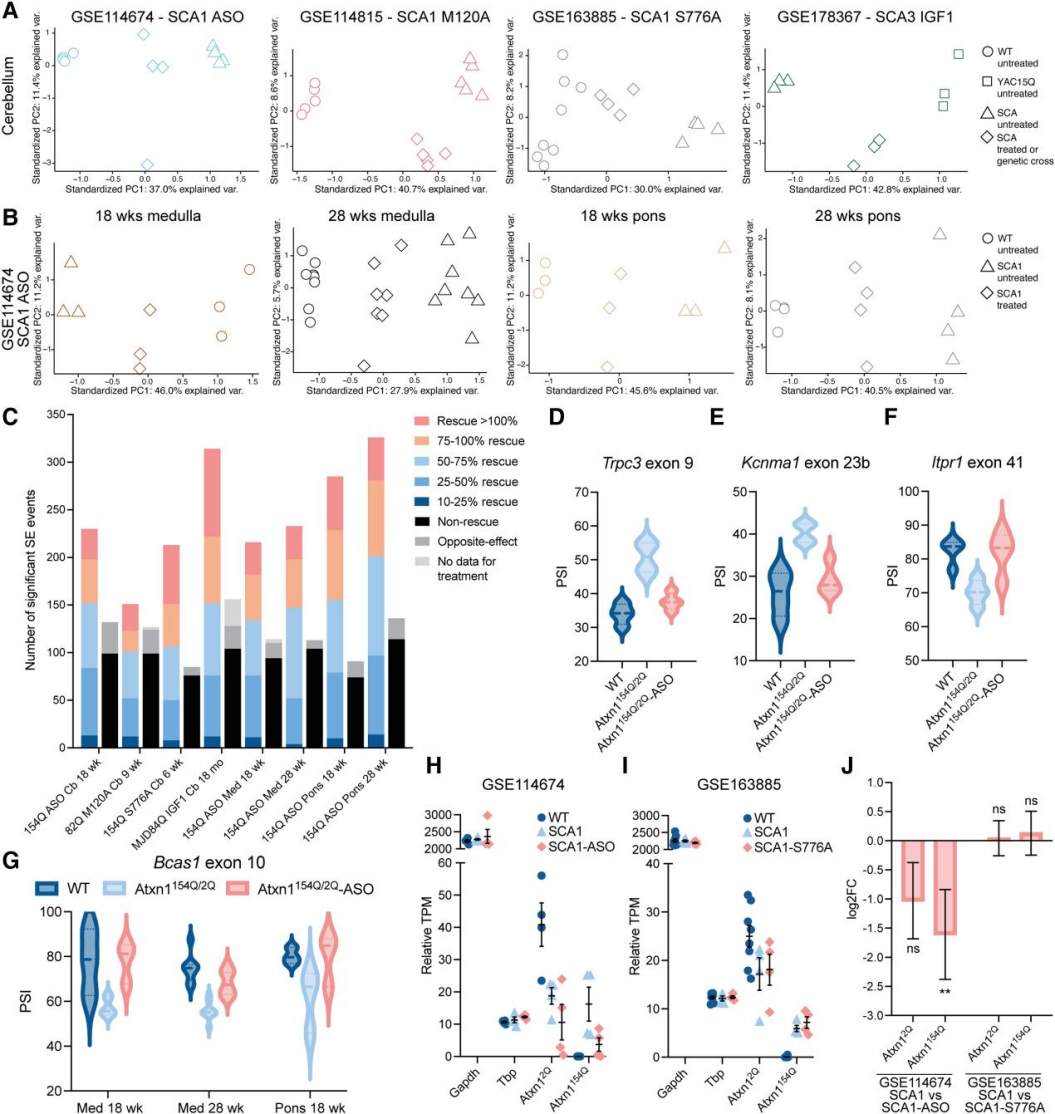
**Alternative splicing dysregulation is rescued in therapeutic studies of CAG expansion SCAs.** (**A** and **B**) Principal component analyses for skipped exon (SE) events significantly alternatively spliced (FDR < 0.1; ΔPSI > 10%) in spinocerebellar ataxia (SCA) versus wild-type (WT) cerebellum (**A**) or brainstem (**B**) datasets including therapeutic condition or genetic crosses with SCA mice (see Table 1). (**C**) Number of skipped exon events rescued in each dataset for all events significantly alternatively spliced between wild-type and SCA mice. Rescue is defined as >10% rescue (change in splicing towards wild-type PSI) in SCA treated versus SCA untreated mice with a minimum ΔPSI of 5% between SCA treated and untreated. Opposite effect is defined as >10% change in splicing away from wild-type PSI with a minimum ΔPSI of 5% between SCA treated and untreated. All events not classified as rescued or opposite effect are considered non-rescue events unless no information is available for the events in the treatment condition. (**D**–**F**) *Trpc3* exon 9, *Kcnma1* exon 23b and *Itpr1* exon 41 show significant rescue in splicing in *Atxn1^154Q/2Q^* mice treated with Atxn1 targeting ASO compared to untreated mice; rescue >10%, ΔPSI > 5%; (**D** and **F**) FDR < 0.1; (**E**) *P* < 0.05, FDR = 0.1106; thick line indicates median, thin dotted lines indicate quartiles. (**G**) Atxn1 ASO treatment rescues *Bcas1* 10 missplicing in medulla and pons of *Atxn1^154Q/2Q^* mice; rescue > 10%, ΔPSI > 5%, medulla datasets FDR < 0.1, pons 18 week *P <* 0.05, FDR = 0.1393; data shown for datasets with significant missplicing of *Bcas1* exon 10 (exons 9-10-12) at FDR < 0.1, ΔPSI > 10% between wild-type and *Atxn1^154Q/2Q^* mice (see Supplementary Fig. 7D). (**F**) Thick line indicates median, thin dotted lines indicate quartiles. (**H** and **I**) Relative TPM for *Gapdh*, *Tbp*, *Atxn1^2Q^* and *Atxn1^154Q^* in WT, SCA1 and SCA1-ASO (**H**) or SCA1-S776A (**I**) mice; data presented as mean relative TPM ± SE. (**J**) Log2FC ± SE for SCA1 versus SCA1-ASO or SCA1-S776A for *Atxn1^2Q^* and *Atxn1^154Q^* alleles; ns = not significant, **P < 0.01. FDR = false discovery rate; PSI = per cent spliced in.

We next sought to understand whether dysregulation of alternative splicing could be used as a target engagement biomarker for therapeutic studies in CAG expansion SCAs. To assess biomarker potential, we performed alternative splicing analyses across datasets generated from various therapeutic intervention studies (Table 1). These datasets included an *Atxn1* targeting antisense oligonucleotide (ASO) for SCA1,^36,43^ insulin-like growth factor 1 treatment in SCA3^39^ and two studies based on genetic manipulation to reduce phosphorylation of serine 776 in Atxn1^42,44^ (Table 1). Of these latter two studies, one had a serine to alanine mutation at position 776 (S776A) in the *Atxn1^154Q^* allele (study GSE163885)^42^ and the other had a mutation of a key amino acid (M120A) in the enzyme (PKA Cα) responsible for phosphorylating serine 776 in the ATXN1[82Q] mouse model (study GSE114815) ^44^; both these mouse models showed reduced levels of expanded ataxin1 protein. For all studies, the PSI values were calculated for the treatment condition for all skipped exon events significantly alternatively spliced (FDR < 0.1, ΔPSI > 10%) between control (WT or 15Q) and SCA mice (Supplementary Table 11); we then performed PCA based on these events. Across all eight datasets analysed there was clear separation between the three conditions (control, SCA, SCA-treated), for seven of the eight datasets, this separation occurred primarily in the PC1 direction and for six of these datasets the SCA-treatment mice were approximately equidistant between the control and SCA mice (Fig. 6A and B). The major exception to this was the ATXN1[82Q]-M120A dataset in which the SCA-genetic cross mice clustered distinct from, but close to, the SCA mice with the wild-type mice separated along PC1 from both these clusters (Fig. 6A). Overall, this analysis demonstrates that therapeutic strategies can mitigate alternative splicing dysregulation but that this response occurs to different extents depending on the treatment or genetic manipulation strategy.

To investigate the extent of splicing rescue in each dataset, we next categorized the skipped exon events (control versus SCA: FDR < 0.1, ΔPSI > 10%) based on the size of change (per cent rescue) between the SCA and SCA treatment mice. Events were classified as rescued if the PSI for SCA-treatment changed >10% in the direction of the control mice PSI value and had a ΔPSI > 5% compared to SCA mice. Events were classified as changed in the opposite direction if the ΔPSI > 5% for SCA-treatment versus SCA mice and there was >10% shift in PSI in the opposite direction to the control mice. Events that were not rescued had a ΔPSI < 5% between SCA-treatment and SCA mice or showed <10% rescue. For all datasets except ATXN1[82Q]-M120A, the events that were not rescued or showed an opposite effect represented approximately a quarter to a third of the events per dataset. For the ATXN1[82Q]-M120A dataset, the numbers of rescued and not-rescued/opposite effect events were comparable (Fig. 6C).

Next, we further filtered these skipped exon events to identify events significantly alternatively spliced between SCA and SCA-treated conditions and assessed whether key SCA splicing changes were rescued. We focused on the SCA1-ASO datasets and the ATXN1[82Q]-M120A dataset as these showed significant dysregulation of core SCA splicing changes with an FDR < 0.1 and ΔPSI > 10% for wild-type versus SCA mice (Fig. 5 and Supplementary Fig. 7). In the SCA1 cerebellar ASO dataset, we observed a 78% rescue of *Trpc3* exon 9 missplicing (FDR < 0.1; Fig. 6D) and a 77% rescue of *Kcnma1* exon 23b missplicing (*P* < 0.05; Fig. 6E) following Atxn1 ASO treatment. In addition, we assessed rescue of *Itpr1* exon 41, which was significantly misspliced (FDR < 0.1, ΔPSI > 10%) in five of 13 cerebellar datasets (Fig. 1D and Supplementary Table 4) and found that the missplicing was rescued by 96% (FDR < 0.1, Fig. 6F). Of all the cerebellar treatment datasets, ATXN1[82Q]-M120A was the only dataset that showed significant changes in splicing in the opposite direction to control mice (Supplementary Table 11). For example, *Camk2a* exon 14 showed a 78% exacerbation of missplicing (FDR < 0.1; Supplementary Fig. 10A). In contrast to treatment with the Atxn1 ASO, mutation of PKA Cα M120A did not rescue *Trpc3* exon 9 or *Kcnma1* exon 23b missplicing (Supplementary Fig. 10B and C) but did lead to a 35% rescue of *Itpr1* exon 41 missplicing (FDR < 0.1, Supplementary Fig. 10D). Finally, we identified that Atxn1 ASO treatment significantly rescued *Bcas1* exon 9 and exon 10 missplicing in the medulla and pons with a minimum rescue of 66% for exon 10 (Fig. 6G) and 82% for exon 9 (Supplementary Fig. 10E). Together these data demonstrate that alternative splicing dysregulation can be used as a transcriptomic readout in therapeutic studies for CAG expansion SCAs.

To investigate the relationship between CAG expansion expression and alternative splicing we took advantage of the sequence differences between the 2Q and 154Q alleles^57^ present in the same mouse, to distinguish between the alleles bioinformatically. To confirm these sequence differences, we performed a PCR across the repeat expansion and sequenced the 2Q and 154Q alleles (Supplementary Fig. 11A–C). To validate the specificity of using these sequence differences to distinguish between the 154Q and 2Q alleles, we designed qPCR primers selective for each allele (Supplementary Fig. 11A). The 154Q-allele selective primers did not amplify a product in wild-type mice (Supplementary Fig. 11D and E) and while there was no difference in the total levels of *Atxn1* RNA between wild-type and *Atxn1^154Q/2Q^* mice, as expected, the 2Q-allele selective primers detected a 52% reduction in *Atxn1^2Q^* RNA in *Atxn1^154Q/2Q^* mice compared to wild-type mice (*P* = 0.0019; Supplementary Fig. 11D and F).

Having confirmed the specificity of this approach using qPCR, we generated a custom genome containing sequences, of the same length, specific to the 2Q and 154Q alleles. To account for read length, unique genomes were used for datasets with different read lengths such that every read would have to overlap with at least three of the sequence differences to align to either allele. Because of this, relative TPMs for *Atxn1* alleles are not comparable between datasets and do not represent TPMs for full length *Atxn1*. Comparable to the qPCR approach, the 154Q allele was not detected in wild-type mice from datasets GSE114674 and GSE163885, and SCA1 mice showed ∼30–55% less expression of the 2Q allele in both datasets (GSE114674: WT 40.84 ± 13.44, SCA1 18.77 ± 5.13, Fig. 6H; GSE163885: WT 24.99 ± 6.29, SCA1 17.19 ± 6.717; Fig. 6I). Housekeeping genes did not show differences between WT, SCA1 and SCA1 treated mice within each dataset (Fig. 6H and I). While no difference was seen in expression for either the *Atxn1^2Q^* or *Atxn1^154Q^* alleles for SCA1 versus SCA1-S776A mice, both alleles showed a reduction in expression (*Atxn1^2Q^*: log2FC = −1.03; *P* = 0.12) with significant reduction of the *Atxn1^154Q^* allele in the SCA1-ASO mice compared to the wild-type mice (*Atxn1^154Q^*: log2FC = −1.61; *P* = 0.037; Fig. 6J). Together these data demonstrate that expression of *Atxn1^2Q^* and *Atxn1^154Q^* alleles can be selectively quantified and that alternative splicing dysregulation can be rescued by reducing levels of CAG expansion RNAs or by reducing expansion protein levels, as is the case for GSE163885.^42^ These data suggest that alternative splicing dysregulation may represent a tractable biomarker for preclinical therapeutic studies in CAG expansion SCAs.

## Discussion

Dysregulation of alternative splicing has been identified and extensively studied as a key driver of disease pathogenesis in the CTG repeat expansion disorder myotonic dystrophy.^8,10,14^ Despite evidence implicating splicing changes in multiple CAG expansion disorders,^16-21^ the extent of alternative splicing dysregulation and its possible contribution to disease across CAG expansion SCAs is poorly understood. Here, we identified that dysregulation of alternative splicing is widespread across CAG expansion mouse models of SCAs 1, 3 and 7. These changes were detected presymptomatically, persisted throughout disease and were present in the cerebellum and other brain regions implicated in SCA pathogenesis, including the pons and medulla. Widespread alternative splicing dysregulation was also detected when CAG expansion SCA mice were compared to mouse models expressing non-pathogenic length repeat tracts. Furthermore, these differences in alternative splicing were comparable in magnitude to the differences between CAG expansion SCA and wild-type mice. We demonstrated that across disease progression, changes in alternative splicing occurred in genes that function in pathways known to be impaired in SCAs, such as ion channels, synaptic signalling and the cytoskeleton. Finally, we demonstrate that alternative splicing can be used as a target engagement biomarker with key CAG SCA alternative splicing events rescued by *Atxn1* targeting ASO.

**Figure 7:**
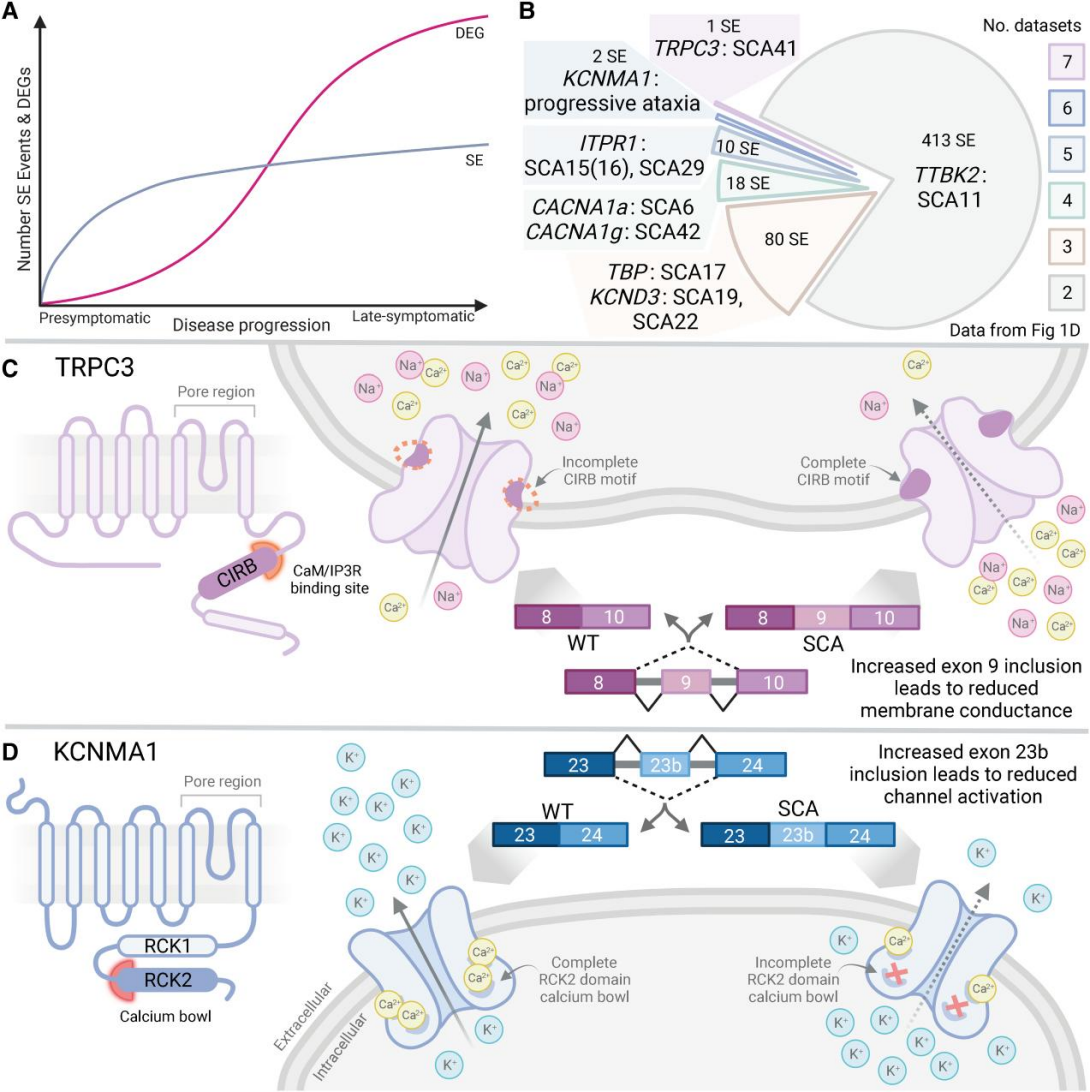
**Potential functional consequences and disease relevance of missplicing in CAG expansion SCAs.** (**A**) Temporal overview of transcriptional dysregulation across mouse models. Alternatively spliced skipped exon events (SE, blue) are dysregulated presymptomatically in SCA mouse models. At the transition to symptomatic stages of disease, the number of differentially expressed genes (DEGs, pink) increases. Skipped exon alternative splicing events see a more-tempered increase in number than DEGs as disease progresses. (**B**) Overview of the number of misspliced skipped exon events shared between multiple cerebellar datasets highlighting genes known to cause SCAs that have misspliced skipped exons across two more datasets in our analysis (SE events: FDR < 0.1, ΔPSI > 10%). Data recreated from Fig. 1D. (**C**) In SCA models, there is increased inclusion of exon 9 in transient receptor potential cation channel subfamily C member 3 (*Trpc3*); in wild-type (WT) mice, exclusion of exon 9 is a cerebellar specific alternative splicing event. Exon 9 spans the calmodulin (CaM) and inositol trisphosphate receptor (IP3R) binding (CIRB) motif (highlighted in orange). When translated, the complete CIRB motif induces a conformational change in the channel that reduces activation and subsequent Ca^2+^ influx. This results in reduced neuronal GPCR-Ca^2+^ signalling efficiency and reduced membrane conductance. (**D**) In SCA models, there is increased inclusion of exon 23b in potassium calcium-activated channel subfamily M alpha 1 (*Kcnma1*); in wild-type mice, exclusion of exon 23b is a cerebellar specific alternative splicing event. Exon 23b partially spans the regulator of K^+^ conductance (RCK2) domain and the calcium bowl region, a high-affinity Ca^2+^ binding site (highlighted in red). When translated, exon 23b inclusion impairs Ca^2+^ binding in the calcium bowl causing a reduction in channel activation and a reduction in the subsequent K^+^ efflux. This reduced calcium sensing capability results in slower synapse repolarization. (**A**–**D**) This figure was generated using BioRender.com. FDR = false discovery rate; PSI = per cent spliced in.

One of the key findings of this study is the presence of widespread alternative splicing dysregulation prior to global changes in differential gene expression (Fig. 7A). While previous studies have demonstrated widespread differential gene expression changes from early symptomatic stages of disease onwards, differential gene expression studies have so far failed to identify consistent, presymptomatic drivers of disease at the transcriptomic level.^35,37,38^ Here we demonstrate presymptomatic missplicing of multiple skipped exon events that could directly contribute to neuronal dysfunction prior to degenerative changes. These changes in alternative splicing were also seen between short repeat and expanded repeat mice at presymptomatic and early symptomatic stages of disease when differential gene expression changes were limited.^38^ Importantly, at both pre- and early symptomatic time points in inferior olive of *ATXN1*-82Q mice, we did not detect widespread missplicing and, of the changes seen, there was very limited overlap seen with other datasets. Since the *ATXN1* transgene is only expressed in Purkinje neurons in this mouse model,^35,55^ this finding indicates that alternative splicing dysregulation is likely not a late, downstream response to disease and that it is primarily governed by cell autonomous effects. Together these data demonstrate that alternative splicing dysregulation is a repeat length-dependent transcriptomic hallmark of CAG expansion SCAs and may represent a presymptomatic driver of disease pathogenesis in these disorders.

Given that dysregulated skipped exon events were primarily found in genes linked to pathways known to be disrupted in CAG expansion SCAs, it is plausible that dysregulation of these alternatively spliced events could contribute to the disruption of these interrelated pathways. Interestingly, some pathways were affected in subgroups of the datasets studied indicating disease stage, brain region or SCA type-specific effects of missplicing. For example, cell adhesion was enriched for presymptomatic cerebellar datasets and nuclear process associated terms were more frequently associated with SCA3 or brainstem datasets than in SCA1 cerebellar datasets. The latter of these findings may reflect a contribution of the early, skipped exon missplicing in zinc fingers and transcriptional regulators to the subsequent widespread dysregulation of gene expression, but accounts for the strong link between alterations in mutant Atxn1 interactions with the transcriptional repressor capicua and differential gene expression in SCA1 cerebellum.^3^ Conversely, enrichment of cytoskeleton and neuron projection associated terms occurred across all datasets. Numerous avenues of evidence suggesting that cytoskeletal disruption occurs in ataxias,^58-64^ most notably, mutations in the β-III spectrin gene cause SCA5^59^ and impairs spectrin-actin cytoskeletal dynamics and dendritic arborization.^58^ We identified dysregulation of alternative splicing of *Anks1b* which acts as a scaffold protein linking the membrane and cytoskeleton at postsynaptic densities.^65^ While studies have demonstrated a role for Anks1b in glutamatergic neurotransmission and synaptic plasticity,^65^ other Ankyrin cytoskeletal scaffolding proteins have been shown to be essential for Purkinje neuron survival by connecting potassium channels to the β-III spectrin cytoskeleton.^66^ Similarly, across datasets from brainstem and SCA3 cerebellum, we saw dysregulation of alternative splicing of *Bcas1*, a marker of early myelinating oligodendrocytes of which loss causes hypomyelination.^67,68^ Interestingly, defects in early oligodendrocyte maturation have been implicated in SCA3^69,70^ with a reduction in myelination seen across multiple CAG SCAs.^70-74^ These data indicate that, at the level of pathways and individual genes, dysregulation of alternative splicing could contribute to disruption of cellular processes known to be implicated in pathogenesis of ataxia.

While the missplicing of *Anks1b* and *Bcas1* suggest possible functional links to pathways impaired in SCAs, the relevance of the individual splicing events is not clear; however, for several of the skipped exon events identified in ion channels the specific functional effect of the missplicing is well defined. Two key events that were identified and validated in this study occurred in ion channels in which mutations have been shown to cause ataxia: *Trpc3* mutations cause SCA41^4,5^ and mutations in *Kcnma1* cause progressive ataxia^4,75,76^ (Fig. 7B). We also detected missplicing of skipped exon events in an additional six genes known to cause eight different types of SCA,^2,4^ in two or more cerebellar datasets (Fig. 7B). In *Trpc3* we found that exon 9 is included more often in SCA cerebellum than wild-type cerebellum. Normally, exon 9 is excluded ∼70% of the time in the cerebellum, which leads to Trpc3 proteins with incomplete CIRB domains and increased membrane conductance due to enhanced channel opening frequency and increased Ca^2+^ entry.^33,56^ In SCAs, this cerebellar specific adaptation is disrupted by an increase in exon 9 inclusion, which would lead to reduced Ca^2+^ entry and decreased membrane conductance (Fig. 7C). Likewise, an increase in inclusion of exon 23b in *Kcnma1* in SCA cerebellum leads to an incomplete calcium bowl, a region within the RCK2 domain, which impairs Ca^2+^ binding, reduces channel activation and K^+^ efflux—in turn, slowing synapse repolarization^77,78^ (Fig. 7D). Importantly, impaired calcium signalling, disrupted membrane conductance and impaired K^+^ channel functioning have previously been reported in CAG expansion SCAs.^2,4^ These specific events highlight how missplicing could directly impair cerebellar-specific adaptations and functions of ion channels encoded for by genes which harbour mutations that cause ataxia.

In addition to genetic causes of ataxia, there are also several forms of paraneoplastic cerebellar degeneration which cause ataxia and are associated with antineuronal antibodies produced by cancers, which target specific proteins or cells.^79^ One of these immunological forms of ataxia is caused by anti-Ri antibodies, which target NOVA alternative splicing regulator-1 (NOVA1) and NOVA2.^79,80^ The NOVA proteins are neuron-specific RNA binding proteins that regulate alternative splicing of specific events with critical roles in axon projections, voltage-gated ion channel regulation, and synapse structure and function, with NOVA1 being predominantly expressed in the cerebellum and spinal cord.^81^ In our study, splicing factor NOVA regulated synaptic proteins was one of the most frequently dysregulated gene ontology enrichment terms across all datasets, and the most enriched term in presymptomatic SCA1 datasets and comparisons between short and long repeat length mouse models. While this finding indicates overlap between alternative splicing in CAG expansion SCAs and another form of ataxia, it also suggests the potential for NOVA RBPs to be implicated in the mechanism of alternative splicing in CAG expansion SCAs.

Although further studies are needed to understand the mechanism(s) of widespread alternative splicing dysregulation in CAG expansion SCAs, studies from other microsatellite expansion diseases indicate possible mechanisms that could contribute to the dysregulation of alternative splicing. For example, in DM1, the CUG expansion RNAs sequester the muscle-blind like (MBNL) family of RBPs into nuclear RNA foci. MBNL proteins regulate alternative splicing of specific MBNL-dependent skipped exon events. In DM1, this sequestration reduces the functional pool of MBNL proteins resulting in missplicing of MBNL-regulated alternative splicing events.^8,10^ This RNA-sequestration model of alternative splicing dysregulation is one possible mechanism through which widespread missplicing could be occurring in CAG SCAs. Indeed, this dysregulation could be mediated through either or both the sense CAG RNAs and antisense CUG RNAs, the latter of which is already supported by studies in SCA8^16^ and SCA2.^17^ However, other studies focusing on SCA3 and Huntington’s disease suggest that CAG expansion RNAs may play a role in alternative splicing dysregulation.^21^

Another possible mechanism supported by studies in Huntington’s disease is a reduction in expression of multiple RBPs,^18^ which would again lead to a reduction in the functional pool of RBPs or change in the balance of RBP expression and subsequent dysregulation of alternative splicing events mediated by these RBPs. Due to the clear implication of differential gene expression in CAG SCAs,^35,37,38,41,46,49^ effects on RBP expression may contribute to splicing dysregulation in later stages of disease progression. However, due to the detection of widespread alternative splicing dysregulation prior to widespread differential gene expression, this mechanism is unlikely to contribute to presymptomatic missplicing.

Finally, there is the possibility for a protein sequestration mechanism whereby aggregates of polyglutamine proteins^2,3^ or proteins produced through repeat-associated non-AUG (RAN) translation^82^ sequester splicing factors and RBPs.^83-85^ Polyglutamine and RAN proteins have been shown to be preferentially expressed and aggregate in different cell types and brain regions.^71,82,86^ As RBPs have brain region-specific expression profiles,^9,10,81^ the differential affinities of each expansion protein for specific RBPs could lead to brain region-specific protein sequestration resulting in the differences in alternative splicing seen in this study. Changes in expression of both expansion proteins and RBPs, their cellular localization or post-translational modifications could all potentially impact this mechanism of alternative splicing dysregulation. While this mechanism is supported by evidence that reducing Atxn1 expression and aggregation^42,44^ rescued missplicing to a certain extent (Fig. 6A, C and J and Supplementary Fig. 10A–D), further studies are needed to understand the contribution of protein and RNA mediated RBP sequestration, accounting for effects of polyglutamine and RAN proteins and sense and antisense RNAs, and identifying which RBPs are relevant for missplicing in CAG SCAs.

Here we identified dysregulation of alternative splicing as a novel presymptomatic pathogenic mechanism in CAG expansion SCAs by performing analyses of previously published RNA-Seq datasets. While confounding factors such as read-length, read-depth and limitations due to polyA selection,^25,50-52,54,87^ limit comparisons between datasets, this study highlights what can be learned from mining published data and the benefits of data-sharing. Future studies will be needed to determine whether alternative splicing dysregulation is a key transcriptomic hallmark in CAG expansion SCA patient derived-model systems and if misspliced transcripts could be used as target engagement biomarkers in CAG SCAs. It is important to note that the use of rRNA depletion methods with long read lengths and paired-end sequencing in these types of studies would enable a greater volume of information to be extracted about alternatively spliced exons and the maturation of alternatively spliced isoforms.^25,50-52,54^ Additionally, while we presented clear functional consequences for two of the identified splicing events, future studies will be required to understand the direct contribution of these alternative splicing events to disease symptoms, and will have to account for brain region specific missplicing. Together, these studies provide an overview of alternative splicing dysregulation in CAG expansion SCAs and represent a platform for future investigations into the mechanisms and therapeutic potential, both as biomarkers and therapeutic candidates, of alternative splicing dysregulation across this class of devastating diseases.

## Supplementary Material

awad329_Supplementary_Data

## Data Availability

All datasets used in this study are publicly available through the NCBI Gene Expression Omnibus (GEO) using the following GSE numbers: GSE122099, GSE114674, GSE108256, GSE75778, GSE114815, GSE163885, GSE107958, GSE117605, GSE145613, GSE178367, and GSE138527 (Table 1). Custom genome files used for analyses in Fig. 6H–J are available from the authors on request.

## References

[1] Ashizawa T , Öz G, Paulson HL. Spinocerebellar ataxias: Prospects and challenges for therapy development. Nat Rev Neurol. 2018;14:590–605. 30131520 10.1038/s41582-018-0051-6 PMC6469934

[2] Klockgether T , Mariotti C, Paulson HL. Spinocerebellar ataxia. Nat Rev Dis Primers. 2019;5:24. 30975995 10.1038/s41572-019-0074-3

[3] Paulson HL , Shakkottai VG, Clark HB, Orr HT. Polyglutamine spinocerebellar ataxias—From genes to potential treatments. Nat Rev Neurosci. 2017;18:613–626. 28855740 10.1038/nrn.2017.92 PMC6420820

[4] Bushart DD , Shakkottai VG. Ion channel dysfunction in cerebellar ataxia. Neurosci Lett. 2019;688:41–48. 29421541 10.1016/j.neulet.2018.02.005 PMC6077100

[5] Fogel BL , Hanson SM, Becker EBE. Do mutations in the murine ataxia gene TRPC3 cause cerebellar ataxia in humans? Mov Disord. 2015;30:284–286. 25477146 10.1002/mds.26096 PMC4318721

[6] Huang L , Chardon JW, Carter MT, et al Missense mutations in ITPR1 cause autosomal dominant congenital nonprogressive spinocerebellar ataxia. Orphanet J Rare Dis. 2012;7:67. 22986007 10.1186/1750-1172-7-67 PMC3545966

[7] Marelli C , van de Leemput J, Johnson JO, et al SCA15 Due to large ITPR1 deletions in a cohort of 333 white families with dominant ataxia. Arch Neurol. 2011;68:637–643. 21555639 10.1001/archneurol.2011.81 PMC3142680

[8] Hale MA , Johnson NE, Berglund JA. Repeat-associated RNA structure and aberrant splicing. Biochim Biophys Acta Gene Regul Mech. 2019;1862(11–12):194405. 31323433 10.1016/j.bbagrm.2019.07.006 PMC7099610

[9] Baralle FE , Giudice J. Alternative splicing as a regulator of development and tissue identity. Nat Rev Mol Cell Biol. 2017;18:437–451. 28488700 10.1038/nrm.2017.27 PMC6839889

[10] Scotti MM , Swanson MS. RNA mis-splicing in disease. Nat Rev Genet. 2016;17:19–32. 26593421 10.1038/nrg.2015.3 PMC5993438

[11] Mankodi A , Takahashi MP, Jiang H, et al Expanded CUG repeats trigger aberrant splicing of ClC-1 chloride channel pre-mRNA and hyperexcitability of skeletal muscle in myotonic dystrophy. Mol Cell. 2002;10:35–44. 12150905 10.1016/s1097-2765(02)00563-4

[12] Freyermuth F , Rau F, Kokunai Y, et al Splicing misregulation of SCN5A contributes to cardiac-conduction delay and heart arrhythmia in myotonic dystrophy. Nat Commun. 2016;7:11067. 27063795 10.1038/ncomms11067 PMC4831019

[13] Jenquin JR , O'Brien AP, Poukalov K, et al Molecular characterization of myotonic dystrophy fibroblast cell lines for use in small molecule screening. iScience. 2022;25:104198. 35479399 10.1016/j.isci.2022.104198 PMC9035709

[14] Wagner SD , Struck AJ, Gupta R, et al Dose-Dependent regulation of alternative splicing by MBNL proteins reveals biomarkers for myotonic dystrophy. PLoS Genet. 2016;12:e1006316. 27681373 10.1371/journal.pgen.1006316 PMC5082313

[15] Antoury L , Hu N, Balaj L, et al Analysis of extracellular mRNA in human urine reveals splice variant biomarkers of muscular dystrophies. Nat Commun. 2018;9:3906. 30254196 10.1038/s41467-018-06206-0 PMC6156576

[16] Daughters RS , Tuttle DL, Gao W, et al RNA gain-of-function in spinocerebellar ataxia type 8. PLoS Genet. 2009;5:e1000600. 19680539 10.1371/journal.pgen.1000600 PMC2719092

[17] Li PP , Sun X, Xia G, et al ATXN2-AS, A gene antisense to ATXN2, is associated with spinocerebellar ataxia type 2 and amyotrophic lateral sclerosis. Ann Neurol. 2016;80:600–615. 27531668 10.1002/ana.24761 PMC6555153

[18] Elorza A , Marquez Y, Cabrera JR, et al Huntington’s disease-specific mis-splicing unveils key effector genes and altered splicing factors. Brain. 2021;144:2009–2023. 33725094 10.1093/brain/awab087 PMC8370404

[19] Lin L , Park JW, Ramachandran S, et al Transcriptome sequencing reveals aberrant alternative splicing in Huntington’s disease. Hum Mol Genet. 2016;25:3454–3466. 27378699 10.1093/hmg/ddw187 PMC5179942

[20] Schilling J , Broemer M, Atanassov I, et al Deregulated splicing is a Major mechanism of RNA-induced toxicity in Huntington's disease. J Mol Biol. 2019;431:1869–1877. 30711541 10.1016/j.jmb.2019.01.034

[21] Mykowska A , Sobczak K, Wojciechowska M, Kozlowski P, Krzyzosiak WJ. CAG Repeats mimic CUG repeats in the misregulation of alternative splicing. Nucleic Acids Res. 2011;39:8938–8951. 21795378 10.1093/nar/gkr608 PMC3203611

[22] Dobin A , Davis CA, Schlesinger F, et al STAR: Ultrafast universal RNA-Seq aligner. Bioinformatics. 2013;29:15–21. 23104886 10.1093/bioinformatics/bts635 PMC3530905

[23] Robinson JT , Thorvaldsdottir H, Winckler W, et al Integrative genomics viewer. Nat Biotechnol. 2011;29:24–26. 21221095 10.1038/nbt.1754 PMC3346182

[24] Love MI , Huber W, Anders S. Moderated estimation of fold change and dispersion for RNA-Seq data with DESeq2. Genome Biol. 2014;15:550. 25516281 10.1186/s13059-014-0550-8 PMC4302049

[25] Shen S , Park JW, Lu ZX, et al rMATS: Robust and flexible detection of differential alternative splicing from replicate RNA-Seq data. Proc Natl Acad Sci U S A. 2014;111:E5593–E5601. 25480548 10.1073/pnas.1419161111 PMC4280593

[26] Zhou Y , Zhou B, Pache L, et al Metascape provides a biologist-oriented resource for the analysis of systems-level datasets. Nat Commun. 2019;10:1523. 30944313 10.1038/s41467-019-09234-6 PMC6447622

[27] Huang da W , Sherman BT, Lempicki RA. Systematic and integrative analysis of large gene lists using DAVID bioinformatics resources. Nat Protoc. 2009;4:44–57. 19131956 10.1038/nprot.2008.211

[28] Sherman BT , Hao M, Qiu J, et al DAVID: A web server for functional enrichment analysis and functional annotation of gene lists (2021 update). Nucleic Acids Res. 2022;50(W1):W216–W221. 35325185 10.1093/nar/gkac194 PMC9252805

[29] Shannon P , Markiel A, Ozier O, et al Cytoscape: A software environment for integrated models of biomolecular interaction networks. Genome Res. 2003;13:2498–2504. 14597658 10.1101/gr.1239303 PMC403769

[30] Bray NL , Pimentel H, Melsted P, Pachter L. Near-optimal probabilistic RNA-Seq quantification. Nat Biotechnol. 2016;34:525–527. 27043002 10.1038/nbt.3519

[31] Pimentel H , Bray NL, Puente S, Melsted P, Pachter L. Differential analysis of RNA-Seq incorporating quantification uncertainty. Nat Methods. 2017;14:687–690. 28581496 10.1038/nmeth.4324

[32] Watase K , Weeber EJ, Xu B, et al A long CAG repeat in the mouse sca1 locus replicates SCA1 features and reveals the impact of protein solubility on selective neurodegeneration. Neuron. 2002;34:905–919. 12086639 10.1016/s0896-6273(02)00733-x

[33] Kim Y , Wong AC, Power JM, et al Alternative splicing of the TRPC3 ion channel calmodulin/IP3 receptor-binding domain in the hindbrain enhances cation flux. J Neurosci. 2012;32:11414–11423. 22895723 10.1523/JNEUROSCI.6446-11.2012 PMC6621195

[34] Livak KJ , Schmittgen TD. Analysis of relative gene expression data using real-time quantitative PCR and the 2(-Delta Delta C(T)) method. Methods. 2001;25:402–408. 11846609 10.1006/meth.2001.1262

[35] Driessen TM , Lee PJ, Lim J. Molecular pathway analysis towards understanding tissue vulnerability in spinocerebellar ataxia type 1. Elife. 2018;7:e39981. 30507379 10.7554/eLife.39981 PMC6292693

[36] Friedrich J , Kordasiewicz HB, O'Callaghan B, et al Antisense oligonucleotide-mediated ataxin-1 reduction prolongs survival in SCA1 mice and reveals disease-associated transcriptome profiles. JCI Insight. 2018;3:e123193. 30385727 10.1172/jci.insight.123193 PMC6238731

[37] Haas E , Incebacak RD, Hentrich T, et al A novel SCA3 knock-in mouse model mimics the human SCA3 disease phenotype including neuropathological, behavioral, and transcriptional abnormalities especially in oligodendrocytes. Mol Neurobiol. 2022;59:495–522. 34716557 10.1007/s12035-021-02610-8 PMC8786755

[38] Ingram M , Wozniak EAL, Duvick L, et al Cerebellar transcriptome profiles of ATXN1 transgenic mice reveal SCA1 disease progression and protection pathways. Neuron. 2016;89:1194–1207. 26948890 10.1016/j.neuron.2016.02.011 PMC4795980

[39] Lin YT , Lin YS, Cheng WL, et al Transcriptomic and metabolic network analysis of metabolic reprogramming and IGF-1 modulation in SCA3 transgenic mice. Int J Mol Sci. 2021;22:7974. 34360740 10.3390/ijms22157974 PMC8348158

[40] Liu Q , Huang S, Yin P, et al Cerebellum-enriched protein INPP5A contributes to selective neuropathology in mouse model of spinocerebellar ataxias type 17. Nat Commun. 2020;11:1101. 32107387 10.1038/s41467-020-14931-8 PMC7046734

[41] Niewiadomska-Cimicka A , Doussau F, Perot JB, et al SCA7 Mouse cerebellar pathology reveals preferential downregulation of key purkinje cell-identity genes and shared disease signature with SCA1 and SCA2. J Neurosci. 2021;41:4910–4936. 33888607 10.1523/JNEUROSCI.1882-20.2021 PMC8260160

[42] Nitschke L , Coffin SL, Xhako E, et al Modulation of ATXN1 S776 phosphorylation reveals the importance of allele-specific targeting in SCA1. JCI Insight. 2021;6:e144955. 33554954 10.1172/jci.insight.144955 PMC7934855

[43] O’Callaghan B , Hofstra B, Handler HP, et al Antisense oligonucleotide therapeutic approach for suppression of ataxin-1 expression: A safety assessment. Mol Ther Nucleic Acids. 2020;21:1006–1016. 32818920 10.1016/j.omtn.2020.07.030 PMC7452125

[44] Pérez Ortiz JM , Mollema N, Toker N, et al Reduction of protein kinase A-mediated phosphorylation of ATXN1-S776 in Purkinje cells delays onset of ataxia in a SCA1 mouse model. Neurobiol Dis. 2018;116:93–105. 29758256 10.1016/j.nbd.2018.05.002 PMC6028938

[45] Pflieger LT , Dansithong W, Paul S, et al Gene co-expression network analysis for identifying modules and functionally enriched pathways in SCA2. Hum Mol Genet. 2017;26:3069–3080. 28525545 10.1093/hmg/ddx191 PMC5886232

[46] Ramani B , Panwar B, Moore LR, et al Comparison of spinocerebellar ataxia type 3 mouse models identifies early gain-of-function, cell-autonomous transcriptional changes in oligodendrocytes. Hum Mol Genet. 2017;26:3362–3374. 28854700 10.1093/hmg/ddx224 PMC5886175

[47] Rousseaux MWC , Tschumperlin T, Lu HC, et al ATXN1-CIC Complex is the primary driver of cerebellar pathology in spinocerebellar ataxia type 1 through a gain-of-function mechanism. Neuron. 2018;97:1235–1243 e5. 29526553 10.1016/j.neuron.2018.02.013 PMC6422678

[48] Stoyas CA , Bushart DD, Switonski PM, et al Nicotinamide pathway-dependent sirt1 activation restores calcium homeostasis to achieve neuroprotection in spinocerebellar ataxia type 7. Neuron. 2020;105:630–644 e9. 31859031 10.1016/j.neuron.2019.11.019 PMC7147995

[49] Toonen LJA , Overzier M, Evers MM, et al Transcriptional profiling and biomarker identification reveal tissue specific effects of expanded ataxin-3 in a spinocerebellar ataxia type 3 mouse model. Mol Neurodegener. 2018;13:31. 29929540 10.1186/s13024-018-0261-9 PMC6013885

[50] Shen S , Park JW, Huang J, et al MATS: A Bayesian framework for flexible detection of differential alternative splicing from RNA-Seq data. Nucleic Acids Res. 2012;40:e61. 22266656 10.1093/nar/gkr1291 PMC3333886

[51] Trincado JL , Entizne JC, Hysenaj G, et al SUPPA2: Fast, accurate, and uncertainty-aware differential splicing analysis across multiple conditions. Genome Biol. 2018;19:40. 29571299 10.1186/s13059-018-1417-1 PMC5866513

[52] Vaquero-Garcia J , Barrera A, Gazzara MR, et al A new view of transcriptome complexity and regulation through the lens of local splicing variations. Elife. 2016;5:e11752. 26829591 10.7554/eLife.11752 PMC4801060

[53] Reddy K , Jenquin JR, McConnell OL, et al A CTG repeat-selective chemical screen identifies microtubule inhibitors as selective modulators of toxic CUG RNA levels. Proc Natl Acad Sci U S A. 2019;116:20991–21000. 31570586 10.1073/pnas.1901893116 PMC6800345

[54] Katz Y , Wang ET, Airoldi EM, Burge CB. Analysis and design of RNA sequencing experiments for identifying isoform regulation. Nat Methods. 2010;7:1009–1015. 21057496 10.1038/nmeth.1528 PMC3037023

[55] Burright EN , Clark HB, Servadio A, et al SCA1 Transgenic mice: A model for neurodegeneration caused by an expanded CAG trinucleotide repeat. Cell. 1995;82:937–948. 7553854 10.1016/0092-8674(95)90273-2

[56] Cederholm JME , Kim Y, von Jonquieres G, Housley GD. Human brain region-specific alternative splicing of TRPC3, the type 3 canonical transient receptor potential non-selective cation channel. Cerebellum. 2019;18:536–543. 30887370 10.1007/s12311-019-01026-4

[57] Lorenzetti D , Watase K, Xu B, Matzuk MM, Orr HT, Zoghbi HY. Repeat instability and motor incoordination in mice with a targeted expanded CAG repeat in the sca1 locus. Hum Mol Genet. 2000;9:779–785. 10749985 10.1093/hmg/9.5.779

[58] Avery AW , Thomas DD. Hays TS. beta-III-spectrin spinocerebellar ataxia type 5 mutation reveals a dominant cytoskeletal mechanism that underlies dendritic arborization. Proc Natl Acad Sci U S A. 2017;114:E9376–E9385. 29078305 10.1073/pnas.1707108114 PMC5676893

[59] Ikeda Y , Dick KA, Weatherspoon MR, et al Spectrin mutations cause spinocerebellar ataxia type 5. Nat Genet. 2006;38:184–190. 16429157 10.1038/ng1728

[60] Smith FM , Kosman DJ. Molecular defects in Friedreich’s ataxia: Convergence of oxidative stress and cytoskeletal abnormalities. Front Mol Biosci. 2020;7:569293. 33263002 10.3389/fmolb.2020.569293 PMC7686857

[61] Nakamura Y , Tagawa K, Oka T, et al Ataxin-7 associates with microtubules and stabilizes the cytoskeletal network. Hum Mol Genet. 2012;21:1099–1110. 22100762 10.1093/hmg/ddr539 PMC3277310

[62] Yamamoto K , Seki T, Yamamoto H, et al Deregulation of the actin cytoskeleton and macropinocytosis in response to phorbol ester by the mutant protein kinase C gamma that causes spinocerebellar ataxia type 14. Front Physiol. 2014;5:126. 24744737 10.3389/fphys.2014.00126 PMC3978357

[63] Duncan EJ , Lariviere R, Bradshaw TY, et al Altered organization of the intermediate filament cytoskeleton and relocalization of proteostasis modulators in cells lacking the ataxia protein sacsin. Hum Mol Genet. 2017;26:3130–3143. 28535259 10.1093/hmg/ddx197 PMC5886247

[64] Wu XS , Subramanian S, Zhang Y, et al Presynaptic Kv3 channels are required for fast and slow endocytosis of synaptic vesicles. Neuron. 2021;109:938–946 e5. 33508244 10.1016/j.neuron.2021.01.006 PMC7979485

[65] Younis RM , Taylor RM, Beardsley PM, McClay JL. The ANKS1B gene and its associated phenotypes: Focus on CNS drug response. Pharmacogenomics. 2019;20:669–684. 31250731 10.2217/pgs-2019-0015 PMC6912848

[66] Stevens SR , van der Heijden ME, Ogawa Y, Lin T, Sillitoe RV, Rasband MN. Ankyrin-R links Kv3.3 to the spectrin cytoskeleton and is required for purkinje neuron survival. J Neurosci. 2022;42:2–15. 34785580 10.1523/JNEUROSCI.1132-21.2021 PMC8741159

[67] Fard MK , van der Meer F, Sanchez P, et al BCAS1 Expression defines a population of early myelinating oligodendrocytes in multiple sclerosis lesions. Sci Transl Med. 2017;9:eaam7816. 29212715 10.1126/scitranslmed.aam7816 PMC7116798

[68] Ishimoto T , Ninomiya K, Inoue R, Koike M, Uchiyama Y, Mori H. Mice lacking BCAS1, a novel myelin-associated protein, display hypomyelination, schizophrenia-like abnormal behaviors, and upregulation of inflammatory genes in the brain. Glia. 2017;65:727–739. 28230289 10.1002/glia.23129

[69] Schuster KH , DiFranco DM, Putka AF, et al Disease-associated oligodendrocyte signatures are spatiotemporally dysregulated in spinocerebellar ataxia type 3. Front Neurosci. 2023;17:1118429. 36875652 10.3389/fnins.2023.1118429 PMC9975394

[70] Schuster KH , Zalon AJ, Zhang H, et al Impaired oligodendrocyte maturation is an early feature in SCA3 disease pathogenesis. J Neurosci. 2022;42:1604–1617. 35042771 10.1523/JNEUROSCI.1954-20.2021 PMC8883861

[71] Ayhan F , Perez BA, Shorrock HK, et al SCA8 RAN polySer protein preferentially accumulates in white matter regions and is regulated by eIF3F. EMBO J. 2018;37:e99023. 30206144 10.15252/embj.201899023 PMC6166133

[72] Ozaki K , Irioka T, Uchihara T, et al Neuropathology of SCA34 showing widespread oligodendroglial pathology with vacuolar white matter degeneration: A case study. Acta Neuropathol Commun. 2021;9:172. 34689836 10.1186/s40478-021-01272-w PMC8543940

[73] Scherzed W , Brunt ER, Heinsen H, et al Pathoanatomy of cerebellar degeneration in spinocerebellar ataxia type 2 (SCA2) and type 3 (SCA3). Cerebellum. 2012;11:749–760. 22198871 10.1007/s12311-011-0340-8

[74] Costa MDC , Radzwion M, McLoughlin HS, et al In vivo molecular signatures of cerebellar pathology in spinocerebellar ataxia type 3. Mov Disord. 2020;35:1774–1786. 32621646 10.1002/mds.28140 PMC7572607

[75] Bailey CS , Moldenhauer HJ, Park SM, Keros S, Meredith AL. KCNMA1-linked Channelopathy. J Gen Physiol. 2019;151:1173–1189. 31427379 10.1085/jgp.201912457 PMC6785733

[76] Carvalho-de-Souza JL , Kubota T, Du X, Latorre R, Gomez CM, Bezanilla F. A missense mutation in the selectivity filter of BK affects the channel’s potassium conductance. Biophys J. 2016; 110:449a.

[77] Fodor AA , Aldrich RW. Convergent evolution of alternative splices at domain boundaries of the BK channel. Annu Rev Physiol. 2009;71:19–36. 18694345 10.1146/annurev.physiol.010908.163124

[78] Latorre R , Castillo K, Carrasquel-Ursulaez W, et al Molecular determinants of BK channel functional diversity and functioning. Physiol Rev. 2017;97:39–87. 27807200 10.1152/physrev.00001.2016

[79] Shams’ili S , Grefkens J, de Leeuw B, et al Paraneoplastic cerebellar degeneration associated with antineuronal antibodies: Analysis of 50 patients. Brain. 2003;126(Pt 6):1409–1418. 12764061 10.1093/brain/awg133

[80] Buckanovich RJ , Yang YY, Darnell RB. The onconeural antigen Nova-1 is a neuron-specific RNA-binding protein, the activity of which is inhibited by paraneoplastic antibodies. J Neurosci. 1996;16:1114–1122. 8558240 10.1523/JNEUROSCI.16-03-01114.1996 PMC6578795

[81] Meldolesi J . Alternative splicing by NOVA factors: From gene expression to cell physiology and pathology. Int J Mol Sci. 2020;21:3941. 32486302 10.3390/ijms21113941 PMC7312376

[82] Banez-Coronel M , Ranum LPW. Repeat-associated non-AUG (RAN) translation: Insights from pathology. Lab Invest. 2019;99:929–942. 30918326 10.1038/s41374-019-0241-x PMC7219275

[83] Olzscha H , Schermann SM, Woerner AC, et al Amyloid-like aggregates sequester numerous metastable proteins with essential cellular functions. Cell. 2011;144:67–78. 21215370 10.1016/j.cell.2010.11.050

[84] Wear MP , Kryndushkin D, O'Meally R, Sonnenberg JL, Cole RN, Shewmaker FP. Proteins with intrinsically disordered domains are preferentially recruited to polyglutamine aggregates. PLoS One. 2015;10:e0136362. 26317359 10.1371/journal.pone.0136362 PMC4552826

[85] Woerner AC , Frottin F, Hornburg D, et al Cytoplasmic protein aggregates interfere with nucleocytoplasmic transport of protein and RNA. Science. 2016;351:173–176. 26634439 10.1126/science.aad2033

[86] Banez-Coronel M , Ayhan F, Tarabochia AD, et al RAN Translation in Huntington disease. Neuron. 2015;88:667–677. 26590344 10.1016/j.neuron.2015.10.038 PMC4684947

[87] Muller IB , Meijers S, Kampstra P, et al Computational comparison of common event-based differential splicing tools: Practical considerations for laboratory researchers. BMC Bioinformatics. 2021;22:347. 34174808 10.1186/s12859-021-04263-9 PMC8236165

