# Supplementary material for: Widespread alternative splicing dysregulation occurs presymptomatically in CAG expansion spinocerebellar ataxias
Source: Brain. 2023 Sep 30;147(2):486–504. doi: 10.1093/brain/awad329 (PMC10834251; doi:10.1093/brain/awad329)
Supplement: awad329_Supplementary_Data [file awad329_supplementary_data.zip › brain-2023-01025-File010.pdf]

Expanded Table 1

Disease	Dataset	Reference	Mouse Model	Tissue Type	Age	Replicates	Library Selection	Sequencing method	Read length	Avg. Read Depth	Pass Threshold	Skipped Exon Events (SCA vs WT) p < 0.05; PSI > 10%	FDR < 0.1; PSI > 10%
SCA1	GSE122099	35	ATXN1[82Q] Tg	Cerebellum	5 weeks	3	Oligo dT	Paired	76	87537211	Yes	650	93
			ATXN1[82Q] Tg	Cerebellum	12 weeks	3	Oligo dT	Paired	76	92485001	Yes	759	136
			ATXN1[82Q] Tg	Inferior Olive	5 weeks	3	Oligo dT	Paired	76	99996263	Yes	610	55
			ATXN1[82Q] Tg	Inferior Olive	12 weeks	3	Oligo dT	Paired	76	97497000	Yes	609	69
			Atxn1 154Q/2Q	Cerebellum	5 weeks	3	Oligo dT	Paired	76	104290286	Yes	684	87
			Atxn1 154Q/2Q	Cerebellum	12 weeks	3	Oligo dT	Paired	76	115188043	Yes	797	154
			Atxn1 154Q/2Q	Inferior Olive	5 weeks	3	Oligo dT	Paired	76	87880555	Yes	780	147
			Atxn1 154Q/2Q	Inferior Olive	12 weeks	3	Oligo dT	Paired	76	99154063	Yes	757	101
SCA1	GSE114674	43	Atxn1 154Q/2Q, ASO treatment	Cerebellum	18 weeks	4	Oligo dT	Paired	126	119643770	Yes	1009	362
			Atxn1 154Q/2Q, ASO treatment	Pons	18 weeks	3	Oligo dT	Paired	126	82569603	Yes	1230	376
			Atxn1 154Q/2Q, ASO treatment	Medulla	18 weeks	3	Oligo dT	Paired	126	82934663	Yes	1217	330
			Atxn1 154Q/2Q, ASO treatment	Pons	28 weeks	4	Oligo dT	Paired	126	94023770	Yes	1157	462
			Atxn1 154Q/2Q, ASO treatment	Medulla	28 weeks	8, 7 ASO	Oligo dT	Paired	126	112181398	Yes	774	346
SCA1	GSE108256	47	Pcp2-ATXN1[82Q]	Cerebellum	12 weeks*	5	Oligo dT	Paired	126	114752434	Yes	1016	523
SCA1	GSE75778[§]	38	ATXN1[30Q] Tg	Cerebellum	5 weeks	3	Oligo dT	Paired	101	127380677	Yes	826	136
			ATXN1[82Q] Tg	Cerebellum	5 weeks	3	Oligo dT	Paired	101	130183849	Yes	886	208
			ATXN1[82Q] Tg	Cerebellum	12 weeks	3	Oligo dT	Paired	76	10182436	No	NA	NA
			ATXN1[82Q] Tg	Cerebellum	28 weeks	3	Oligo dT	Paired	76	9168448	No	NA	NA
SCA1	GSE114815[**]	44	Pcp2-ATXN1[82Q]	Cerebellum	9 weeks	5	Oligo dT	Paired	126	111934958	Yes	776	278
			ATXN1[82Q];CαM120A/M120A	Cerebellum	9 weeks	5	Oligo dT	Paired	126	118498910	Yes	1450	994
SCA1	GSE163885[†]	42	Atxn1 154Q/2Q	Cerebellum	6 weeks	4, 8 WT	cDNA	Paired	150	231979628	Yes	852	298
			Atxn1 154Q[S776A]/2Q	Cerebellum	6 weeks	4, 8 WT	cDNA	Paired	150	230593782	Yes	772	231
SCA2	PRJEB24319	45	ATXN2 Q127 Tg	Cerebellum	1 day	8	rRNA depletion	Single, stranded	50	22095459	No	NA	NA
			ATXN2 Q127 Tg	Cerebellum	3 weeks	8	rRNA depletion	Single, stranded	50	23312280	No	NA	NA
			ATXN2 Q127 Tg	Cerebellum	6 weeks	16	rRNA depletion	Single, stranded	50	21850856	No	NA	NA
SCA3	GSE107958	49	MJD84.2	Cerebellum	17.5 months	6, 8 WT	poly A selection	Paired	125	183848391	Yes	421	75
			MJD84.2	Cortex	17.5 months	6, 8 WT	poly A selection	Paired	125	175864178	Yes	460	84
			MJD84.2	Striatum	17.5 months	6, 8 WT	poly A selection	Paired	125	193332930	Yes	634	167
			MJD84.2	Brainstem	17.5 months	6, 8 WT	poly A selection	Paired	125	68227029	Yes	463	127
SCA3	GSE117605[¶]	46	YAC15Q hemi	Pons	22-24 weeks	4, 6 WT	cDNA	Paired	31-101	51185663	Yes	583	101
			YAC84Q hemi	Pons	22-24 weeks	4, 6 WT	cDNA	Paired	34-101	56641310	Yes	645	157
			KI-het	Pons	22-24 weeks	4, 6 WT	cDNA	Paired	34-101	4714289	No	NA	NA
			KI-hom	Pons	22-24 weeks	4, 6 WT	cDNA	Paired	31-101	59753664	Yes	700	165
SCA3	GSE145613	37	304/304Q	Cerebellum	2 months	5	polyA enrichment	Paired, stranded	101	47636024	Yes	970	547
			304/304Q	Cerebellum	12 months	5	polyA enrichment	Paired, stranded	101	36060638	Yes	1154	698
SCA3	GSE178367	39	YAC15Q (no WT)	Cerebellum	18 months	3	polyA selection	Paired, stranded	30-151	104531443	Yes	NA	NA
			YAC84Q, IGF1 treatment	Cerebellum	18 months	3	polyA selection	Paired, stranded	30-151	58606848	Yes	1249	445
SCA7	GSE138527[#]	41	140Q/5Q	Cerebellum	40 weeks	3	poly-T oligo-purification	Single, stranded	50	43319128	Yes	885	248
SCA7	GSE139090	48	SCA7 92Q	Cerebellum	12 weeks	3	cDNA	Single	49	33206832	No	NA	NA
			SCA7 92Q	Cerebellum	29 weeks	3	cDNA	Single	49	34817416	No	NA	NA
SCA17	GSE145067	40	TBP-105Q	Cerebellum	3 months	4	cDNA	Single	101	11548250	No	NA	NA
			TBP-105Q	Striatum	3 months	3	cDNA	Single	101	10299866	No	NA	NA
			TBP-105Q	Prefrontal	3 months	3	cDNA	Single	101	9952160	No	NA	NA

Supplementary Table 1. Start and end coordinates of skipped exons

Start and end coordinates from mouse genome GRCm38/mm10 of skipped exons shown in Figures 5, 6, and Supplementary Fig. 7, 9 and 10.

Gene	Exon number	Chromosome	Start coordinate	End coordinate
Trpc3	9	3	36634374	36634458
Kcnma1	23b	14	23336039	23336120
Anks1b	5	10	90914695	90914767
Bcas1	9	2	170370449	170370491
Bcas1	10	2	170366363	170366528
Robo1	18	16	72993666	72993693
Pex5l	2	3	33081975	33082229
Tmem234	3	4	129601406	129601625
Gabpb2	3	3	95208839	95208936
Adgrb2	4	4	130006751	130006916
Itpa	4	2	130672059	130672338
Mier1	3	4	103118238	103118315
Kif21a	30	15	90949005	90949044
Itpr1	41	6	108431460	108431496
Camk2a	14	18	60969010	60969043

Supplementary Tables 2-5, 8, 9 and 11 are multi-tab excel workbooks that can be found as individual files in the supplementary material.

Supplementary Table 6. Number and percentage of splicing events by category

Disease	Dataset	Mouse Model	Tissue Type	Age	A3SS Number	A3SS %	A5SS Number	A5SS %	MXE Number	MXE %	RI Number	RI %	SE Number	SE %
SCA1	GSE122099	ATXN1[82Q] Tg	Cerebellum	5 weeks	20	11.76	21	12.35	12	7.06	24	14.12	93	54.71
		ATXN1[82Q] Tg	Cerebellum	12 weeks	13	6.28	21	10.14	18	8.70	19	9.18	136	65.70
		ATXN1[82Q] Tg	Inferior Olive	5 weeks	14	11.76	23	19.33	1	0.84	26	21.85	55	46.22
		ATXN1[82Q] Tg	Inferior Olive	12 weeks	22	17.74	18	14.52	4	3.23	11	8.87	69	55.65
		Atxn1 154Q/2Q	Cerebellum	5 weeks	17	12.23	5	3.60	6	4.32	24	17.27	87	62.59
		Atxn1 154Q/2Q	Cerebellum	12 weeks	22	9.65	15	6.58	12	5.26	25	10.96	154	67.54
		Atxn1 154Q/2Q	Inferior Olive	5 weeks	49	11.639	24	5.7007	161	38.242	40	9.5012	147	34.917
		Atxn1 154Q/2Q	Inferior Olive	12 weeks	22	13.75	7	4.38	4	2.50	26	16.25	101	63.13
SCA1	GSE114674	Atxn1 154Q/2Q	Cerebellum	18 weeks	48	8.89	39	7.22	33	6.11	58	10.74	362	67.04
		Atxn1 154Q/2Q	Pons	18 weeks	89	11.097	59	7.3566	209	26.06	69	8.6035	376	46.883
		Atxn1 154Q/2Q	Medulla	18 weeks	87	15.647	48	8.6331	7	1.259	84	15.108	330	59.353
		Atxn1 154Q/2Q	Pons	28 weeks	60	8.8889	54	8	39	5.7778	60	8.8889	462	68.444
		Atxn1 154Q/2Q	Medulla	28 weeks	50	9.5969	38	7.2937	39	7.4856	48	9.2131	346	66.411
SCA1	GSE108256	Pcp2-ATXN1[82Q]	Cerebellum	12 weeks	71	9.15	63	8.12	59	7.60	60	7.73	523	67.40
SCA1	GSE75778	ATXN1[82Q] Tg	Cerebellum	5 weeks	33	10.25	31	9.63	13	4.04	37	11.49	208	64.60
SCA1	GSE114815	Pcp2-ATXN1[82Q]	Cerebellum	9 weeks	21	5.5556	26	6.8783	29	7.672	24	6.3492	278	73.545
SCA1	GSE163885	Atxn1 154Q/2Q	Cerebellum	6 weeks	57	8.1545	37	5.2933	32	4.578	275	39.342	298	42.632
SCA3	GSE107958	MJD84.2	Cerebellum	17.5 months	20	16.807	6	5.042	8	6.7227	10	8.4034	75	63.025
		MJD84.2	Cortex	17.5 months	8	6.90	12	10.34	5	4.31	7	6.03	84	72.41
		MJD84.2	Striatum	17.5 months	28	10.728	6	2.2989	35	13.41	25	9.5785	167	63.985
		MJD84.2	Brainstem	17.5 months	11	5.5	6	3	46	23	10	5	127	63.5
SCA3	GSE117605	YAC84Q hemi	Pons	22-24 weeks	31	12.653	19	7.7551	6	2.449	32	13.061	157	64.082
		KI-hom	Pons	22-24 weeks	20	7.58	22	8.33	10	3.79	47	17.80	165	62.50
SCA3	GSE145613	304/304Q	Cerebellum	2 months	106	11.35	84	8.99	79	8.46	118	12.63	547	58.57
		304/304Q	Cerebellum	12 months	137	12.00	102	8.93	62	5.43	143	12.52	698	61.12
SCA7	GSE138527	140Q/5Q	Cerebellum	40 weeks	47	12.082	50	12.853	16	4.1131	28	7.1979	248	63.753

Supplementary Table 7. Genes corresponding to the gene ontology term 'splicing factor NOVA regulated synaptic proteins'

Genes corresponding to the gene ontology term 'splicing factor NOVA regulated synaptic proteins' identified as enriched using metascape in Figures 1F, 3C and 4B.

Figure 1F	Figure 3C	Figure 4B
Ank3	Ank3	Ank3
Epb41l1	Cask	Kcnma1
Stx2	Epb41l2	Prkcz
Grin1	Grik2	
Kcnma1	Grin2b	
Kcnq2	Kcnq2	
Prkcz	Map4	
Ntng1	Prkcz	
	Clasp1	
	Epb41	
	Dlg2	
	Shank3	
	Ptprd	
	Ppfibp1	

Supplementary Table 10. Number of presymptomatic differentially expressed genes identified at different $\log_2$FoldChange thresholds DEGs found refers to the number of differentially expressed genes identified in this study. DEGs reported, threshold and method refer to the number of differentially expressed genes reported in the original dataset publication at the specified threshold and method used in each publication[35,37,42].

	DEGs Found (STAR, DESeq2)						
**Presymptomatic Datasets**	**log2FC >|1.5| padj < 0.05**	**log2FC >|1.0| padj < 0.05**	**log2FC >|0.5|**	**padj < 0.05**	**DEGs Reported**	**Threshold**	**Method**
GSE75778 SCA1 82Q 5wks	113	199	450	1388	~1000	q ≤ 0•05	TopHat2, Cuffdiff2, Cuffquant, Cuffnorm
GSE122099 SCA1 82Q 5wks	26	78	241	648	402	FDR pvalue < 0•05	TopHat2, Cuffnorm, Cufflinks
GSE122099 SCA1 154Q 5wks	1	4	23	34	25	FDR pvalue < 0•05	TopHat2, Cuffnorm, Cufflinks
GSE163885 SCA1 154Q 6wks	7	28	150	520	118	padj < 0•01, log2FC > |0•5|	STAR, DESeq2
GSE145613 SCA3 304Q/304Q 2mo	1	1	9	22	6	FDR pvalue ≤ 0•05, log2FC > |0•5|	STAR, DESeq2, Rsubread
GSE122099 SCA1 82Q 5wks IO	0	7	25	62	148	FDR pvalue < 0•05	TopHat2, Cuffnorm, Cufflinks
GSE122099 SCA1 154Q 5wks IO	21	31	59	73	143	FDR pvalue < 0•05	TopHat2, Cuffnorm, Cufflinks

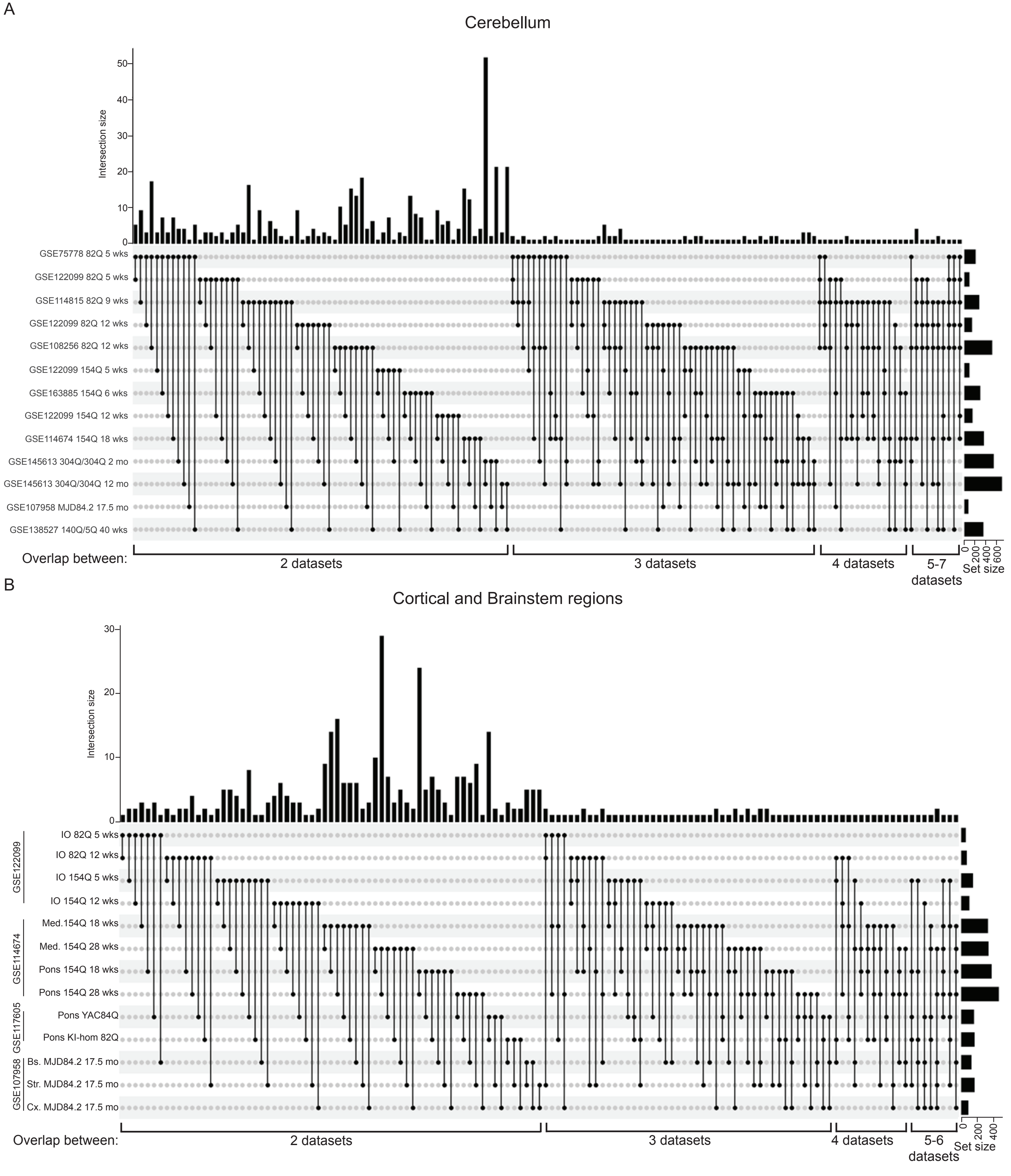

Supplementary Fig. 1. Pairwise comparisons of shared skipped exon events

A-B Upset plots showing overlap of significant skipped exon events across cerebellar (**A**) and cortical and brainstem region (**B**) datasets, FDR<0.1, DPSI>10%.

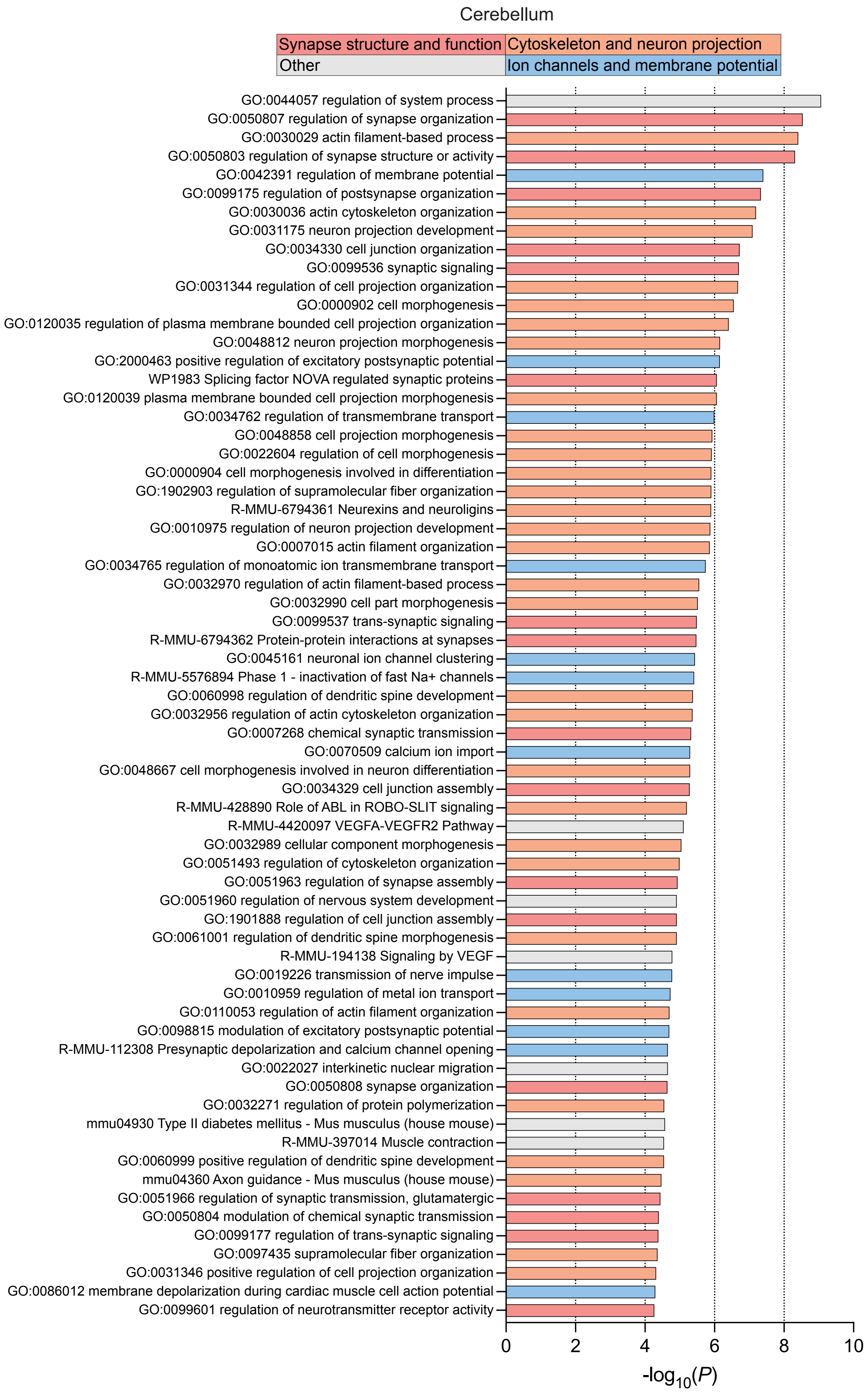

Cerebellum
Synapse structure and function
Cytoskeleton and neuron projection
Other
Ion channels and membrane potential
GO:0044057 regulation of system process
GO:0050807 regulation of synapse organization
GO:0030029 actin filament-based process
GO:0050803 regulation of synapse structure or activity
GO:0042391 regulation of membrane potential
GO:0099175 regulation of postsynapse organization
GO:0030036 actin cytoskeleton organization
GO:0031175 neuron projection development
GO:0034330 cell junction organization
GO:0099536 synaptic signaling
GO:0031344 regulation of cell projection organization
GO:0000902 cell morphogenesis
GO:0120035 regulation of plasma membrane bounded cell projection organization
GO:0048812 neuron projection morphogenesis
GO:2000463 positive regulation of excitatory postsynaptic potential
WP1983 Splicing factor NOVA regulated synaptic proteins
GO:0120039 plasma membrane bounded cell projection morphogenesis
GO:0034762 regulation of transmembrane transport
GO:0048858 cell projection morphogenesis
GO:0022604 regulation of cell morphogenesis
GO:0000904 cell morphogenesis involved in differentiation
GO:1902903 regulation of supramolecular fiber organization
R-MMU-6794361 Neurexins and neuroligins
GO:0010975 regulation of neuron projection development
GO:0007015 actin filament organization
GO:0034765 regulation of monoatomic ion transmembrane transport
GO:0032970 regulation of actin filament-based process
GO:0032990 cell part morphogenesis
GO:0099537 trans-synaptic signaling
R-MMU-6794362 Protein-protein interactions at synapses
GO:0045161 neuronal ion channel clustering
R-MMU-5576894 Phase 1 - inactivation of fast Na+ channels
GO:0060998 regulation of dendritic spine development
GO:0032956 regulation of actin cytoskeleton organization
GO:0007268 chemical synaptic transmission
GO:0070509 calcium ion import
GO:0048667 cell morphogenesis involved in neuron differentiation
GO:0034329 cell junction assembly
R-MMU-428890 Role of ABL in ROBO-SLIT signaling
R-MMU-4420097 VEGFA-VEGFR2 Pathway
GO:0032989 cellular component morphogenesis
GO:0051493 regulation of cytoskeleton organization
GO:0051963 regulation of synapse assembly
GO:0051960 regulation of nervous system development
GO:1901888 regulation of cell junction assembly
GO:0061001 regulation of dendritic spine morphogenesis
R-MMU-194138 Signaling by VEGF
GO:0019226 transmission of nerve impulse
GO:0010959 regulation of metal ion transport
GO:0110053 regulation of actin filament organization
GO:0098815 modulation of excitatory postsynaptic potential
R-MMU-112308 Presynaptic depolarization and calcium channel opening
GO:0022027 interkinetic nuclear migration
GO:0050808 synapse organization
GO:0032271 regulation of protein polymerization
mmu04930 Type II diabetes mellitus - Mus musculus (house mouse)
R-MMU-397014 Muscle contraction
GO:0060999 positive regulation of dendritic spine development
mmu04360 Axon guidance - Mus musculus (house mouse)
GO:0051966 regulation of synaptic transmission, glutamatergic
GO:0050804 modulation of chemical synaptic transmission
GO:0099177 regulation of trans-synaptic signaling
GO:0097435 supramolecular fiber organization
GO:0031346 positive regulation of cell projection organization
GO:0086012 membrane depolarization during cardiac muscle cell action potential
GO:0099601 regulation of neurotransmitter receptor activity
0
2
4
6
8
10
$-\log_{10}(P)$

Supplementary Fig. 2. Gene ontology enrichment member terms for cerebellar analyses

Gene ontology enrichment member terms for Metascape analysis of skipped exon events significantly dysregulated in two or more cerebellar datasets; broad functional categories of terms are indicated by bar colour; SE events: FDR<0.1, DPSI>10%; see Figure 1F for enrichment of summary terms.

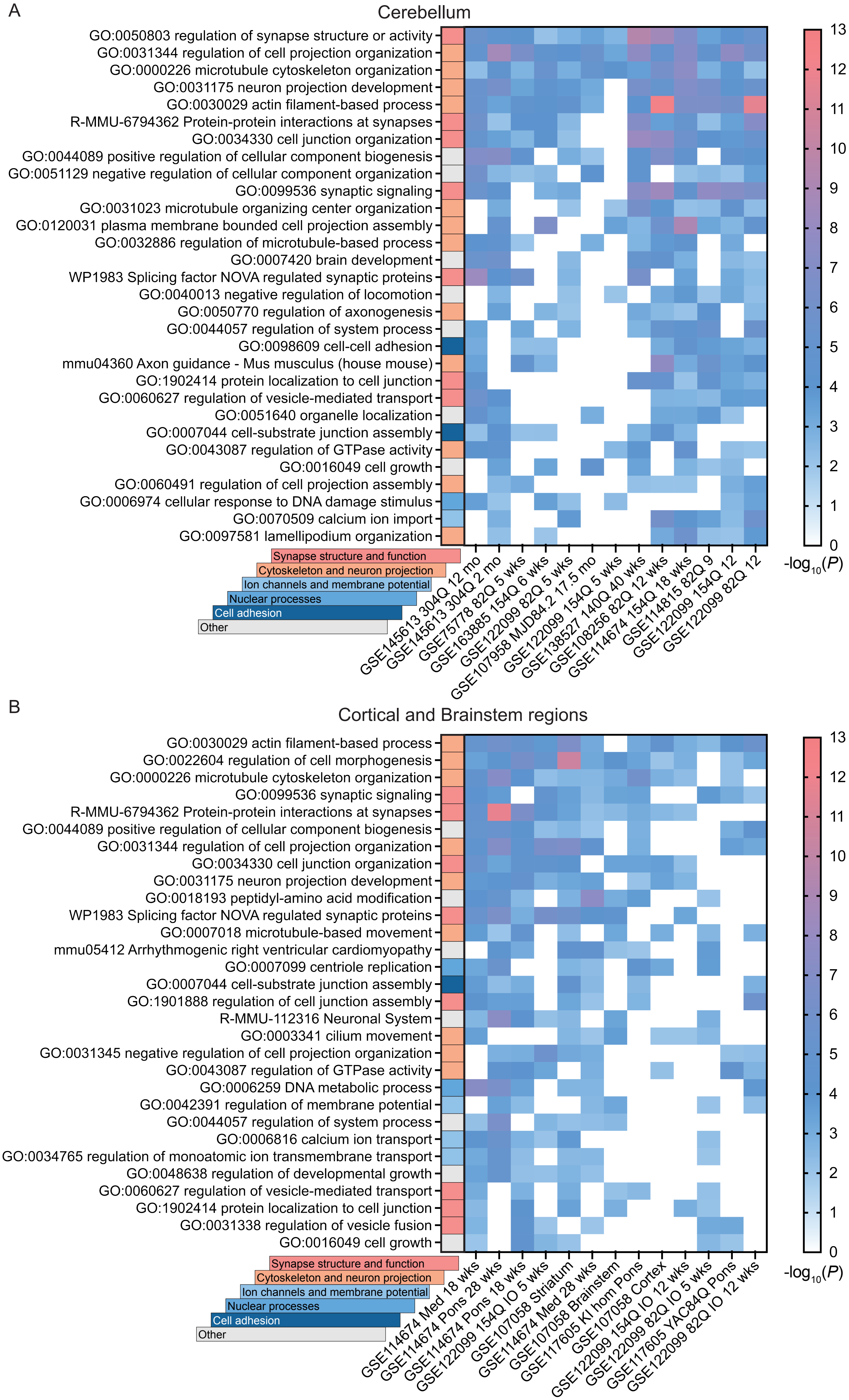
A
Cerebellum
GO:0050803 regulation of synapse structure or activity
GO:0031344 regulation of cell projection organization
GO:0000226 microtubule cytoskeleton organization
GO:0031175 neuron projection development
GO:0030029 actin filament-based process
R-MMU-6794362 Protein-protein interactions at synapses
GO:0034330 cell junction organization
GO:0044089 positive regulation of cellular component biogenesis
GO:0051129 negative regulation of cellular component organization
GO:0099536 synaptic signaling
GO:0031023 microtubule organizing center organization
GO:0120031 plasma membrane bounded cell projection assembly
GO:0032886 regulation of microtubule-based process
GO:0007420 brain development
WP1983 Splicing factor NOVA regulated synaptic proteins
GO:0040013 negative regulation of locomotion
GO:0050770 regulation of axonogenesis
GO:0044057 regulation of system process
GO:0098609 cell-cell adhesion
mmu04360 Axon guidance - Mus musculus (house mouse)
GO:1902414 protein localization to cell junction
GO:0060627 regulation of vesicle-mediated transport
GO:0051640 organelle localization
GO:0007044 cell-substrate junction assembly
GO:0043087 regulation of GTPase activity
GO:0016049 cell growth
GO:0060491 regulation of cell projection assembly
GO:0006974 cellular response to DNA damage stimulus
GO:0070509 calcium ion import
GO:0097581 lamellipodium organization
Synapse structure and function
Cytoskeleton and neuron projection
Ion channels and membrane potential
Nuclear processes
Cell adhesion
Other
GSE145613 304Q 12 mo
GSE145613 304Q 2 mo
GSE75778 82Q 5 wks
GSE163885 154Q 6 wks
GSE122099 82Q 5 wks
GSE107958 MJD84.2 17.5 mo
GSE122099 154Q 5 wks
GSE138527 140Q 40 wks
GSE108256 82Q 12 wks
GSE114674 154Q 18 wks
GSE114815 82Q 9
GSE122099 154Q 12
GSE122099 82Q 12
13
12
11
10
9
8
7
6
5
4
3
2
1
0
-log10(P)
B
Cortical and Brainstem regions
GO:0030029 actin filament-based process
GO:0022604 regulation of cell morphogenesis
GO:0000226 microtubule cytoskeleton organization
GO:0099536 synaptic signaling
R-MMU-6794362 Protein-protein interactions at synapses
GO:0044089 positive regulation of cellular component biogenesis
GO:0031344 regulation of cell projection organization
GO:0034330 cell junction organization
GO:0031175 neuron projection development
GO:0018193 peptidyl-amino acid modification
WP1983 Splicing factor NOVA regulated synaptic proteins
GO:0007018 microtubule-based movement
mmu05412 Arrhythmogenic right ventricular cardiomyopathy
GO:0007099 centriole replication
GO:0007044 cell-substrate junction assembly
GO:1901888 regulation of cell junction assembly
R-MMU-112316 Neuronal System
GO:0003341 cilium movement
GO:0031345 negative regulation of cell projection organization
GO:0043087 regulation of GTPase activity
GO:0006259 DNA metabolic process
GO:0042391 regulation of membrane potential
GO:0044057 regulation of system process
GO:0006816 calcium ion transport
GO:0034765 regulation of monoatomic ion transmembrane transport
GO:0048638 regulation of developmental growth
GO:0060627 regulation of vesicle-mediated transport
GO:1902414 protein localization to cell junction
GO:0031338 regulation of vesicle fusion
GO:0016049 cell growth
Synapse structure and function
Cytoskeleton and neuron projection
Ion channels and membrane potential
Nuclear processes
Cell adhesion
Other
GSE114674 Med 18 wks
GSE114674 Pons 28 wks
GSE114674 Pons 18 wks
GSE122099 154Q IO 5 wks
GSE107058 Striatum
GSE114674 Med 28 wks
GSE107058 Brainstem
GSE117605 KI hom Pons
GSE107058 Cortex
GSE122099 154Q IO 12 wks
GSE122099 82Q IO 5 wks
GSE117605 YAC84Q Pons
GSE122099 82Q IO 12 wks
13
12
11
10
9
8
7
6
5
4
3
2
1
0
-log10(P)

Supplementary Fig. 3. Gene ontology enrichment heatmaps

A Heatmap showing enrichment of gene ontology terms in each cerebellar dataset based on SE events $P<0.05$, ΔPSI>10%.

B Heatmap showing enrichment of gene ontology terms in each cortical and brainstem region dataset based on SE events $P<0.05$, ΔPSI>10%. **A, B** broad functional categories are indicated by the left most column of coloured boxes.

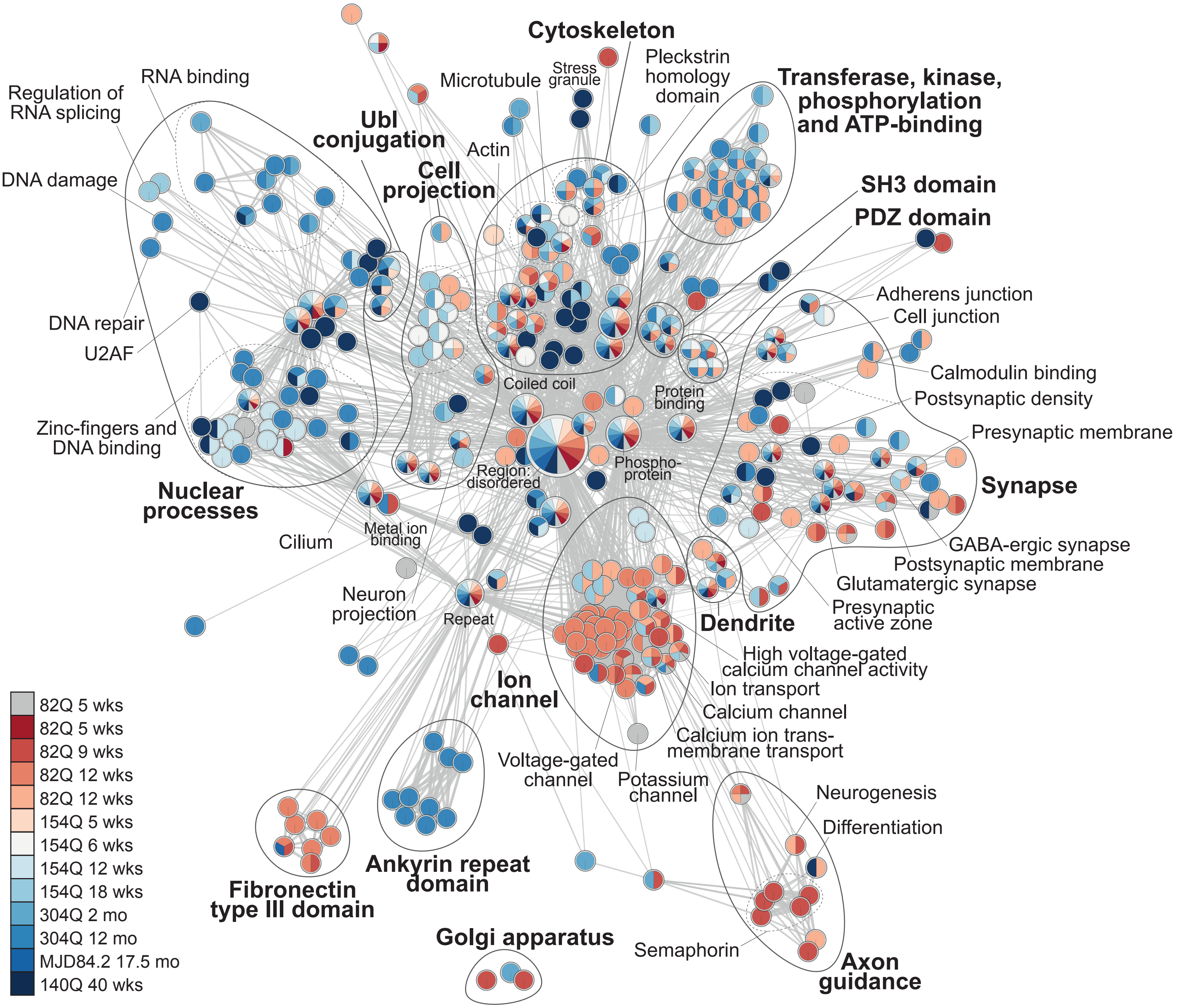

Supplementary Fig. 4. Functional annotation clustering of significantly misregulated skipped exon events for cerebellar datasets with detailed annotations

See Figure 1G.
