# Supplementary material for: Widespread alternative splicing dysregulation occurs presymptomatically in CAG expansion spinocerebellar ataxias
Source: Brain. 2023 Sep 30;147(2):486–504. doi: 10.1093/brain/awad329 (PMC10834251; doi:10.1093/brain/awad329)
Supplement: awad329_Supplementary_Data [file awad329_supplementary_data.zip › brain-2023-01025-File011.pdf]

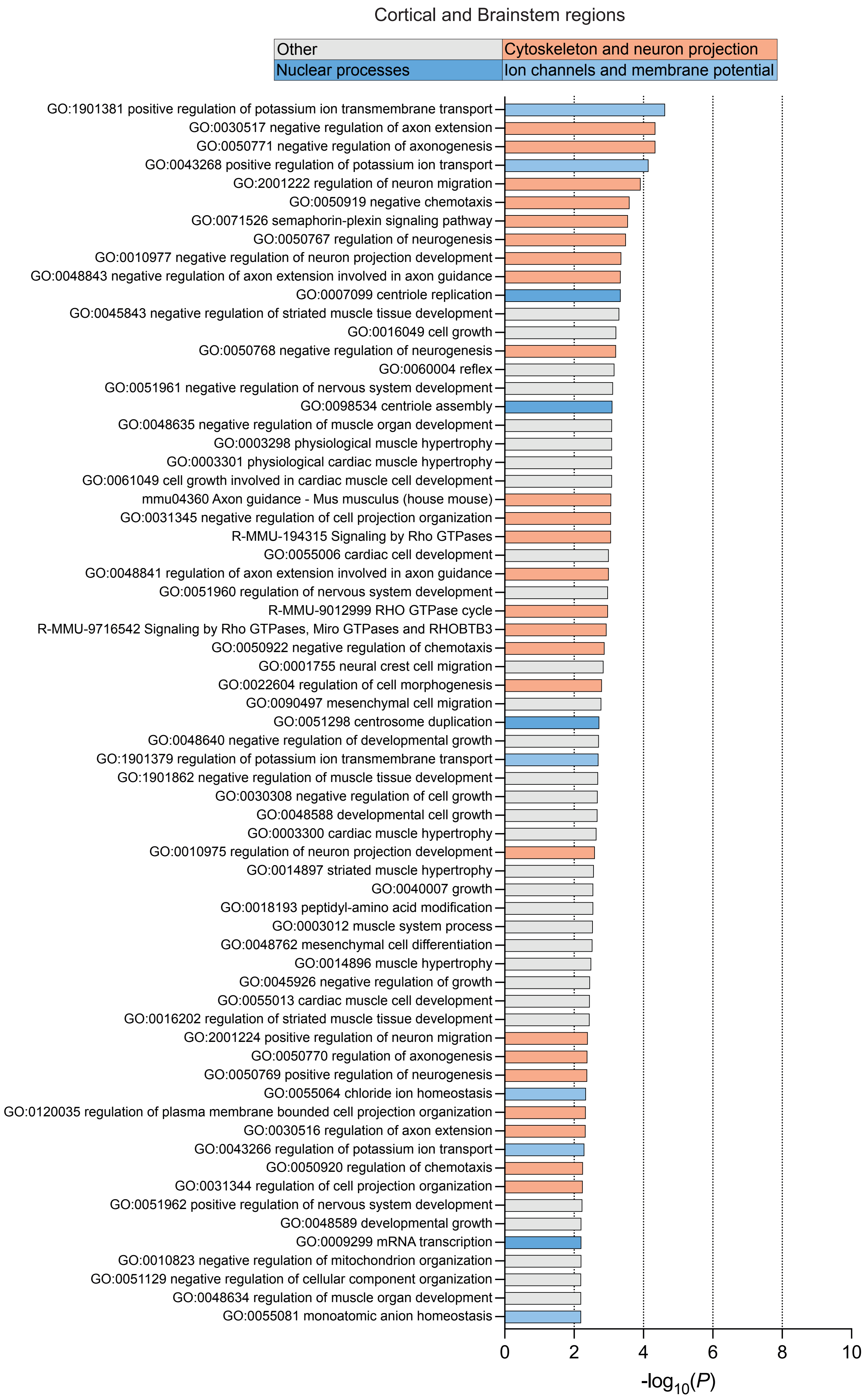

Cortical and Brainstem regions
Other
Cytoskeleton and neuron projection
Nuclear processes
Ion channels and membrane potential
GO:1901381 positive regulation of potassium ion transmembrane transport
GO:0030517 negative regulation of axon extension
GO:0050771 negative regulation of axonogenesis
GO:0043268 positive regulation of potassium ion transport
GO:2001222 regulation of neuron migration
GO:0050919 negative chemotaxis
GO:0071526 semaphorin-plexin signaling pathway
GO:0050767 regulation of neurogenesis
GO:0010977 negative regulation of neuron projection development
GO:0048843 negative regulation of axon extension involved in axon guidance
GO:0007099 centriole replication
GO:0045843 negative regulation of striated muscle tissue development
GO:0016049 cell growth
GO:0050768 negative regulation of neurogenesis
GO:0060004 reflex
GO:0051961 negative regulation of nervous system development
GO:0098534 centriole assembly
GO:0048635 negative regulation of muscle organ development
GO:0003298 physiological muscle hypertrophy
GO:0003301 physiological cardiac muscle hypertrophy
GO:0061049 cell growth involved in cardiac muscle cell development
mmu04360 Axon guidance - Mus musculus (house mouse)
GO:0031345 negative regulation of cell projection organization
R-MMU-194315 Signaling by Rho GTPases
GO:0055006 cardiac cell development
GO:0048841 regulation of axon extension involved in axon guidance
GO:0051960 regulation of nervous system development
R-MMU-9012999 RHO GTPase cycle
R-MMU-9716542 Signaling by Rho GTPases, Miro GTPases and RHOBTB3
GO:0050922 negative regulation of chemotaxis
GO:0001755 neural crest cell migration
GO:0022604 regulation of cell morphogenesis
GO:0090497 mesenchymal cell migration
GO:0051298 centrosome duplication
GO:0048640 negative regulation of developmental growth
GO:1901379 regulation of potassium ion transmembrane transport
GO:1901862 negative regulation of muscle tissue development
GO:0030308 negative regulation of cell growth
GO:0048588 developmental cell growth
GO:0003300 cardiac muscle hypertrophy
GO:0010975 regulation of neuron projection development
GO:0014897 striated muscle hypertrophy
GO:0040007 growth
GO:0018193 peptidyl-amino acid modification
GO:0003012 muscle system process
GO:0048762 mesenchymal cell differentiation
GO:0014896 muscle hypertrophy
GO:0045926 negative regulation of growth
GO:0055013 cardiac muscle cell development
GO:0016202 regulation of striated muscle tissue development
GO:2001224 positive regulation of neuron migration
GO:0050770 regulation of axonogenesis
GO:0050769 positive regulation of neurogenesis
GO:0055064 chloride ion homeostasis
GO:0120035 regulation of plasma membrane bounded cell projection organization
GO:0030516 regulation of axon extension
GO:0043266 regulation of potassium ion transport
GO:0050920 regulation of chemotaxis
GO:0031344 regulation of cell projection organization
GO:0051962 positive regulation of nervous system development
GO:0048589 developmental growth
GO:0009299 mRNA transcription
GO:0010823 negative regulation of mitochondrion organization
GO:0051129 negative regulation of cellular component organization
GO:0048634 regulation of muscle organ development
GO:0055081 monoatomic anion homeostasis
0
2
4
6
8
10
-log10(P)

Supplementary Fig. 5. Gene ontology enrichment member terms for cortical and brainstem region analyses

Gene ontology enrichment member terms for Metascape analysis of skipped exon events significantly dysregulated in two or more cortical or brainstem region datasets; broad functional categories of terms are indicated by bar colour; SE events: FDR<0.1, ΔPSI>10%; see Figure 2F for enrichment of summary terms.

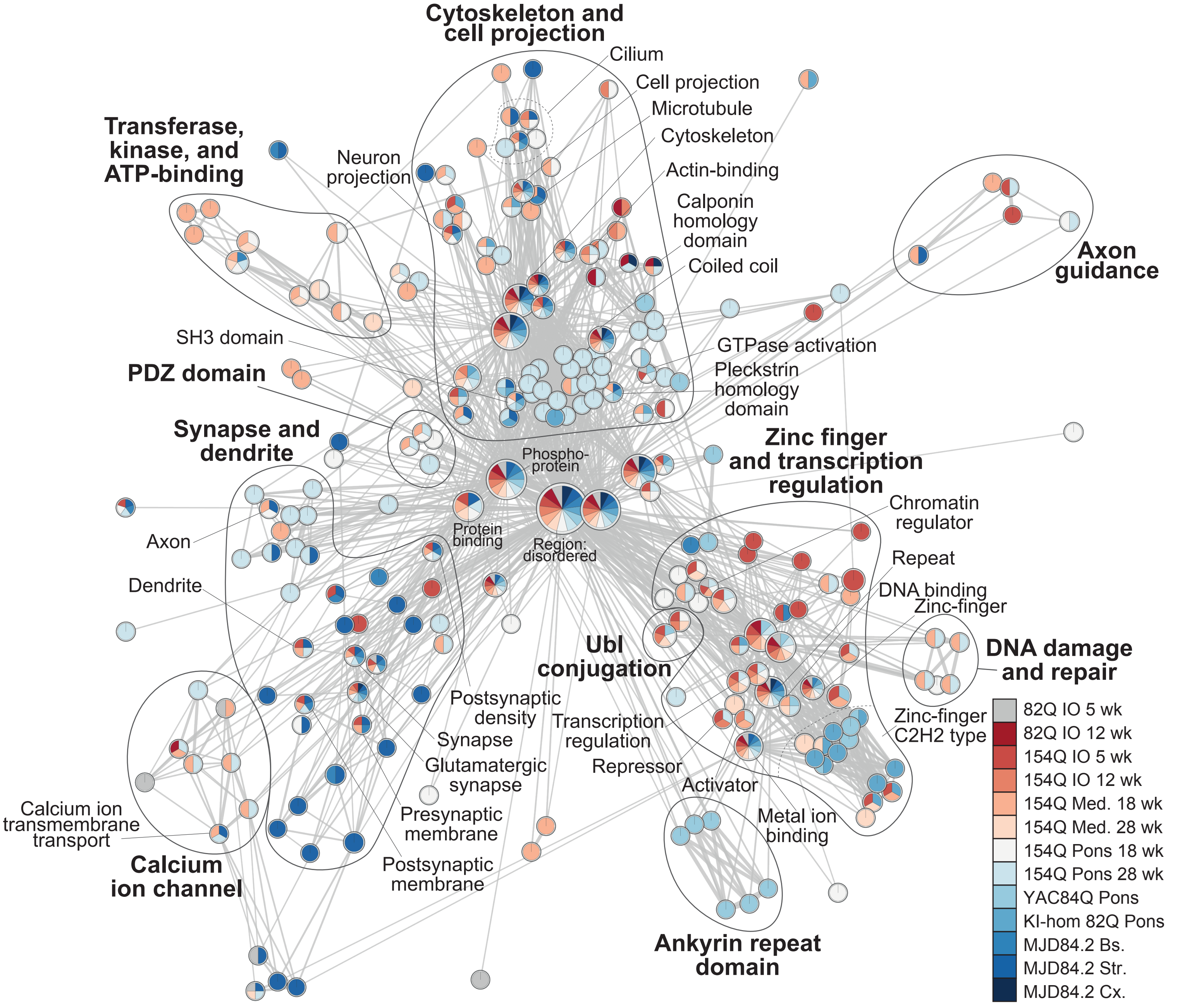

Supplementary Fig. 6. Functional annotation clustering of significantly misregulated skipped exon events for cortical and brainstem region datasets with detailed annotations

See Figure 2G.

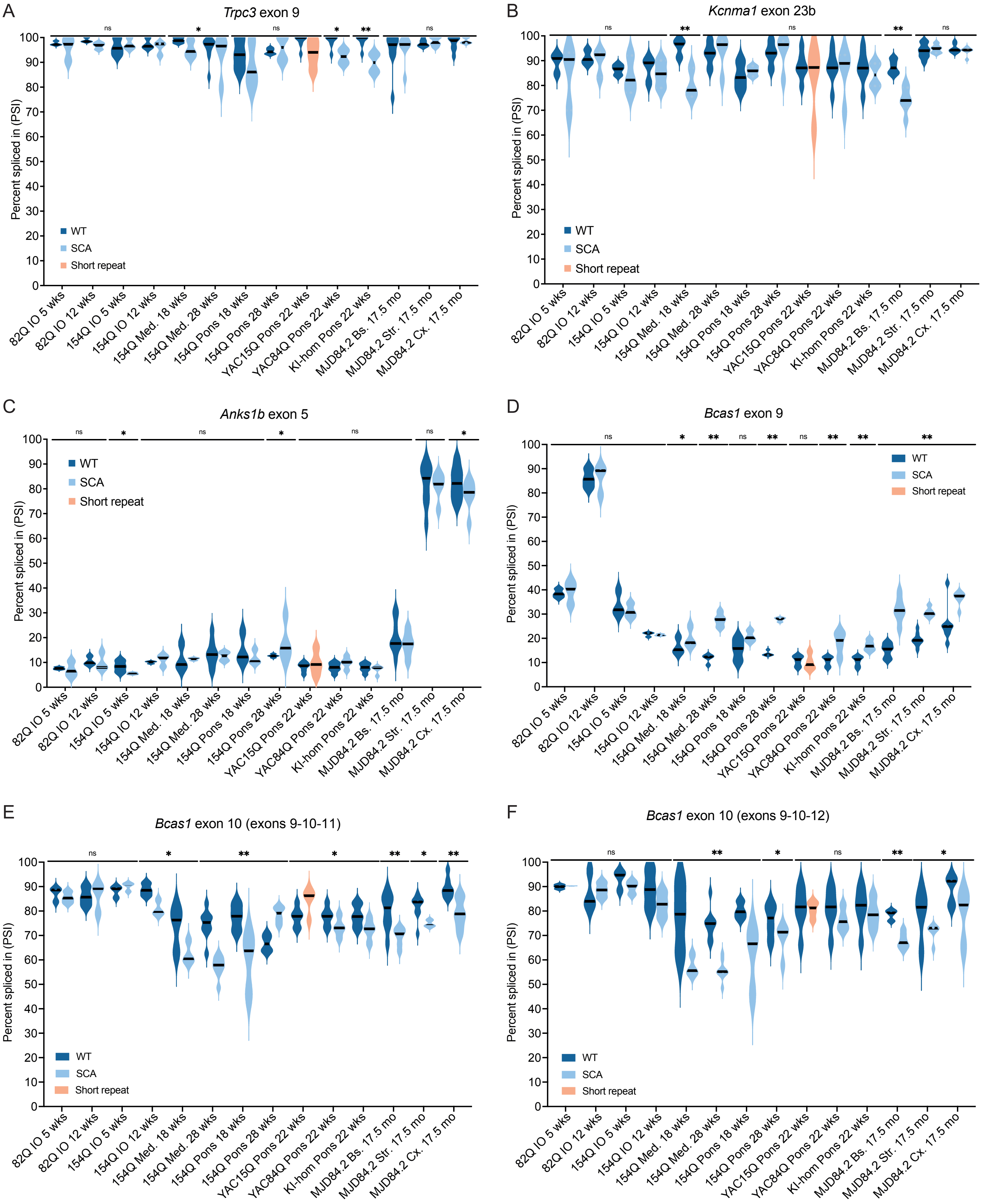

A
Trpc3 exon 9
B
Kcnma1 exon 23b
C
Anks1b exon 5
D
Bcas1 exon 9
E
Bcas1 exon 10 (exons 9-10-11)
F
Bcas1 exon 10 (exons 9-10-12)
Percent spliced in (PSI)
WT
SCA
Short repeat
ns
*
**
82Q IO 5 wks
82Q IO 12 wks
154Q IO 5 wks
154Q IO 12 wks
154Q Med. 18 wks
154Q Med. 28 wks
154Q Pons 18 wks
154Q Pons 28 wks
YAC15Q Pons 22 wks
YAC84Q Pons 22 wks
KI-hom Pons 22 wks
MJD84.2 Bs. 17.5 mo
MJD84.2 Str. 17.5 mo
MJD84.2 Cx. 17.5 mo

Supplementary Fig. 7. Alternative splicing of key CAG expansion SCA skipped exon events in cortical and brainstem regions

A-C *Trpc3* exon 9, *Kcnma1* exon 23b and *Anks1b* exon 5, respectively, show different splicing profiles in brainstem and cortical regions compared to cerebellum and show little missplicing in brainstem and cortical regions; ns – not significant, **P*<0.05, **FDR<0.1; see Figure 5.

D-F Dysregulation of *Bcas1* exon 9 and exon 10 alternative splicing occurs across brainstem regions from SCA1 and SCA3 mouse models; **E** *Bcas1* exon 10 in the context of exon 9, 10, 11; **F** *Bcas1* exon 10 in the context of exon 9, 10, 12. ns – not significant, **P*<0.05, **FDR<0.1.

A-F Datasets shown in the same order as Figure 2A; line indicates median.

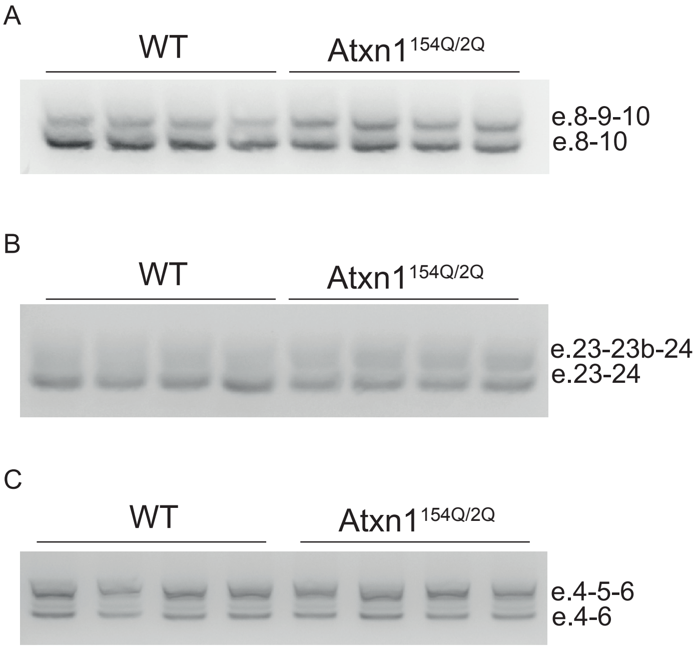

Supplementary Figure 8. Example agarose gel electrophoresis for validated alternative splicing events

A-C Representative images for (**A**) *Trpc3* exon 9, (**B**) *Kcnma1* exon 23b and (**C**) *Anks1b* exon 5 for 12-week-old WT and Atxn11154Q/2Q mice (n=4) indicating inclusion and exclusion events, e. - exon.

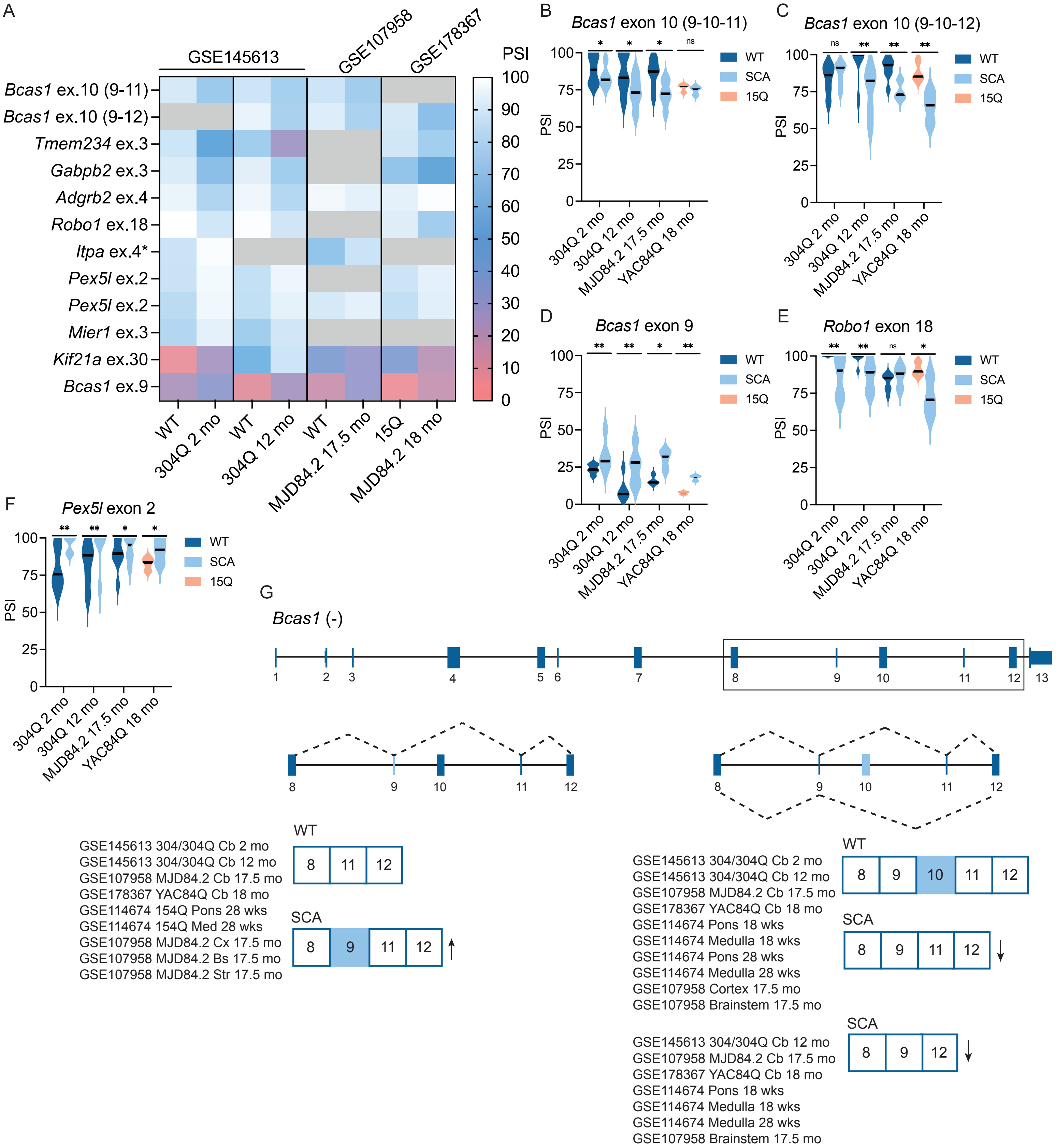
A
GSE145613
GSE107958
GSE178367
PSI
Bcas1 ex.10 (9-11)
Bcas1 ex.10 (9-12)
Tmem234 ex.3
Gabpb2 ex.3
Adgrb2 ex.4
Robo1 ex.18
Itpa ex.4*
Pex5l ex.2
Pex5l ex.2
Mier1 ex.3
Kif21a ex.30
Bcas1 ex.9
WT
304Q 2 mo
WT
304Q 12 mo
WT
MJD84.2 17.5 mo
15Q
MJD84.2 18 mo
B
Bcas1 exon 10 (9-10-11)
C
Bcas1 exon 10 (9-10-12)
D
Bcas1 exon 9
E
Robo1 exon 18
F
Pex5l exon 2
PSI
WT
SCA
15Q
304Q 2 mo
304Q 12 mo
MJD84.2 17.5 mo
YAC84Q 18 mo
ns
G
Bcas1 (-)
WT
SCA
GSE145613 304/304Q Cb 2 mo
GSE145613 304/304Q Cb 12 mo
GSE107958 MJD84.2 Cb 17.5 mo
GSE178367 YAC84Q Cb 18 mo
GSE114674 154Q Pons 28 wks
GSE114674 154Q Med 28 wks
GSE107958 MJD84.2 Cx 17.5 mo
GSE107958 MJD84.2 Bs 17.5 mo
GSE107958 MJD84.2 Str 17.5 mo
GSE145613 304/304Q Cb 2 mo
GSE145613 304/304Q Cb 12 mo
GSE107958 MJD84.2 Cb 17.5 mo
GSE178367 YAC84Q Cb 18 mo
GSE114674 Pons 18 wks
GSE114674 Medulla 18 wks
GSE114674 Pons 28 wks
GSE114674 Medulla 28 wks
GSE107958 Cortex 17.5 mo
GSE107958 Brainstem 17.5 mo
GSE145613 304/304Q Cb 12 mo
GSE107958 MJD84.2 Cb 17.5 mo
GSE178367 YAC84Q Cb 18 mo
GSE114674 Pons 18 wks
GSE114674 Medulla 18 wks
GSE114674 Medulla 28 wks
GSE107958 Brainstem 17.5 mo

Supplementary Fig. 9. Dysregulation of alternative splicing events in cerebellum from SCA3 mice

A SCA3 cerebellar datasets show widespread shared missplicing of skipped exon events in a repeat length dependent manner. All events shown for datasets pass threshold of either P<0.05 or FDR<0.1, grey squares indicate events with P>0.05. * *Itpa* exon 4 inclusion event is specifically exon 4 with retention of 205bp of intron 4-5 as in transcript *Itpa*-203.

B-D Dysregulation of *Bcas1* exon 9 and exon 10 alternative splicing occurs across SCA3 cerebellar datasets; **B** *Bcas1* exon 10 in the context of exon 9, 10, 11; **C** *Bcas1* exon 10 in the context of exon 9, 10, 12; ns – not significant, *P<0.05, **FDR<0.1, line indicates median.

E, F *Robo 1* exon 18 and *Pex5l* exon 2 show dysregulation of alternative splicing across SCA3 cerebellar datasets; ns – not significant, *P<0.05, **FDR<0.1, line indicates median.

G Schematic of *Bcas1* indicating exon positions, the significantly misspliced skipped exons and the datasets that show missplicing of each skipped exon event.

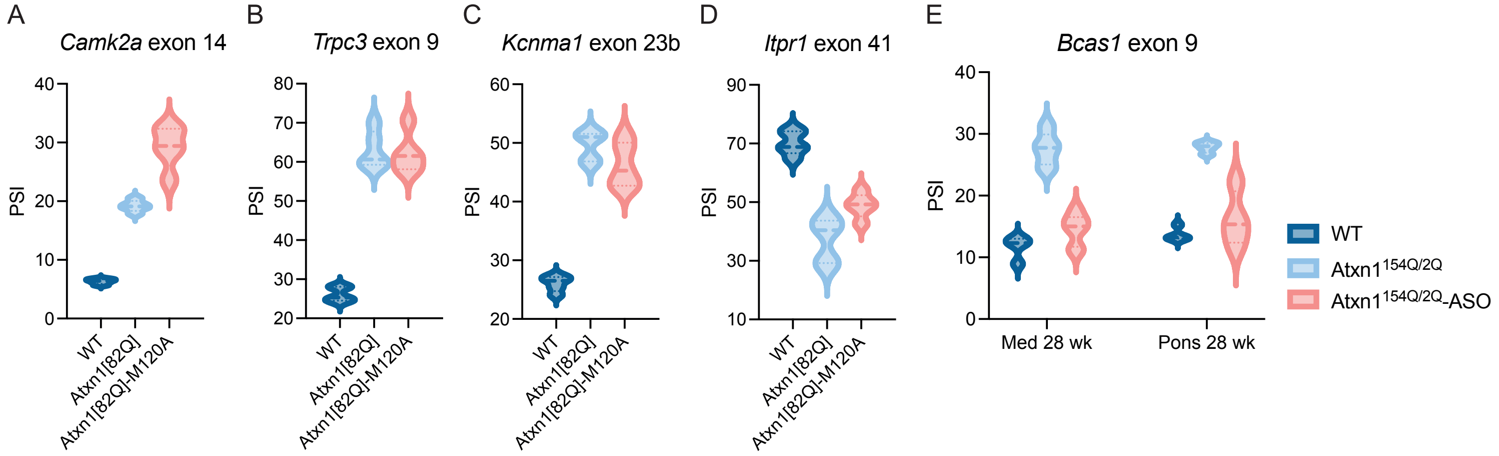

Supplementary Fig. 10. Alternative splicing dysregulation is rescued to different extents in therapeutic studies of CAG expansion SCAs

A ATXN1[82Q]-M120A mice show significant worsening of splicing of *Camk2a* exon 14; FDR<0.1, rescue >10%, PSI>5%.

B-D ATXN1[82Q]-M120A mice do not show significant rescue of *Trpc3* exon 9 or *Kcnma1* exon 23b and show minor rescue of *Itpr1* exon 41; **D** FDR<0.1, rescue>10%, PSI>5%.

E Atxn1 ASO treatment rescues *Bcas1* exon 9 missplicing in medulla and pons of *Atxn1*$^{154Q/2Q}$ mice; rescue>10%, PSI>5%, FDR<0.1; data shown for datasets with significant missplicing of *Bcas1* exon 9 at FDR<0.1, PSI>10% between WT and *Atxn1*$^{154Q/2Q}$ mice (see Supplementary Fig. 7D, F).

A-E Thick line indicates median, thin dotted lines indicate quartiles.

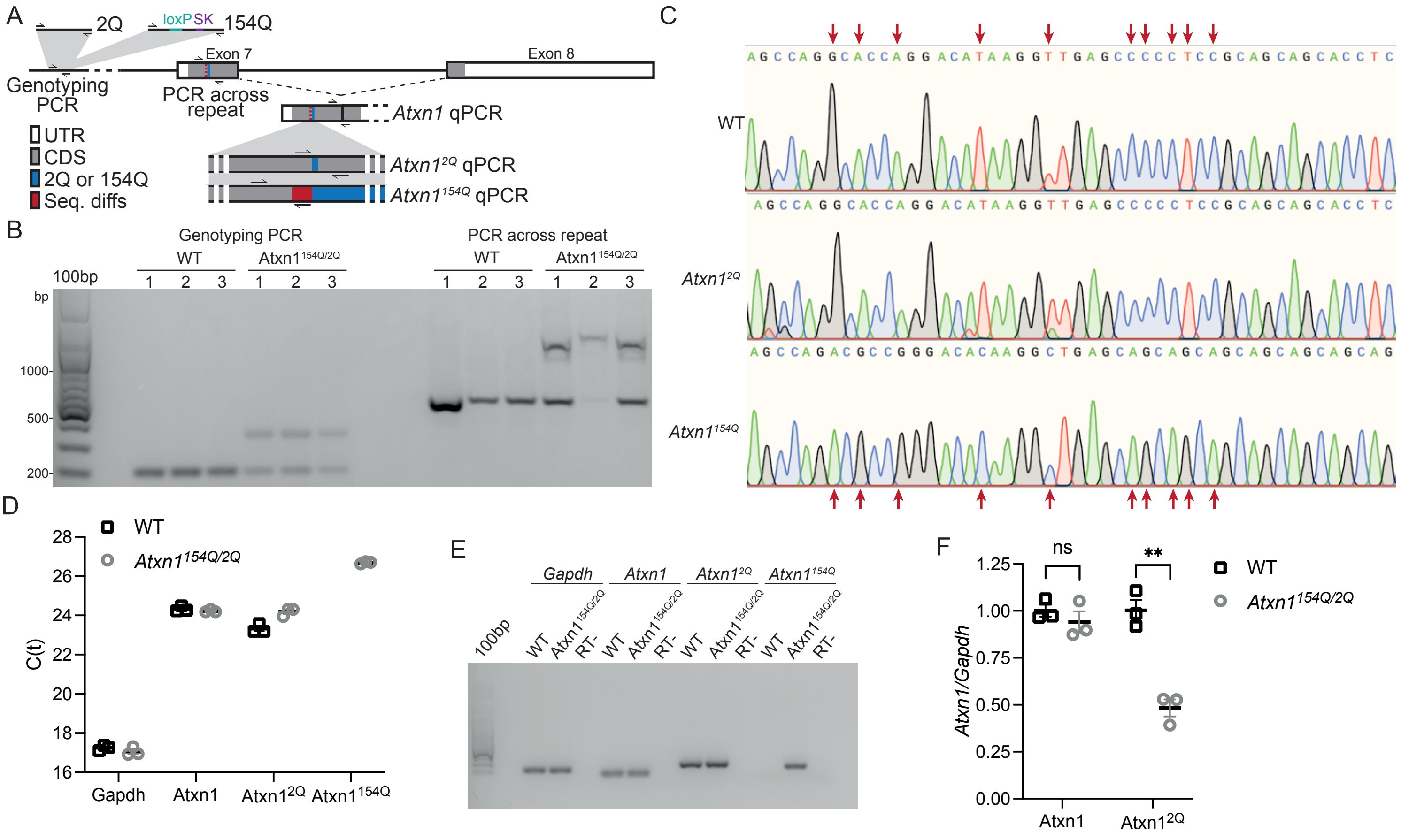

Supplementary Fig. 11. Quantification of allele specific *Atxn1* RNA levels

A Schematic of *Atxn1* intron 6-7, exon 7, intron 7-8 and exon 8 showing primers used for PCR and qPCR. Genotyping PCR primers: Jax labs standard PCR primers for strain 005601. The forward primer for the *Atxn1*^{2Q} selective qPCR and the reverse primer for the *Atxn1*^{154Q} selective qPCR bind to the same region which contains sequence differences in the *Atxn1*^{154Q} allele. White with black box: untranslated region (UTR); Grey: coding sequence (CDS); Blue: 2Q or 154Q repeat tract; Red: sequence differences in *Atxn1*^{154Q} allele (see panel C). Exon numbers and relative exon/intron sizes are derived from ensembl m38 genome *Atxn1* transcript 201.

B Representative genotyping gel for conventional genotyping PCR and PCR across the repeat for *Atxn1*$^{154Q/2Q}$ mice.

C Sanger sequencing confirming mutations associated with the 154Q allele for design of 2Q and 154Q allele selective qPCR primers. Red arrows indicate sequence differences between the 154Q and 2Q alleles. Sequence differences in the 154Q allele are consistent with the human ATXN1 sequence.

D Cycle threshold [C(t)] values for qPCR using primers specific to *Gapdh*, *Atxn1* (non-allele selective), *Atxn1*^{2Q} and *Atxn1*^{154Q}.

E Representative agarose gel showing qPCR products from **D** demonstrating a single product and no signal for RT- reactions for each primer set and no signal for the WT mouse with *Atxn1*^{154Q} allele selective primers.

F Non-allele selective qPCR primers detect no difference in Atxn1 RNA levels between WT and *Atxn1*$^{154Q/2Q}$ mice; allele selective *Atxn1*^{2Q} qPCR primers detect 50% less *Atxn1*^{2Q} in *Atxn1*$^{154Q/2Q}$ mice compared to WT mice as expected; ns – not significant, ** P<0.01.
