# Supplementary material for: Widespread alternative splicing dysregulation occurs presymptomatically in CAG expansion spinocerebellar ataxias
Source: Brain. 2023 Sep 30;147(2):486–504. doi: 10.1093/brain/awad329 (PMC10834251; doi:10.1093/brain/awad329)

Uncropped agarose gel for Supplementary Figure 8A

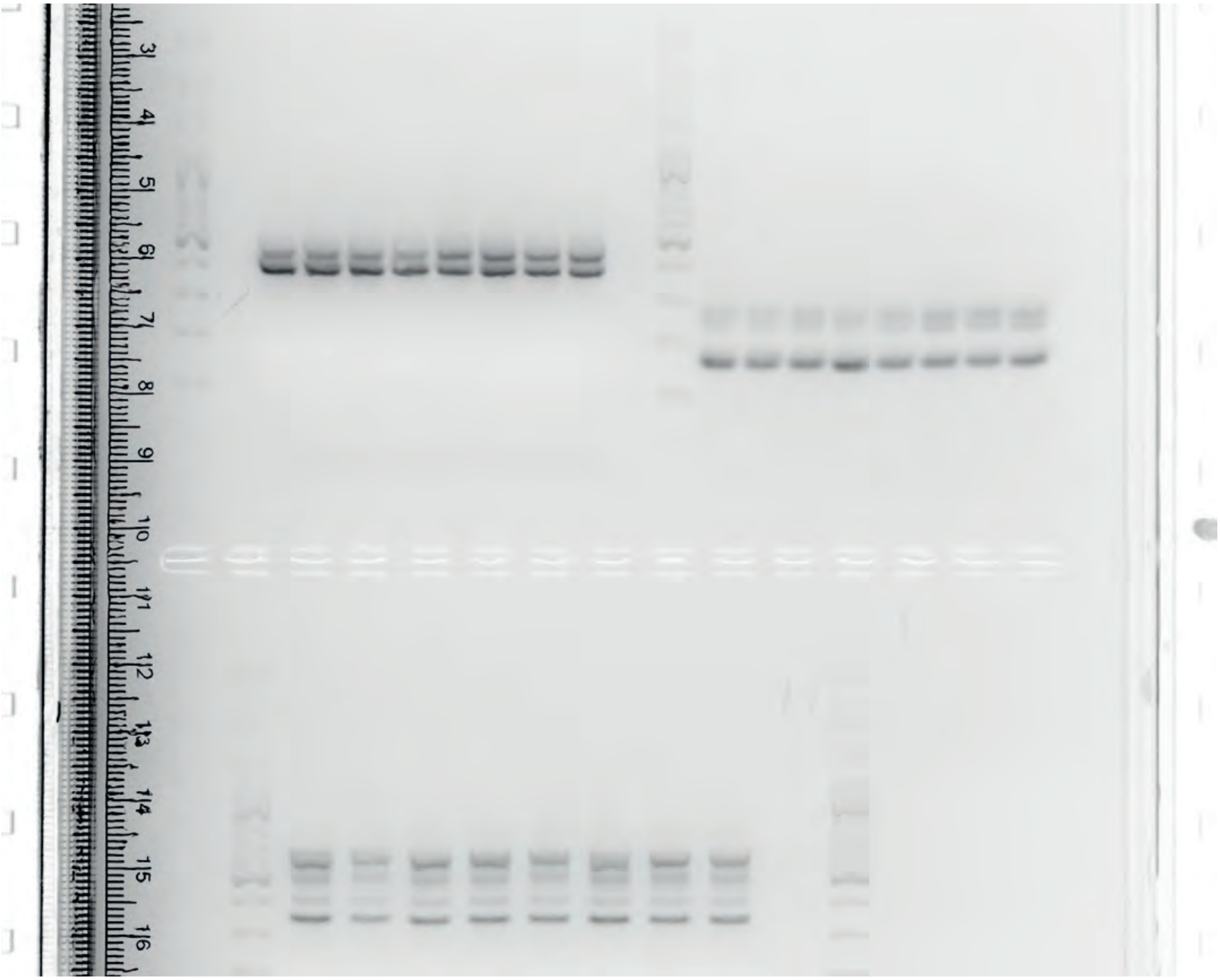

Uncropped agarose gel for Supplementary Figures 8B and C

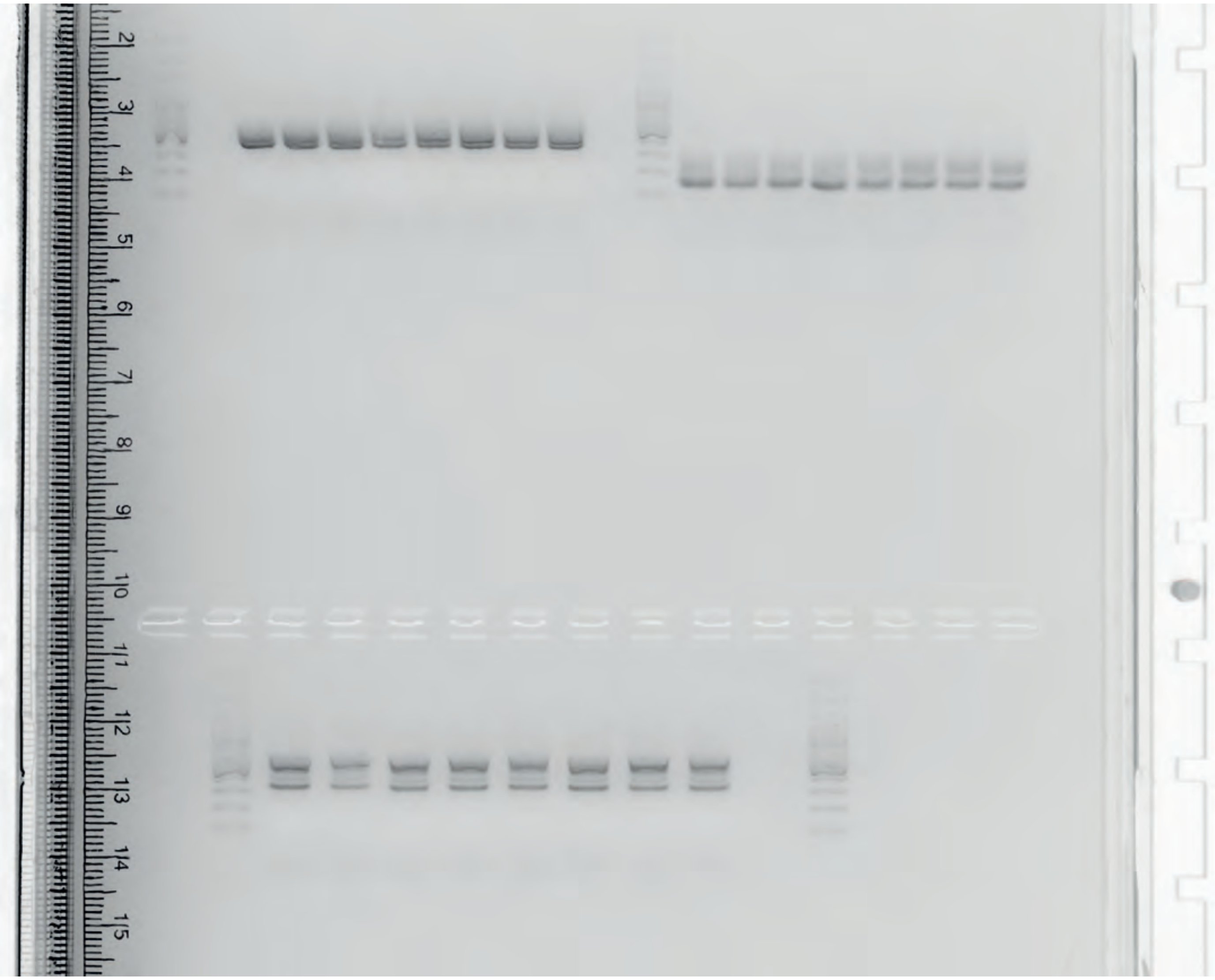

Uncropped agarose gel for Supplementary Figure 11B

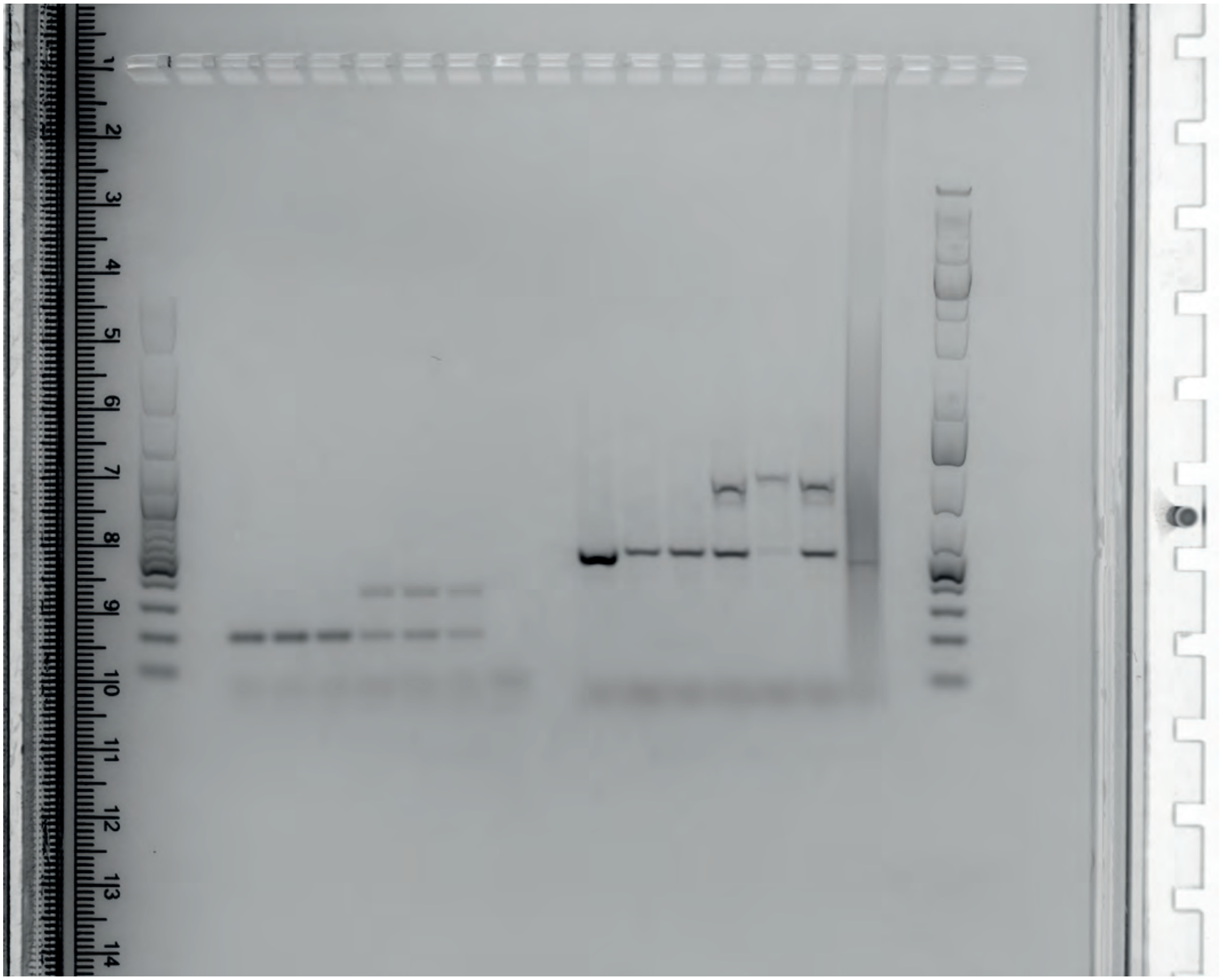

Uncropped agarose gel for Supplementary Figure 11E

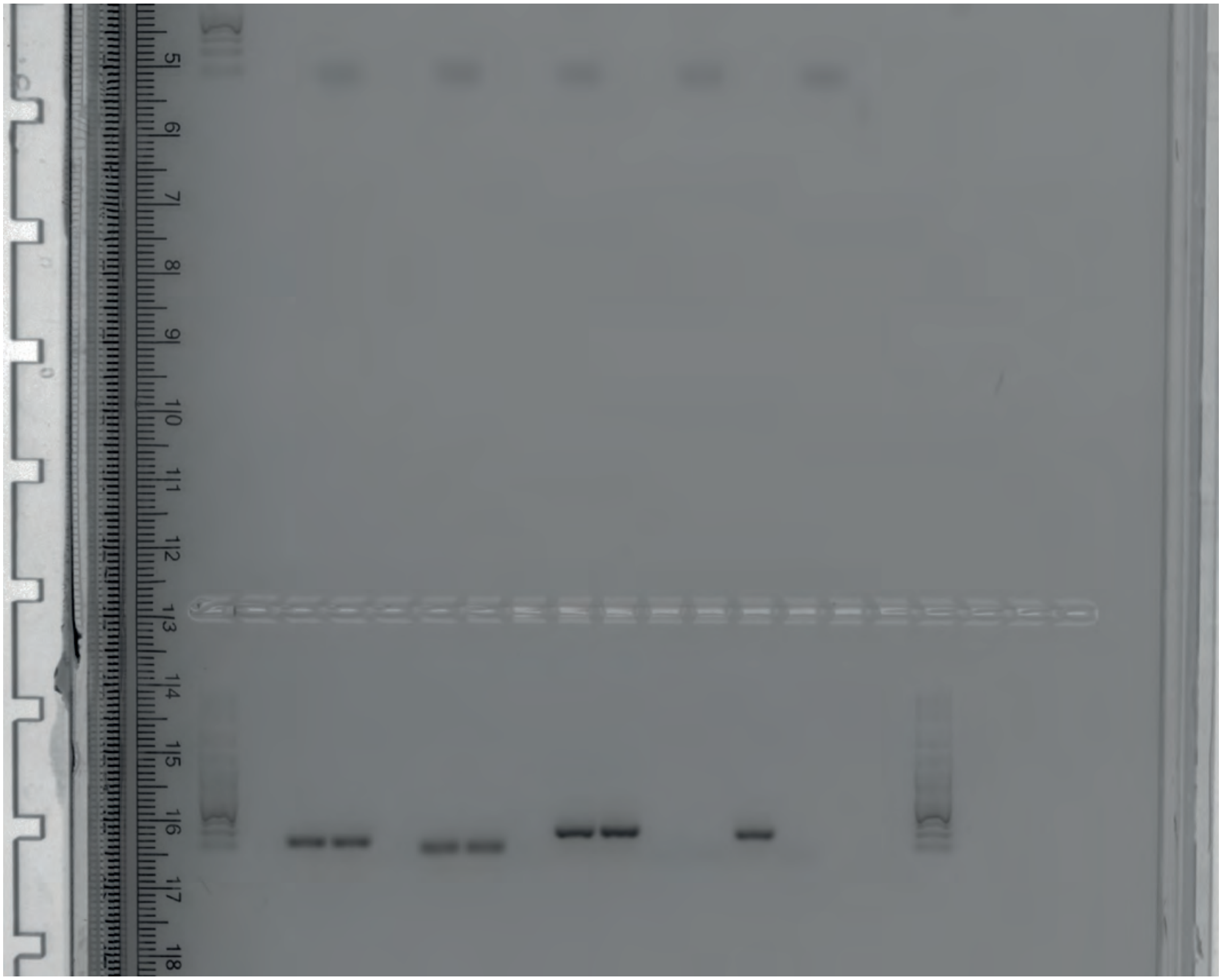

Supplement: awad329_Supplementary_Data [file awad329_supplementary_data.zip › brain-2023-01025-File019.pdf]
